# FoxO1/Rictor axis induces a nongenetic adaptation to ibrutinib via Akt activation in chronic lymphocytic leukemia

**DOI:** 10.1172/JCI173770

**Published:** 2024-10-22

**Authors:** Laura Ondrisova, Vaclav Seda, Krystof Hlavac, Petra Pavelkova, Eva Hoferkova, Giorgia Chiodin, Lenka Kostalova, Gabriela Mladonicka Pavlasova, Daniel Filip, Josef Vecera, Pedro Faria Zeni, Jan Oppelt, Zuzana Kahounova, Rachel Vichova, Karel Soucek, Anna Panovska, Karla Plevova, Sarka Pospisilova, Martin Simkovic, Filip Vrbacky, Daniel Lysak, Stacey M. Fernandes, Matthew S. Davids, Alba Maiques-Diaz, Stella Charalampopoulou, Jose I. Martin-Subero, Jennifer R. Brown, Michael Doubek, Francesco Forconi, Jiri Mayer, Marek Mraz

**Affiliations:** 1 Molecular Medicine, CEITEC Masaryk University, Brno, Czechia.; 2 Department of Internal Medicine, Hematology and Oncology, University Hospital Brno and Faculty of Medicine, Masaryk University, Brno, Czechia.; 3 Cancer Sciences, Faculty of Medicine, University of Southampton, Southampton, United Kingdom.; 4 Department of Cytokinetics, Institute of Biophysics of the Czech Academy of Sciences, Brno, Czechia.; 5 Fourth Department of Internal Medicine–Haematology, University Hospital Hradec Kralove and Faculty of Medicine Hradec Kralove, Charles University, Prague, Czechia.; 6 Department of Haematology and Oncology, University Hospital Pilsen, Pilsen, Czechia.; 7 Department of Medical Oncology, Dana-Farber Cancer Institute, Boston, Massachusetts, USA.; 8 Institut d’Investigacions Biomèdiques August Pi I Sunyer (IDIBAPS), University of Barcelona, Barcelona, Spain.; 9 Haematology Department, Cancer Care Directorate, University Hospital Southampton NHS Trust, Southampton, United Kingdom.

**Keywords:** Hematology, Oncology, Drug therapy, Leukemias, Signal transduction

## Abstract

Bruton tyrosine kinase (BTK) inhibitor therapy induces peripheral blood lymphocytosis in chronic lymphocytic leukemia (CLL), which lasts for several months. It remains unclear whether nongenetic adaptation mechanisms exist, allowing CLL cells’ survival during BTK inhibitor–induced lymphocytosis and/or playing a role in therapy resistance. We show that in approximately 70% of CLL cases, ibrutinib treatment in vivo increases Akt activity above pretherapy levels within several weeks, leading to compensatory CLL cell survival and a more prominent lymphocytosis on therapy. Ibrutinib-induced Akt phosphorylation (pAkt^S473^) is caused by the upregulation of Forkhead box protein O1 (FoxO1) transcription factor, which induces expression of *Rictor*, an assembly protein for the mTORC2 protein complex that directly phosphorylates Akt at serine 473 (S473). Knockout or inhibition of FoxO1 or Rictor led to a dramatic decrease in Akt phosphorylation and growth disadvantage for malignant B cells in the presence of ibrutinib (or PI3K inhibitor idelalisib) in vitro and in vivo. The FoxO1/Rictor/pAkt^S473^ axis represents an early nongenetic adaptation to B cell receptor (BCR) inhibitor therapy not requiring PI3Kδ or BTK kinase activity. We further demonstrate that FoxO1 can be targeted therapeutically and its inhibition induces CLL cells’ apoptosis alone or in combination with BTK inhibitors (ibrutinib, acalabrutinib, pirtobrutinib) and blocks their proliferation triggered by T cell factors (CD40L, IL-4, and IL-21).

## Abstract 2

We describe a non-genetic adaptation to BTK inhibitor therapy in chronic lymphocytic leukemia that involves Akt activation via FoxO1/Rictor axis.

## Graphical abstract

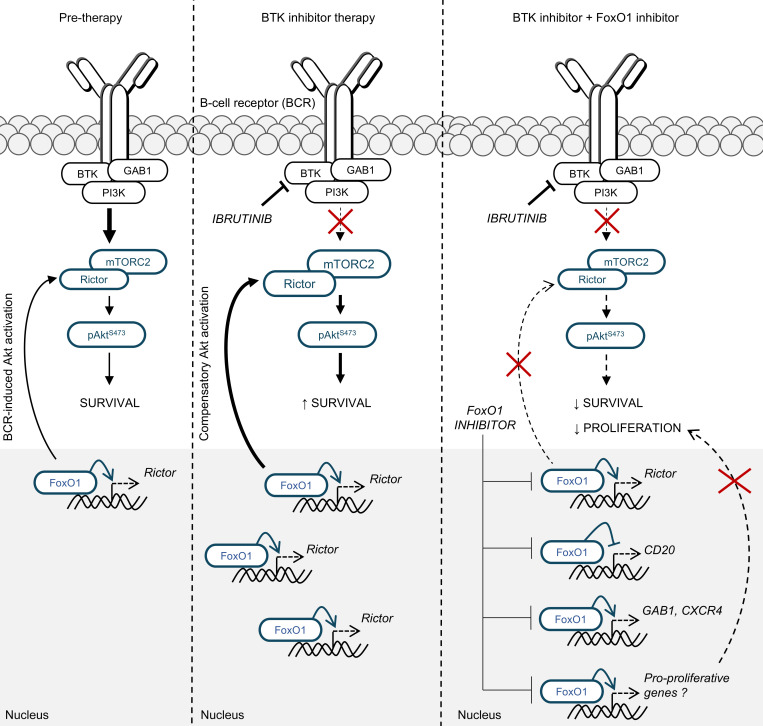

## Introduction

The approval of Bruton tyrosine kinase (BTK) inhibitor ibrutinib and PI3K inhibitor idelalisib has revolutionized chronic lymphocytic leukemia (CLL) therapy (1–4). These B cell receptor (BCR) inhibitors induce transient peripheral blood lymphocytosis in CLL lasting for several months by inhibiting CLL cells’ adhesion and chemokine responsiveness (5–7). This was an unexpected consequence of BTK or PI3K inhibition and is coupled with the relatively weak induction of CLL cell death by these drugs. Resistance to ibrutinib has been linked to *BTK* and/or *PLCG2* mutations and several chromosomal aberrations (8–11). However, these mutations occur late on therapy (typically >12 months) and cannot explain why CLL cells initially undergo apoptosis at a slow rate, with the lymphocytosis lasting for several months, allowing for a large pool of malignant B cells that can later develop resistance (1–3, 12). Notably, increased CLL cell resistance to apoptosis during the period of lymphocytosis has been associated with higher levels of minimal residual disease (13).

We hypothesized that early nongenetic adaptation mechanisms to BCR inhibitors might exist, allowing CLL cells to survive the lack of recirculation to immune niches and microenvironmental interactions that provide them with antiapoptotic signals. Nongenetic mechanisms of resistance or adaptation to ibrutinib have been described in mantle cell lymphoma (MCL) and diffuse large B cell lymphoma (DLBCL) and involve CD79b overexpression or compensatory PI3K activation (14–17). Nonmutational mechanisms of resistance/adaptation have also been described with other kinase inhibitors, such as BRAF or ALK inhibitors (melanoma, lung cancer) (18–22). In CLL, we have previously described ibrutinib therapy as inducing cell-surface IgM and BCR adaptor protein GAB1, suggesting a compensatory response (23–25), and rewiring of the signaling pathways upon ibrutinib treatment has also been shown by others (26). CLL provides a unique opportunity to sample primary malignant cells during targeted therapy to study “compensatory” signaling pathway activation. We focused on the possible role of the Akt pathway in adapting to BCR inhibitors since, in mouse models, PI3K/Akt activation rescues the apoptosis induced by complete loss of BCR signaling via deletion of cell-surface BCR in mature B cells, which could be seen as parallel to inhibiting an essential BCR-associated kinase. Inducing PI3K/Akt represents a known rescue mechanism for BCR loss in normal mature B cells, and activating NF-κB, MAPK/ERK, or Bcl2 is not sufficient to prevent cell apoptosis (27, 28). Moreover, Akt activity has been identified as a critical prosurvival signal in CLL and mature B cells (28–33).

Here, we describe an early adaptation mechanism by which CLL cells increase Akt phosphorylation during in vivo ibrutinib therapy. This supports CLL cell survival and is clinically associated with more prominent ibrutinib-induced lymphocytosis during the first months of therapy. We describe for the first time, to our knowledge, that FoxO1 acts as a direct transcriptional activator of Rictor during BTK inhibitor therapy, leading to mTORC2-mediated phosphorylation of Akt (S473) and thus contributing to the survival of CLL cells. We also show that inhibition of FoxO1 decreases CLL cell viability and reduces CLL cell proliferation induced by T cell signals, suggesting a potential therapeutic target.

## Results

### Akt phosphorylation is induced during ibrutinib therapy in vivo.

**Figure 1:**
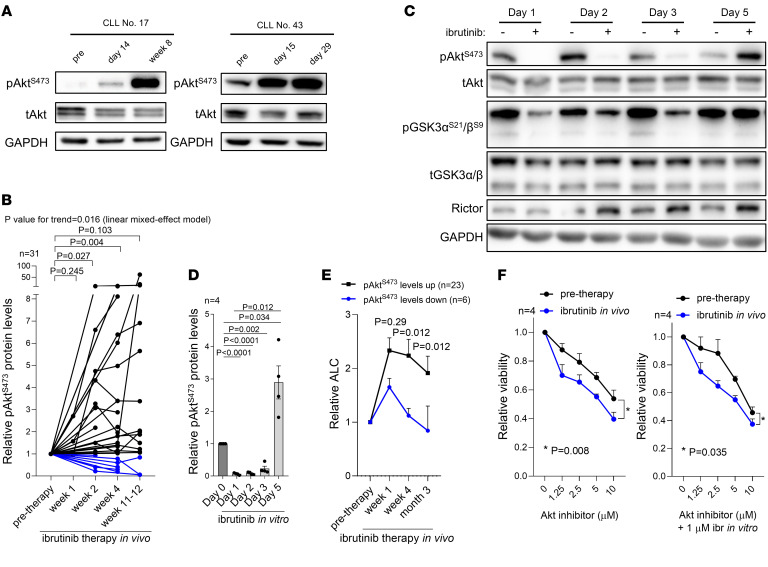
CLL cells induce Akt phosphorylation during ibrutinib treatment. (**A**) Representative pAkt^S473^ immunoblots in primary CLL samples (*n* = 2) obtained before and during ibrutinib treatment in vivo. (**B**) Densitometric quantification of relative pAkt^S473^ protein levels in primary CLL samples obtained before and during ibrutinib treatment in vivo (*n* = 31). *P* value for trend calculated with linear mixed-effect model; other *P* values were calculated using paired *t* test. Black lines indicate samples with pAkt^S473^ upregulation (*n* = 22) or stable levels (*n* = 2); blue lines indicate samples with pAkt^S473^ downregulation (*n* = 7). The pAkt^S473^ levels were normalized to both total Akt and GAPDH (loading control) to obtain precise quantification. For patient characteristics, see Supplemental Table 8. (**C**) Representative immunoblot of MEC1 cells treated with ibrutinib (2 μM, 1–5 days). (**D**) Densitometric quantification of relative pAkt^S473^ protein levels in MEC1 cells treated with ibrutinib (2 μM) for 1–5 days (*n* = 4). The pAkt^S473^ levels at day 0 were set as 1. Fresh ibrutinib was added to culture media each day. *P* values were calculated using paired *t* test. (**E**) Relative absolute lymphocyte count (ALC) in patients treated with ibrutinib in vivo that had upregulated/stable (*n* = 23) or downregulated (*n* = 6) levels of pAkt^S473^ in the first 12 weeks of the therapy (stratification according to **B**). All samples with available clinical data were analyzed. *P* values were calculated using Mann-Whitney *U* test. (**F**) Relative viability of CLL samples (*n* = 4) obtained from patients before and after 4 weeks (*n* = 2), 6 weeks (*n* = 1), or 8 weeks (*n* = 1) of ibrutinib therapy in vivo and treated (72 hours) with Akt inhibitor MK-2206 (1.25, 2.5, 5, and 10 μM) or a combination of ibrutinib (ibr, 1 μM) and MK-2206 (1.25, 2.5, 5, and 10 μM), or vehicle. Statistical difference was tested using 2-way ANOVA with Geisser-Greenhouse correction. For patient characteristics, see Supplemental Table 8.

We hypothesized that CLL cells with inhibited BTK might adapt via compensatory Akt activation since PI3K/Akt represents a known rescue mechanism for BCR loss in normal mature B cells (27, 28). Indeed, we observed that primary CLL cells obtained from patients treated continuously with ibrutinib in vivo gain Akt phosphorylation on serine 473 (pAkt^S473^) within several weeks on the therapy (in total 31 patients with 87 samples; fold-change 3.2 at week 4, *P* = 0.004; Figure 1, A and B). The induction of Akt^S473^ phosphorylation in these samples obtained at the time of ibrutinib-induced lymphocytosis exceeded the pretherapy levels in approximately 70% of cases, and an additional approximately 6% of cases had stable pAkt^S473^ levels (*n* = 22 out of 31 induced pAkt^S473^, 2 patients had stable pAkt^S473^ levels; Figure 1, A and B). This is independent of major clinicobiological CLL characteristics, such as immunoglobulin heavy chain variable region gene (IGHV) status or the presence of recurrent chromosomal aberrations (Supplemental Figure 1, A and B; supplemental material available online with this article; https://doi.org/10.1172/JCI173770DS1), and is not related to *BTK/PLCG2* mutation evolution, since these are not present in a detectable fraction of CLL cells early during therapy (also see below). On the contrary, the T308 phosphorylation of Akt was not significantly induced during ibrutinib therapy (*n* = 20; Supplemental Figure 1C). The pAkt^S473^ was restored in MEC1 cells treated with ibrutinib in vitro, where after an initial drop in Akt phosphorylation, its levels were induced within 5 days (fold-change ~40 between day 1 versus day 5, *P* = 0.012; Figure 1, C and D), allowing us to study this phenotype in a CLL-derived cell line. This process was not due to a selection of apoptosis-resistant MEC1 cells, since ibrutinib did not cause apoptosis under the used conditions (2 μM ibrutinib, Supplemental Figure 2). Akt phosphorylation induction was reflected in its increased activity in cells exposed to ibrutinib, namely Akt-mediated inhibitory phosphorylation of GSK3α^S21^/β^S9^ (Figure 1C; day 5). Importantly, patients with upregulated pAkt^S473^ levels upon ibrutinib therapy had a more prominent and longer lasting lymphocytosis compared with those with downregulated pAkt^S473^ levels (*P* = 0.012, month 1 and 3; Figure 1E and Supplemental Figure 1D). Furthermore, CLL cells were highly sensitive to the Akt inhibitor and more sensitive when obtained during ibrutinib treatment in vivo (*P* < 0.05; Figure 1F). Notably, Akt was also phosphorylated (S473) at the time of relapse on BTK inhibitors in several analyzed samples with clonal *BTK* mutations (Supplemental Figure 3). Altogether, this shows that CLL cells can relatively rapidly activate Akt phosphorylation independently of BTK kinase activity, which correlates with the extent of ibrutinib-induced lymphocytosis and supports CLL cell survival.

### Upregulation of Rictor/mTORC2 complex induces Akt phosphorylation during ibrutinib therapy.

**Figure 2:**
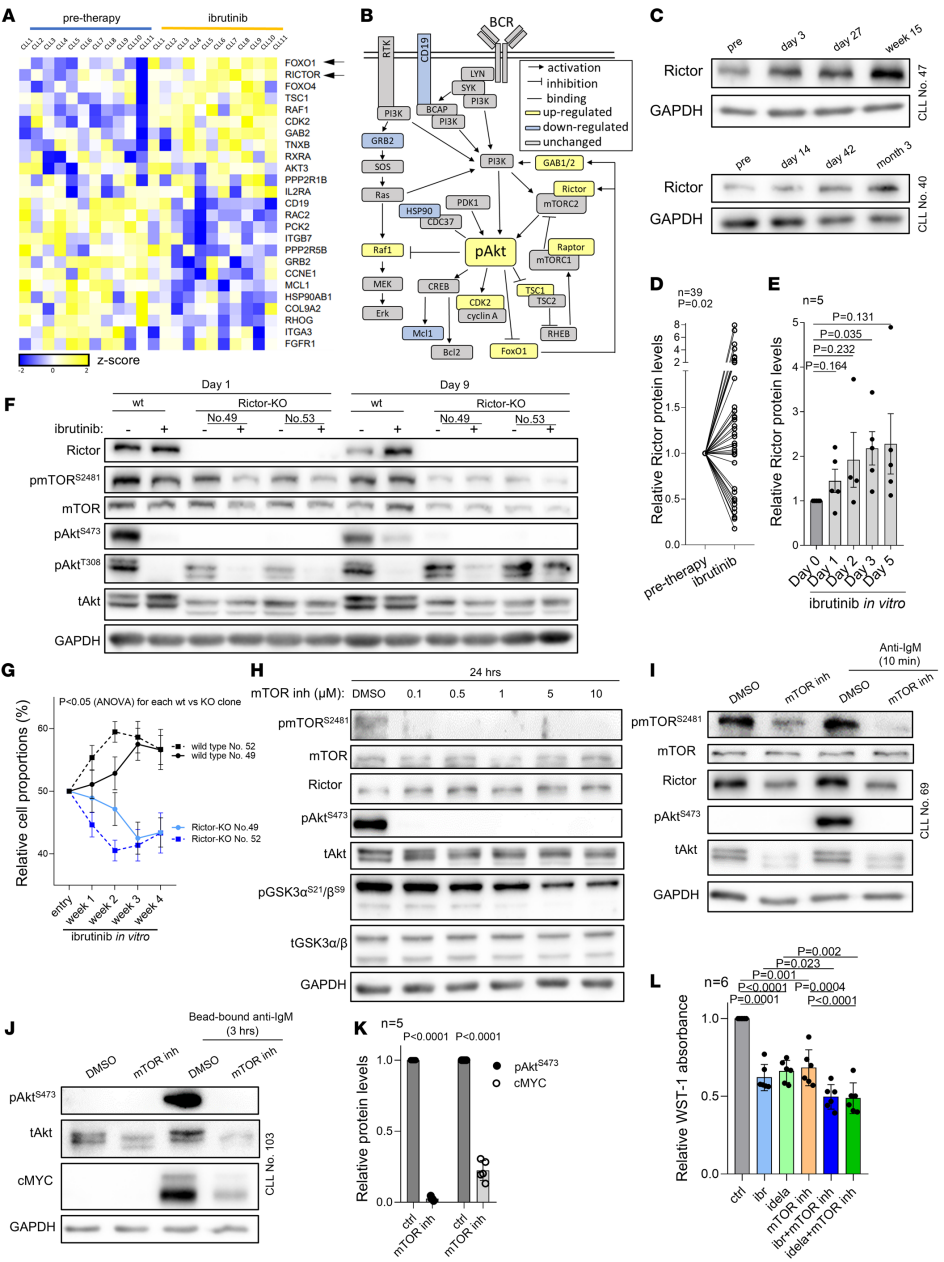
Induction in Akt activity during ibrutinib treatment is caused by Rictor upregulation. (**A**) Heatmap of differentially expressed genes from samples obtained before and during ibrutinib treatment in vivo (*P* adj < 0.05; base mean > 100; *n* = 11 pairs). List includes genes overlapping with genes involved in PI3K/Akt signaling (gene sets: no. M27162, reactome_PI3K_AKT_signalling _in_cancer and has04151, KEGG: PI3K-Akt signaling pathway). (**B**) PI3K/Akt pathway with visualization of genes differentially expressed in patients treated with ibrutinib in vivo. (**C**) Representative immunoblots of Rictor in primary CLL samples obtained before and during ibrutinib treatment in vivo (*n* = 2). (**D**) Densitometric quantification of Rictor protein levels in primary CLL samples obtained before and during ibrutinib treatment in vivo for 2–8 weeks (*n* = 39). (**E**) Relative Rictor protein levels analyzed by densitometric quantification of immunoblots in MEC1 cells treated with ibrutinib (2 μM) for 5 days (*n* = 5; representative immunoblot in Figure 1C). (**F**) Representative immunoblot of WT and Rictor-KO MEC1 clones (*Rictor*-KO). Cells treated with ibrutinib (2 μM) for 1 or 9 days. (**G**) Competitive growth of WT versus *Rictor*-KO MEC1 cells. (*n* = 4 repetitions for each of the 2 clones, WT clones are marked by numbers corresponding to the specific KO clone that was used in the corresponding competitive growth experiment). Cells were treated with ibrutinib (2 μM, fresh ibrutinib was added 3 times a week) or vehicle (DMSO). Graph represents percentage of KO versus WT ibrutinib-treated cells, and this is plotted relative to vehicle-treated (DMSO) KO or WT cells, respectively, to correct for any effect of the KO on ibrutinib-unrelated cell fitness. Statistical difference was tested using 2-way ANOVA with Geisser-Greenhouse correction. (**H**) Representative immunoblot of MEC1 cells treated with mTOR inhibitor AZD8055 (0.1–10 μM, 24 hours). (**I**) Representative immunoblot of primary CLL cells treated with mTOR inhibitor AZD8055 (0.5 μM) for 24 hours and then stimulated with anti-IgM (20 μg/ml) for 10 minutes. (**J**) Representative immunoblot of primary CLL cells treated with mTOR inhibitor AZD8055 (0.5 μM) for 24 hours and then stimulated with bead-bound anti-IgM for 3 hours. (**K**) Relative protein levels of pAkt^S473^ and cMYC obtained by densitometric quantification of immunoblots from the experiment described in **J** (*n* = 5). (**L**) Relative viability (WST-1 absorbance) in CLL cells (*n* = 6) treated with ibrutinib (ibr, 1 μM), idelalisib (idela, 1 μM), AZD8055 (mTOR inh, 0.5 μM), or their combination (48 hours). *P* values in **D**, **E**, **K**, and **L** were calculated using paired *t* test. For patient characteristics, see Supplemental Table 8.

To identify the factors responsible for Akt phosphorylation, we performed a transcriptome analysis (RNA-Seq) of paired samples (*n* = 11) obtained from patients before and during ibrutinib treatment in vivo (median time on therapy, 2 weeks; range, 1–12 weeks; all samples had WT *BTK/PLCG2* at both analyzed time points). This identified 1,034 differentially expressed genes during ibrutinib treatment (fold-change >1.5, *P* adjusted < 0.05; Supplemental Table 1). As expected, Gene Ontology analysis revealed changes in cell adhesion, BCR signaling, immune response, and other related pathways (Supplemental Table 2). Notably, the differentially expressed mRNAs included 25 genes (12 upregulated, 13 downregulated) annotated in databases as involved in the PI3K/Akt pathway (Figure 2, A and B, and Supplemental Table 3). Rictor induction on ibrutinib was particularly notable since it is an essential assembly protein for the mTORC2 complex, which is known to directly phosphorylate Akt on S473 (34). Rictor upregulation was further confirmed at a protein level in 25 out of 39 patients (64.1%) treated with ibrutinib in vivo (*P* = 0.02; Figure 2, C and D) as well as in ibrutinib-treated MEC1 cells (from day 3, fold-change, ~2.2, *P* = 0.035; Figure 1C and Figure 2E). This led us to hypothesize that higher Rictor levels during ibrutinib therapy are responsible for increased mTORC2 activity and Akt^S473^ phosphorylation. Indeed, *Rictor* KO in MEC1 cells resulted in a dramatic decrease in pAkt^S473^ and phosphorylated mTOR (phospho-mTOR) levels (Figure 2F), and these cells were unable to recover pAkt^S473^ levels after ibrutinib treatment in vitro (Figure 2F). This demonstrates that the Rictor level determines the Rictor/mTORC2 complex’s ability to maintain basal pAkt^S473^ levels. Importantly, *Rictor* KO cells had a significant growth disadvantage during competitive culture in the presence of ibrutinib (4 weeks) compared with control MEC1 WT cells (Figure 2G). In line with these observations, mTOR inhibitor AZD8055 (specific Rictor inhibitor is not available) also inhibited Akt phosphorylation in WT MEC1 cells (Figure 2H). mTOR inhibition also completely blocked Akt^S473^ phosphorylation in primary CLL cells induced by short-term (soluble anti-IgM) and long-term (bead-bound anti-IgM) BCR crosslinking, a known mTORC1/2 pathway activator (Figure 2, I, J, and K), and significantly impaired cMYC induction upon long-term BCR stimulation by bead-bound anti-IgM (Figure 2, J and K). The combination of mTOR inhibitor with ibrutinib or idelalisib was more toxic (*P* < 0.05) to primary CLL cells than each BCR inhibitor alone (Figure 2L). Altogether, these data indicate that induction of Rictor, a key assembly protein of the mTORC2 complex, is responsible for the basal, BCR-induced, and compensatory Akt^S473^ phosphorylation during ibrutinib therapy.

### FoxO1 activates Rictor/pAkt^S473^ axis during BCR inhibitor treatment.

**Figure 3:**
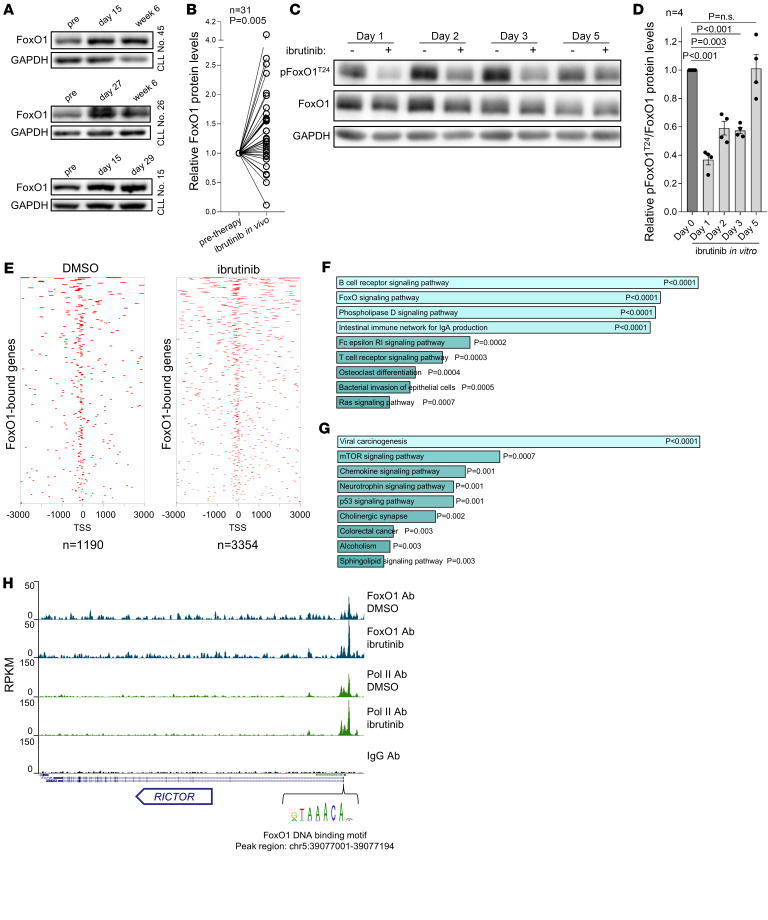
FoxO1 is more active in ibrutinib-treated CLL cells and binds to *RICTOR* promoter. (**A**) Representative immunoblots of FoxO1 in primary CLL samples obtained before and during ibrutinib therapy in vivo (*n* = 3). (**B**) Densitometric quantification of relative FoxO1 protein levels (immunoblots) in primary CLL samples obtained before and during ibrutinib therapy in vivo (2–8 weeks, *n* = 31). *P* value was calculated using paired *t* test. For patient characteristics, see Supplemental Table 8. (**C**) Representative immunoblot of MEC1 cells treated with 2 μM ibrutinib (1–5 days). The immunoblot is from the same samples as in Figure 1C to allow a comparison. (**D**) Densitometric quantification of relative pFoxO1^T24^/FoxO1 protein levels analyzed by immunoblot in MEC1 cells treated with 2 μM ibrutinib for 1–5 days (*n* = 4). FoxO1^T24^ phosphorylation inhibits FoxO1 functions in the nucleus. Fresh ibrutinib was added to culture media each day. *P* values were calculated using paired *t* test. (**E**) Heatmap of FoxO1 binding to transcription start site (TSS) regions containing its binding motif in MEC1 cells treated 6 days with vehicle (DMSO) or ibrutinib (1 μM). (**F** and **G**) Pathway enrichment analysis of FoxO1-bound genes in MEC1 cells treated with ibrutinib (1 μM) or vehicle (6 days). (**F**) Pathway enrichment analysis (Enrichr tool) of FoxO1-bound genes overlapping between vehicle (DMSO) and ibrutinib-treated MEC1 cells. (**G**) Pathway enrichment analysis (Enrichr tool) of genes bound by FoxO1 preferentially in ibrutinib-treated MEC1 cells compared with vehicle -treated (DMSO) cells. *P* values were calculated by Enrichr tool using Fisher’s exact test. (**H**) FoxO1 binding to *Rictor* promoter region in MEC1 cells treated with vehicle (DMSO) or ibrutinib (1 μM) for 6 days revealing increased reads per kb per transcript per million reads mapped (RPKM) in ibrutinib-treated cells. FoxO1 Ab represents pull-down with anti-FoxO1 antibody, Pol II Ab represents pull-down with anti-polymerase II antibody (serves as control), and IgG Ab represents pull-down with control IgG antibody (negative control).

The RNA-Seq of in vivo ibrutinib-exposed CLL cells revealed *FoxO1* induction (Figure 2A), and this attracted our attention, since FoxO1 has been shown to transcriptionally activate *Rictor* in renal cancer cells (35). Moreover, *Rictor* contains putative FoxO1 binding sites in its promoter (35, 36), and this led us to hypothesize that FoxO1 can contribute to *Rictor* upregulation in CLL. We have also noticed that other previously characterized FoxO1 transcriptional targets, such as *CXCR4*, *CD20*, *P27*, *BACH2*, or *NF1* (7, 37, 38), are changed during ibrutinib treatment (Supplemental Table 4) (7, 23). The FoxO1 protein levels increased during ibrutinib treatment in vivo in approximately 75 % of cases (*n* = 23 out of 31 patients, fold-change, ~1.4, *P* = 0.005; Figure 3, A and B), and patients who had more prominently upregulated FoxO1 levels also had significantly higher Rictor protein levels (Supplemental Figure 4). The extent of FoxO1 induction was not associated with CLL clinicobiological features such as IGHV status or the presence of chromosomal abnormalities (del17p13, del11q22, del13q14, trisomy 12; data not shown). Increased FoxO1 activity was also observed in ibrutinib-treated MEC1 cells as evidenced by a decrease in levels of inhibitory FoxO1^T24^ phosphorylation (fold-change, ~0.5, *P* < 0.005; Figure 3, C and D), and this preceded the induction of Akt phosphorylation. To study FoxO1 activity during ibrutinib therapy, we performed CUT&RUN experiments to directly assess genome-wide FoxO1 binding to DNA in MEC1 cells with and without ibrutinib treatment in vitro. This revealed clearly increased FoxO1 DNA binding activity in ibrutinib-treated MEC1 cells with 3,354 regions being bound by FoxO1 and containing its canonical DNA binding motif as compared with 1,190 FoxO1-bound regions in vehicle-treated MEC1 cells (Figure 3E). BCR signaling was the most enriched pathway among FoxO1-bound genes in both conditions (vehicle and ibrutinib; *P* < 0.0001), while mTOR signaling was specifically enriched among genes preferentially bound by FoxO1 in ibrutinib-treated cells (*P* < 0.001; Figure 3, F and G). Notably, the FoxO1 targets identified by CUT&RUN in ibrutinib-treated MEC1 cells included 7 out of 25 PI3K/Akt pathway regulators changed during ibrutinib therapy in vivo including *Rictor* (Figure 2A, Figure 3H, and Supplemental Figure 5). The FoxO1 binding to *Rictor* was located close to its transcription start site, coincided with binding of polymerase II, and contained a well-known FoxO1 DNA-binding motif (as defined in ref. 39), suggesting a direct transcriptional regulation (Figure 3H).

**Figure 4:**
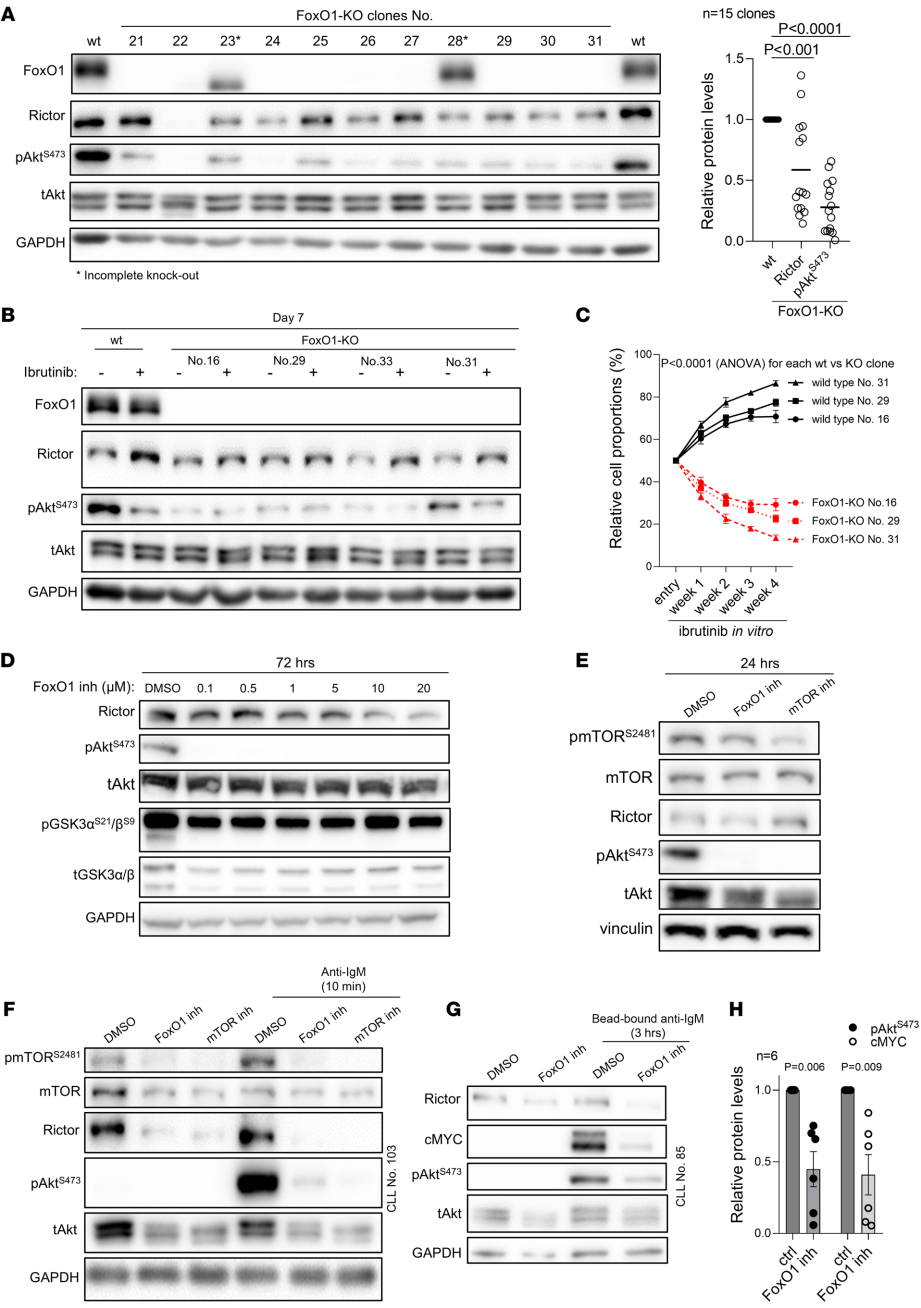
FoxO1-KO or inhibition leads to decrease of Rictor and pAkt^S473^ levels. (**A**) Representative immunoblot of *FoxO1*-KO MEC1 clones and densitometric quantification of relative Rictor and pAkt^S473^ protein levels in all obtained *FoxO1-*KO MEC1 clones with complete *FoxO1* KO (*n* = 15). *P* values were calculated using unpaired *t* test. (**B**) Representative immunoblot of WT and *FoxO1*-KO MEC1 clones (*n* = 4). Cells were treated with ibrutinib (1 μM) for 7 days. Fresh ibrutinib was added to culture media every other day. (**C**) Competitive growth of WT MEC1 cells versus *FoxO1*-KO MEC1 clones in medium with ibrutinib (2 μM, 4 weeks) relative to growth in medium with vehicle (DMSO). WT and FoxO1-KO cells marked with GFP or AZURIT (and vice versa) and mixed in 1:1 ratio (*n* = 4 repetitions for each of the 3 clones, WT clones are marked by numbers corresponding to the specific KO clone that was used in the corresponding competitive growth experiment). Cells were treated with ibrutinib (fresh ibrutinib was added 3 times a week) or DMSO. Graph represents the percentage of KO versus WT ibrutinib-treated cells, and this is plotted relatively to vehicle-treated (DMSO) KO or WT cells, respectively, to correct for any effect of the KO on ibrutinib-unrelated cell fitness. Statistical difference was tested using 2-way ANOVA with Geisser-Greenhouse correction. (**D**) Representative immunoblot of MEC1 cells treated with various FoxO1 inhibitor concentrations (72 hours). (**E**) Representative immunoblot of MEC1 cells treated with FoxO1 inhibitor (inh) or mTOR inhibitor (both 0.5 μM, 24 hours). (**F**) Representative immunoblot of primary CLL cells treated with FoxO1 inhibitor or mTOR inhibitor for 48 hours (both 0.5 μM) and then stimulated with anti-IgM (20 μg/ml, 10 minutes). (**G**) Representative immunoblot of primary CLL cells treated with FoxO1 inhibitor (0.5 μM) for 48 hours and then stimulated with bead-bound anti-IgM (3 hours). (**H**) Relative protein levels of pAkt^S473^ and cMYC obtained by densitometric quantification of immunoblots from experiment in **G** (*n* = 6). *P* values were calculated using paired *t* test.

Next, we produced 15 independent *FoxO1*-KO MEC1 clones (generated via single cell sorting of Cas9-sgRNA edited cells) and noted prominent Rictor and pAkt^S473^ downregulation (fold-change, 0.6 and 0.3, *P* < 0.001 and *P* < 0.0001, respectively; Figure 4A) as well as some pAkt^T308^ downregulation (Supplemental Figure 6A). *FoxO1*-KO cells were substantially less able to restore pAkt^S473^ levels during ibrutinib treatment (Figure 4B), and pAkt^S473^ levels were rescued in *FoxO1*-KO by artificial FoxO1 overexpression (Supplemental Figure 6, B and C). The *FoxO1*-KO in MEC1 cells dramatically decreased the fitness (*P* < 0.0001) of MEC1 cells treated with ibrutinib for 4 weeks in the competitive growth assay in comparison with *FoxO1* WT cells (Figure 4C). Moreover, *FoxO1*-KO MEC1 cells were completely outcompeted by WT cells in a competitive growth assay in immunodeficient NSG mice (Supplemental Figure 6D), confirming the important role of FoxO1 for malignant B cell growth. Treatment of MEC1 WT cells with FoxO1 inhibitor (AS1842856) resulted in Rictor downmodulation accompanied by pAkt^S473^ level repression (Figure 4, D and E). FoxO1 inhibitor also resulted in impaired signaling downstream of the crosslinked BCR, as evidenced by impaired pAkt^S473^ and cMYC induction in primary CLL cells (fold-change, 0.4 for both, *P* < 0.01; Figure 4, F–H), similarly to mTOR inhibitor. Overall, this demonstrates that FoxO1 plays an important role in activating the Rictor/pAkt^S473^ axis and is required for adaptation to ibrutinib.

Next, we tested to determine whether the FoxO1/Rictor/pAkt^S473^ pathway was activated not only in response to ibrutinib but also in response to acalabrutinib or pirtobrutinib. Indeed, FoxO1 and Rictor were also induced in response to acalabrutinib in vivo (*n* = 8 patients with 16 paired samples; fold-change, 1.3 and 2.1, respectively; *P* < 0.05; Supplemental Figure 7A) and pAkt^S473^ was induced in 4 out of 8 analyzed cases and stable in 1 additional case (Supplemental Figure 7A). In vitro, treatment of MEC1 cells with acalabrutinib or pirtobrutinib initially led to a decrease of Akt^S473^ phosphorylation, similarly to ibrutinib, followed by recovery of the FoxO1/Rictor/pAkt^S473^ axis (Supplemental Figure 7B). However, a stronger pAkt^S473^ repression was observed following acute ibrutinib treatment than acalabrutinib or pirtobrutinib treatment in BCR-stimulated cells in vitro (Supplemental Figure 7C). This is likely caused by ibrutinib off-target inhibition of other BCR-associated kinases such as LYN, BLK, or HCK (16, 40, 41). In line with this, FoxO1 and Rictor levels increased at day 5 of in vitro treatment of MEC1 with acalabrutinib or pirtobrutinib, but the pAkt^S473^ upregulation between day 1 and 5 was not as dramatic as with ibrutinib. Together, the results from samples obtained on acalabrutinib in vivo and the in vitro acalabrutinib/pirtobrutinib treatments confirm that changes in FoxO1 and Rictor are BTK dependent and contribute to pAkt^S473^ induction.

**Figure 5:**
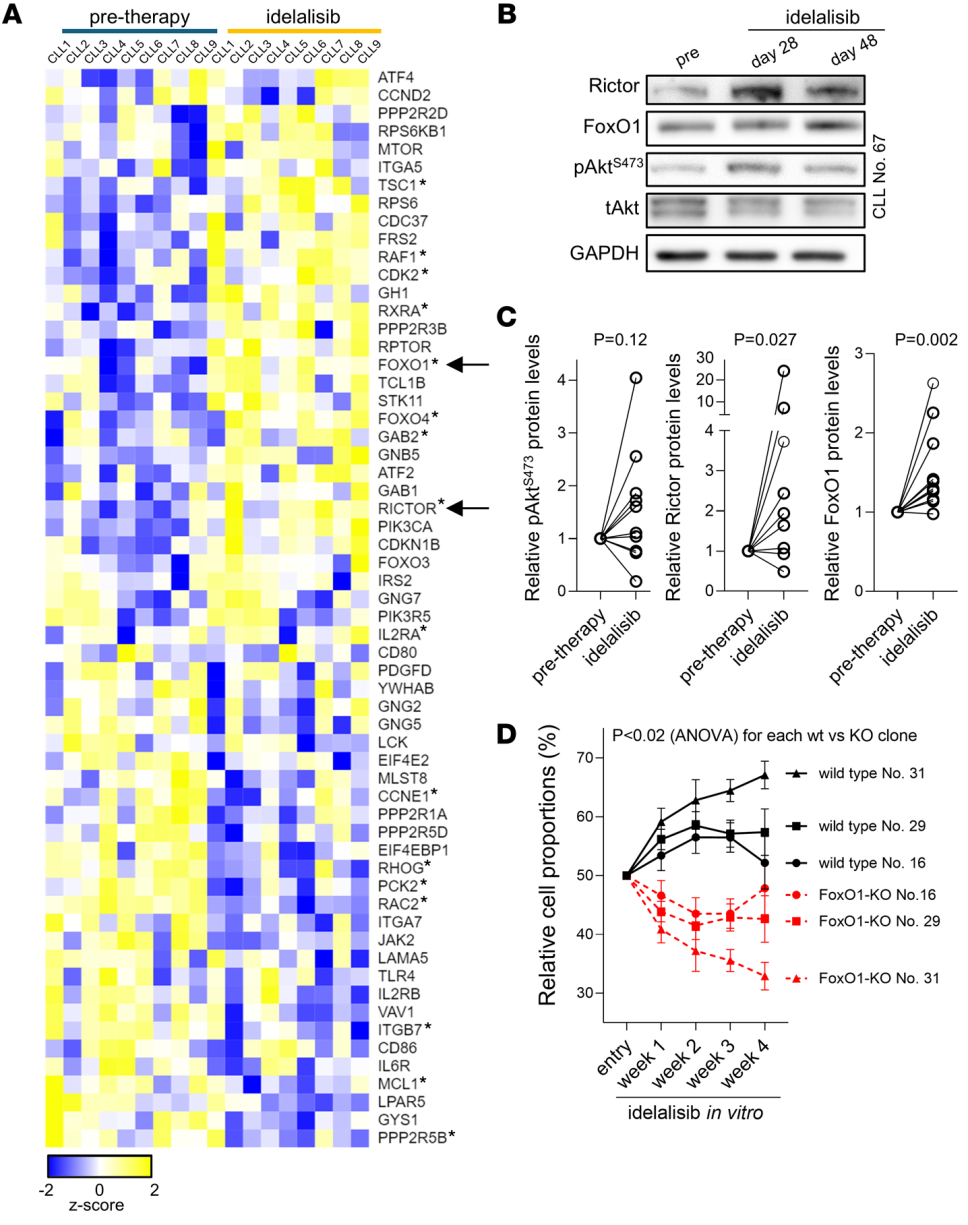
FoxO1/Rictor/pAkt^S473^ is responsible for adaptation to idelalisib. (**A**) Heatmap of differentially expressed genes from samples obtained before and during idelalisib treatment in vivo (*P* adj < 0.05; base mean > 100; *n* = 11 pairs) and overlapped with 2 databases of genes involved in PI3K/Akt signaling (gene sets: no. M27162, reactome_PI3K_AKT_signalling_in_cancer and has04151, KEGG: PI3K-Akt signaling pathway). Lower expression indicated in blue, higher in yellow. Genes marked with asterisks were also differentially expressed after ibrutinib treatment in vivo (see Figure 2A). List of top 500 differentially expressed genes is included in Supplemental Table 5. (**B**) Representative immunoblot of Rictor, FoxO1, and pAkt^S473^ in primary CLL sample treated with idelalisib in vivo. (**C**) Densitometric quantification of relative pAkt^S473^, Rictor, and FoxO1 protein levels analyzed by immunoblot in primary samples of CLL patients treated with idelalisib in vivo for 2–4 weeks (*n* = 11 for pAkt^S473^ and FoxO1; *n* = 9 for Rictor). *P* values were calculated using Wilcoxon’s test. For patient characteristics, see Supplemental Table 8. (**D**) Competitive growth of WT versus *FoxO1*-KO MEC1 cells in medium with idelalisib (2 μM, 4 weeks) relative to growth in medium with vehicle (DMSO). WT and *FoxO1*-KO cells marked with GFP or AZURIT (and vice versa) and mixed in 1:1 ratio (*n* = 4 repetitions for each of the 3 clones, WT clones are marked by numbers corresponding to the specific KO clone that was used in the corresponding competitive growth experiment). Graph represents the percentage of KO versus WT idelalisib-treated cells, and this is plotted relatively to vehicle-treated (DMSO) KO or WT cells, respectively, to correct for any effect of the KO on idelalisib-unrelated cell fitness. Statistical difference was tested using 2-way ANOVA with Geisser-Greenhouse correction.

To test the relevance of the FoxO1/pAkt^S473^ axis further, we investigated the effects of the PI3Kδ inhibitor idelalisib in this context. We performed RNA expression profiling of samples obtained from patients before and during single-agent idelalisib therapy (*n* = 9 patients, 18 samples; median time on therapy, 4 weeks; range, 2–5 weeks; Supplemental Tables 5 and 6). We profiled genes involved in the PI3K/Akt pathway and found a major overlap with the ibrutinib effect in vivo, with 16 genes being changed in the same manner during both ibrutinib and idelalisib therapy, including upregulated *FoxO1* and *Rictor* (Figure 5A and Supplemental Table 7). We also confirmed FoxO1 and Rictor protein level upregulation during idelalisib treatment (*P* = 0.002 and 0.027, respectively; Figure 5, B and C) and increase in pAkt^S473^ in approximately 64% of samples obtained during idelalisib treatment (7 out of 11 patients; Figure 5, B and C). This is in line with a recent publication describing Akt phosphorylation in response to PI3Kδ inhibition in some patients (42). Notably, *FoxO1*-KO MEC1 cells had decreased fitness when treated with idelalisib for 4 weeks compared with *FoxO1* WT cells (Figure 5D). Moreover, higher FoxO1 levels in primary CLL samples were also associated with increased resistance to ibrutinib or idelalisib in vitro (Supplemental Figure 8). Altogether, FoxO1 induces Rictor as an adaptation mechanism to BTK or PI3K inhibition that acts by Akt activation downstream of these BCR-associated kinases.

### FoxO1 inhibition induces apoptosis and potentiates BTK inhibitors’ effects.

**Figure 6:**
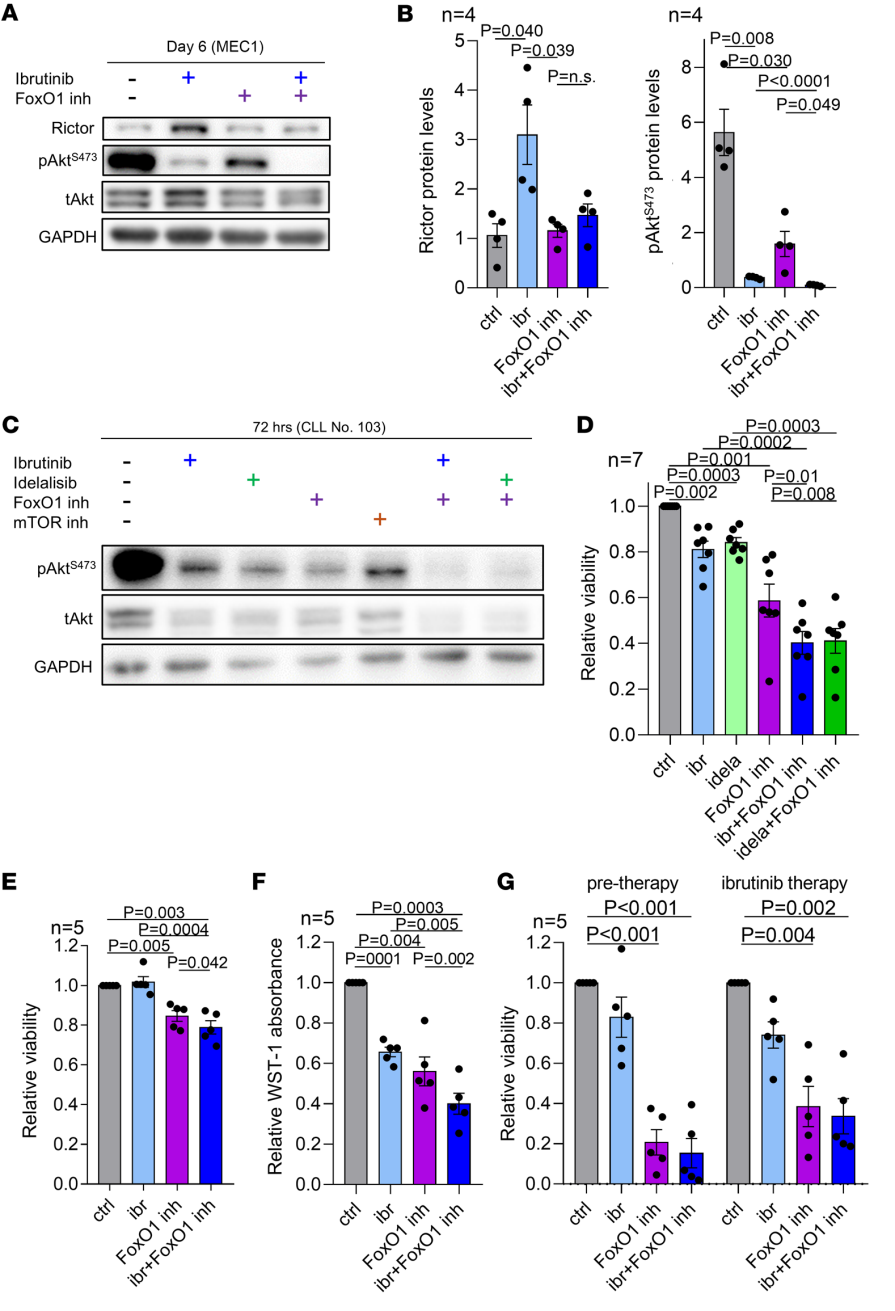
FoxO1 is a potential therapeutic target alone or in combination with BCR inhibitors. (**A**) Representative immunoblot of MEC1 cells treated with ibrutinib (2 μM), FoxO1 inhibitor (0.5 μM), or their combination for 6 days. (**B**) Densitometric quantification of Rictor and pAkt^S473^ protein levels in MEC1 cells treated with ibrutinib (2 μM), FoxO1 inhibitor (0.5 μM), or their combination for 6 days (*n* = 4). (**C**) Representative immunoblot of primary CLL cells pretreated with ibrutinib (1 μM) or idelalisib (1 μM) for 24 hours and subsequently with FoxO1 inhibitor (0.5 μM) or AZD8055 (mTOR inh, 0.5 μM) added to the culture for additional 48 hours. (**D**) Relative viability of primary CLL cells (*n* = 7) pretreated with vehicle, ibrutinib (1 μM), or idelalisib (1 μM) for 24 hours and then treated with FoxO1 inhibitor (0.5 μM) for additional 48 hours. Combination index = 1.01 for ibrutinib and FoxO1 inhibitor, and 0.97 for idelalisib and FoxO1 inhibitor. For patient characteristics, see Supplemental Table 8. (**E**) Relative viability of MEC1 cells treated with ibrutinib (2 μM), FoxO1 inhibitor (0.5 μM), or their combination (96 hours, *n* = 5). Combination index = 0.64. (**F**) Relative WST-1 absorbance in MEC1 cells treated (48 hours) with ibrutinib (2 μM), FoxO1 inhibitor (0.5 μM), or their combination (*n* = 5). (**G**) Relative viability of paired CLL samples (*n* = 5) obtained before and after 1 month (*n* = 2) or 2 months (*n* = 3) of ibrutinib therapy in vivo. Upon thawing, cells were treated with ibrutinib (1 μM), FoxO1 inhibitor (0.5 μM), or their combination (72 hours). For patient characteristics, see Supplemental Table 8. All *P* values in Figure 5 were calculated using paired *t* test.

We next tested the effects of the FoxO1 inhibitor on cell viability and CLL cell adaptation to ibrutinib. The combination of ibrutinib and FoxO1 inhibitor blocked ibrutinib-induced Rictor upregulation and pAkt^S473^ restoration in MEC1 cells (Figure 6, A and B). Similarly, Akt phosphorylation was nearly completely eliminated in primary CLL cells treated with a combination of FoxO1 inhibitor with ibrutinib or idelalisib (Figure 6C). Notably, a submicromolar concentration of FoxO1 inhibitor (0.5 μM) decreased primary CLL cells’ viability by approximately 40% (*n* = 7, *P* = 0.001; Figure 6D and Supplemental Figure 9A), and FoxO1 inhibition combined with ibrutinib or idelalisib was more potent in inducing primary CLL cells and MEC1 cell apoptosis in vitro (*P* < 0.05, mostly additive effect; Figure 6, D–F). Akt^S473^ phosphorylation and primary CLL cell viability was also reduced by the combination of FoxO1 inhibitor with acalabrutinib or pirtobrutinib (Supplemental Figure 9, B and C).

The high degree of spontaneous apoptosis did not allow us to study the adaptation of primary CLL cells to ibrutinib in vitro, since FoxO1/Rictor/pAkt^S473^ axis induction requires continuous ibrutinib exposure for several days. To overcome this, we took paired samples from CLL patients before and during ibrutinib treatment in vivo and exposed them to FoxO1 inhibitor in vitro (72 hours). This showed that the FoxO1 inhibitor is highly toxic to CLL cells obtained during ibrutinib therapy, but there was no increase in apoptosis compared with pretherapy samples (Figure 6G). We attribute this to the high toxicity of the FoxO1 inhibitor alone (cell killing ~70%), and it demonstrates that CLL cells exposed to ibrutinib are sensitive to FoxO1 inhibition. The FoxO1 inhibitor was also toxic for CLL cells obtained during acalabrutinib in vivo therapy (~55% killing, *P* = 0.026; Supplemental Figure 9D).

### FoxO1 inhibition overcomes microenvironmental protection and blocks proliferation induced by T cell factors.

**Figure 7:**
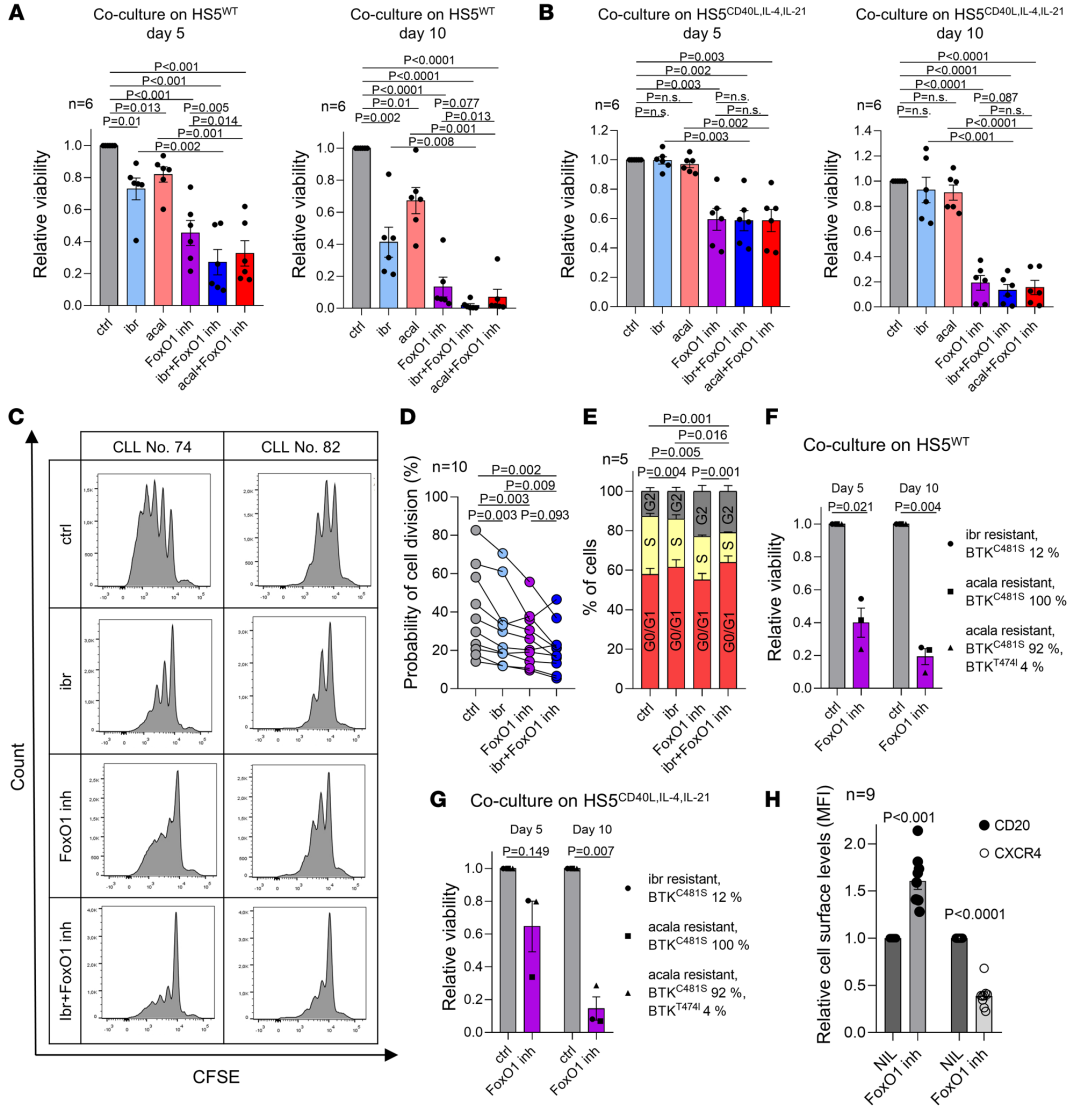
FoxO1 inhibition overcomes microenvironmental protection and blocks CLL cells’ proliferation induced by T cell factors. (**A** and **B**) Relative viability of primary CLL cells cocultured with HS5 cells. CLL cells were cocultured for 5 or 10 days on (**A**) WT HS5 or (**B**) HS5^CD40L,IL-4,IL-21^ and treated with ibrutinib (1 μM), acalabrutinib (acal, 1 μM), FoxO1 inhibitor (0.5 μM), or their combination. (**C** and **D**) Proliferation of primary CLL cells cocultured with stromal cells HS5^CD40L,IL-4,IL-21^ treated with ibrutinib (1 μM), FoxO1 inhibitor (0.5 μM), or their combination. (**C**) Representative CFSE staining histogram in 2 primary CLL samples. Proliferation rate quantified by dilution of CFSE signal. (**D**) Probability (calculated from precursor frequency) that cells will divide at least once (*n* = 10). (**E**) Cell cycle measured by propidium iodide (PI) staining in MEC1 cells treated with ibrutinib (1 μM), FoxO1 inhibitor (0.5 μM), or their combination for 96 hours (*n* = 5). *P* values are calculated for differences in percentages of cells in S phase. (**F** and **G**) Relative viability of primary CLL cells obtained for patients at the time of progression on BTK inhibitors and cocultured for 5 or 10 days on (**F**) WT HS5 or (**G**) HS5^CD40L,IL-4,IL-21^ and treated with FoxO1 inhibitor (0.5 μM). (**H**) Relative levels of cell-surface CD20 and CXCR4 levels in primary CLL cells (*n* = 9) treated with FoxO1 inhibitor (0.5 μM, 48 hours). For patient characteristics, see Supplemental Table 8. All *P* values in Figure 6 were calculated using paired *t* test.

The FoxO1 inhibitor had a strong effect on CLL cell viability in vitro (see above), and we further tested its effects in coculture models, which provide resistance to cytostatic drugs, venetoclax, or monoclonal antibodies (43–46). FoxO1 inhibitor induced significant apoptosis (85%-90% at 10 days; *P* < 0.0001) in primary CLL cells cocultured with HS5 stromal cells or with HS5 stromal cells engineered to produce T cell factors CD40L, IL-4, and IL-21 (Figure 7, A and B) (47). The effect of the combination of the FoxO1 inhibitor with ibrutinib or acalabrutinib was mostly additive in HS5^WT^ cocultures (day 5; Figure 7A). The FoxO1 inhibitor was also able to reduce the proliferation of CLL cells cocultured with HS5^CD40L,IL-4,IL-21^, and this was further decreased when combined with ibrutinib (Figure 7, C and D). The FoxO1 inhibitor also significantly reduced the percentage of MEC1 cells in the S phase (Figure 7E and Supplemental Figure 10A). Interestingly, ibrutinib alone also slightly inhibited proliferation, but acalabrutinib did not (Supplemental Figure 10B), suggesting the role of ibrutinib off targets in this phenomena. To study the FoxO1/Rictor/pAkt^S473^ axis in the coculture setting, we cocultured MEC1 with HS5^WT^ in the presence or absence of ibrutinib for several days. This showed that, similarly to MEC1 cultures on plastic, the ibrutinib treatment increases Rictor levels and leads to some pAkt^S473^ recovery (Supplemental Figure 11A). However, this phenotype is less pronounced, as the HS5^WT^ coculture itself leads to changes in FoxO1 levels (degradation) due to microenvironmental stimuli. Together the data show that ibrutinib is able to also initially repress the pAkt^S473^ in the context of CLL cell interaction with HS5^WT^ cells and that over time pAkt^S473^ is partially restored. Notably, the FoxO1 inhibitor was toxic (> 70% cell killing) for BTK inhibitor–resistant primary CLL cells with BTK mutations cultured on plastic or in the coculture setting (*P* < 0.05; Figure 7, F and G, and Supplemental Figure 11B). Importantly, in a cell line–derived xenograft mouse model, the bone marrow infiltration of immunodeficient mice by MEC1 cells was reduced by approximately 2.6-fold when ibrutinib was combined with the FoxO1 inhibitor as compared with treatment by ibrutinib alone (36% versus 14% marrow infiltration, *P* = 0.027; Supplemental Figure 12).

The therapeutic potential of FoxO1 inhibition might be increased by the observation that it upregulated CD20 levels (fold-change, 1.6, *P* < 0.001, *n* = 9; Figure 7H), which is in line with FoxO1 being a *CD20* repressor (48–50). It has been shown that CD20 levels are repressed by ibrutinib and idelalisib therapy as single agents (7, 51, 52), suggesting a potential synergism of FoxO1 inhibition with BCR inhibitors and anti-CD20 antibodies or anti-CD20 antibodies alone. Furthermore, the FoxO1 inhibitor reduces cell-surface CXCR4 and thus potentially impairs CLL cell migration (fold-change, 0.4, *P* < 0.0001, *n* = 9; Figure 7H). Together, the data suggest that FoxO1 inhibition disrupts microenvironmental signaling and is a promising therapeutic strategy alone or in combination with BCR inhibitors.

## Discussion

Here we show that induction of transcription factor FoxO1 during ibrutinib therapy upregulates Rictor, an mTORC2 assembly protein, leading to phosphorylation of Akt, an essential molecule supporting CLL cell survival. This adaptive increase in pAkt^S473^ levels occurred in approximately 70% of CLL cases during ibrutinib therapy and was associated with a more prominent ibrutinib-induced lymphocytosis. FoxO1 inhibition decreases CLL cell viability alone and more potently with BCR inhibition and blocks CLL proliferation induced by T cell signals (a combination of CD40L, IL-4 and IL-21).

We hypothesized that CLL cells might use nongenetic adaptation to survive BTK kinase inhibition. It has been shown that PI3K/Akt pathway modulation appears to be the key element controlling mature B cell survival, and constitutively active PI3K rescues BCR-deficient mature B cells from apoptosis (27, 28). Indeed, we observed that 70% of CLL patients on ibrutinib therapy (2–12 weeks) and 50% of patients on acalabrutinib (4–12 weeks) therapy had induced pAkt^S473^ levels, and pAkt^S473^ level recovery was also observed in MEC1 cells treated with BTK inhibitors in vitro. This is partially conterintuitive since the acute effect of ibrutinib is a reduction in pAkt^S473^ levels (see Figure 1C and Supplemental Figure 13) and suggests that CLL cells utilize pAkt^S473^ to survive the BTK/BCR signaling inhibition. This is in line with data from DLBCL cell lines where constitutively active Akt rescued BCR KO in vitro (53). Indeed, we observed that patients with upregulated pAkt^S473^ levels during ibrutinib therapy experienced higher and longer-lasting lymphocytosis after the start of treatment (Figure 1E). CLL cells obtained during ibrutinib therapy are also highly sensitive to Akt inhibition, with an increase in the apoptosis rate compared with pretherapy samples. This suggests a direct role for Akt in the survival of CLL cells during ibrutinib therapy and within the relatively stimuli-poor microenvironment of peripheral blood. The essential role of Akt in CLL cell survival has been shown previously, including its inhibition causing cell apoptosis and overactivation triggering Richter transformation (29–33, 54). BCR inhibitors induce mild cell apoptosis and have substantial cytostatic effects shown by interfering with CLL cell entrance to lymph nodes to obtain proproliferative CLL–T cell interactions (12, 37). pAkt^S473^ supports CLL cell survival, but it is not sufficient to allow for CLL cell proliferation and thus provides a survival adaptation, but not a complete resistance to BTK inhibition. Unfortunately, the short follow-up of the analyzed patient samples (and a small number of samples without pAkt^S473^ induction) did not allow us to study the association of pAkt^S473^ levels with Richter transformation or time to relapse. Notably, CLL cells obtained from BTK inhibitor–resistant patients with *BTK* mutations harbor phosphorylated Akt (Supplemental Figure 3) (55, 56). It is plausible that the early adaptation via pAkt^S473^ might support later development of fully resistant clones carrying mutations in *BTK* and/or *PLCG2* and/or other genes or might combine with the occurrence of such mutations later during therapy. It remains unclear whether *BTK* or *PLCG2* mutations are fully responsible for the survival of the whole CLL cell clone on therapy/during relapse since they are often present in a small CLL cell subpopulation (<5% variant allele frequency [VAF]) (8, 9), and 20%–35% of relapsing CLL patients do not carry mutations explaining the resistance (57–59).

Our RNA profiling of paired CLL samples obtained before and during ibrutinib therapy in vivo revealed upregulation of *Rictor*, an indispensable protein for mTORC2 protein complex assembly and function that directly phosphorylates Akt at S473 (34). *Rictor* KO in MEC1 cells confirmed its critical role for adaptive Akt^S473^ phosphorylation during ibrutinib exposure. To understand the mechanisms leading to Rictor upregulation, we analyzed the expression of transcription factors during ibrutinib therapy in CLL in vivo. This revealed that transcription factor FoxO1 directly binds in the *Rictor* promoter, and its activity determines *Rictor* levels in CLL cells during ibrutinib therapy. Notably, the FoxO1 targets identified by CUT&RUN in ibrutinib-treated MEC1 cells included 7 out of 25 PI3K/Akt pathway regulators changed during ibrutinib therapy, suggesting that FoxO1 plays a complex role in the PI3K pathway changes. Importantly, the *FoxO1*-KO cells had a significant disadvantage in competitive growth assay with ibrutinib when compared with *FoxO1* WT MEC1 cells (*P* < 0.0001), and *FoxO1*-KO MEC1 cells were also outgrown by the WT cells in immunodeficient mice. *FoxO1* KO or its chemical inhibition led to a dramatic downregulation of basal Akt^S473^ phosphorylation and also blocked pAkt^S473^ induction after BCR crosslinking. This demonstrates that FoxO1 is directly responsible for the *Rictor* activation and mTORC2’s subsequent ability to phosphorylate Akt at S473. This is in line with studies of mutated FoxO1 in DLBCL, where FoxO1 mutations causing its increased activity in the nucleus lead to increased Akt phosphorylation (60). Roberto et al. (60) suggested that FoxO1 regulates the transcription of *PHLPP1* phosphatase responsible for Akt dephosphorylation. However, we did not observe *PHLPP1* changes after ibrutinib/idelalisib therapy in CLL, and its promoter was not bound by FoxO1 (data not shown). We have previously shown that FoxO1 can transcriptionally induce the adaptor protein GAB1, an adaptor molecule for PI3K, and thus also contributes to PI3K/Akt axis activity via another mechanism (23, 61). Therefore, the increased Akt^S473^ phosphorylation after FoxO1 induction in CLL cells might be caused by the cooperation of 2 mechanisms, i.e., (a) Rictor upregulation leading to direct increase of mTORC2 kinase activity responsible for Akt^S473^ phosphorylation and (b) induced GAB1 levels leading to PI3K signaling amplification (23). Our data from experiments with idelalisib suggest that the former mechanism is of larger importance since PI3K kinase activity is not essential for the FoxO1/Rictor/pAkt^S473^ axis. The FoxO1/Rictor/pAkt^S473^ axis bypasses both BTK and PI3K, but the GAB1/PI3K axis requires functional PI3K. However, our data do not exclude the possibility that other mechanisms may (co)exist that support “tonic” Akt activity, such as the induction of surface BCR during ibrutinib therapy described by us previously (24). Notably, induction of FoxO1 and Rictor also occurs upon treatment with highly selective BTK inhibitors, namely acalabrutinib and pirtobrutinib. However, the initial inhibition followed by recovery of pAkt^S473^ levels in vitro is more pronounced with ibrutinib, likely due to its off-target activity on other BCR-related kinases (40, 41, 62). We also observed a decrease in pAkt^T308^ in *FoxO1-KO* MEC1, likely due to impaired PI3K recruited to the plasma membrane caused by lower GAB1 levels (23) and decreased recruitment of Akt to the plasma membrane (dependent on binding to PIP3 produced by PI3K). Moreover, Akt’s phosphorylation at S473 facilitates Akt’s phosphorylation at T308 by PDK1 (34, 63, 64). Notably, an increase in FoxO1/Rictor/pAkt^S473^ axis activity during BTK inhibitor treatment might also result in an increased propensity for signaling after BCR ligation with antigen once the BTK inhibitor is discontinued due to reasons other than the acquisition of *BTK/PLCG2* mutations (such as surgical intervention or toxicity) (65–67), since the same axis is involved in the amplification of the BCR signal (see Figure 2, I–K, and Figure 4, F–H).

We show that the FoxO1/Rictor/pAkt^S473^ axis is induced during ibrutinib treatment, and this helps CLL cells adapt and survive in peripheral blood, which associates with the extent of lymphocytosis following the treatment initiation. This may be a “preprogrammed” mechanism for CLL cells’ survival in stimuli-poor peripheral blood, as FoxO1 and Rictor are upregulated in intraclonal CXCR4^bright^CD5^dim^ subpopulations (Supplemental Figure 14A), the CLL cell subpopulation circulating in peripheral blood for an extended time (7, 23, 68). Moreover, BTK inhibition in vivo causes changes in intraclonal CXCR4/CD5 composition, and CXCR4^bright^CD5^dim^ cells become a dominant subpopulation (Supplemental Figure 14B). FoxO1 might also be involved in the transcriptional regulation of other proteins contributing to adaptation or resistance to BCR inhibition, such as integrin signaling (17, 69) or CXCR4 induction (70), and this likely depends on interactions with other transcription factors as evidenced in MCL (71). For example, it has been shown that FoxO1 induces IGF1R, and this contributes to idelalisib resistance in TCL1-transgenic mice (72); however, we did not observe *IGF1R* induction during several weeks of idelalisib or ibrutinib therapy in CLL in vivo. Activating the FoxO1/Rictor/pAkt^S473^ pathway is not exclusive to ibrutinib treatment, as the axis is also induced during acalabrutinib or idelalisib treatment. Moreover, *FoxO1*-KO cells were significantly less able to proliferate during competitive culture experiments with either idelalisib or ibrutinib (*P* < 0.02 and *P* < 0.0001, respectively). It has been previously shown that strong BCR activation downregulates FoxO1 via its degradation via the PI3K axis, but also on a transcriptional level, which could be blocked by PI3K inhibition or independently via the BTK/PLCγ2/Ca^2+^ axis (73). It is likely that the inhibition of BCR signaling by BTK or PI3K inhibition leads to upregulation of *FoxO1* mRNA in CLL cells because its transcription is not repressed by the “continuous” tonic BCR signaling. FoxO1 can also be regulated at the protein level by Akt, which phosphorylates FoxO1, leading to subsequent ubiquitination and degradation. Acute exposure of CLL cells to ibrutinib leads to a nearly complete loss of pAkt^S473^ and stabilization of FoxO1 protein levels, which triggers transcription of FoxO1 targets such as Rictor, but possibly also FoxO1 itself (FoxO1 binds its own promotor; ref. 74). This likely explains why we observed FoxO1 changes on both protein and mRNA levels during BTK/PI3K inhibitor therapy.

It has been suggested that FoxO1 and Akt activity are mutually exclusive since Akt activation leads to FoxO1 phosphorylation, its sequestration from the nucleus, and degradation (75, 76). However, we and others had previously shown that active Akt and nuclear FoxO1 are not mutually exclusive in malignant B cells (23, 77) and observed FoxO1 nuclear accumulation in CLL cells obtained during ibrutinib therapy (23). Despite FoxO1’s reported role as a tumor suppressor, recent reports linked high FoxO1 expression to poor prognosis in B cell malignancies (77, 78). Approximately 10% of DLBCL and Burkitt lymphomas carry FoxO1 mutations that lead to FoxO1 retention in the nucleus (specifically T24 mutation) and its increased transcriptional activity (77, 79, 80). Here, we show that FoxO1 inhibition represents a potential therapeutic target in CLL since it decreases CLL cells’ viability, and this effect is more profound when combined with ibrutinib, acalabrutinib, pirtobrutinib, or idelalisib. The FoxO1 inhibitor is also toxic for CLL cells resistant to BTK inhibitors due to *BTK* mutations underscoring its therapeutic potential. Furthermore, FoxO1 inhibition reduced the viability of CLL cells cocultured with stromal cells and CLL cell proliferation induced by the T cell factors (CD40L, IL-4, and IL-21), and this was potentiated in combination with ibrutinib. The cytotoxic and cytostatic effects of FoxO1 inhibitor also translated into reduced MEC1 cell growth in immunodeficient mice treated by a combination of the FoxO1 inhibitor with ibrutinib as compared with ibrutinib alone. The antiproliferative effects of the FoxO1 inhibitor might be related not only to Akt inhibition but also to the important role of FoxO1 in cell metabolism (81) and possibly some off-target activity against FoxO3a and FoxO4 (82). Interestingly, ibrutinib impaired CLL cell proliferation in the HS5^CD40L,IL-4,IL-21^ cocultures, but acalabrutinib did not, and this is likely due to the off-target activity of ibrutinib against JAK3 (IC_50_: 240 nM), which is required for interleukin signaling (40). The effect of FoxO1 inhibition on cell viability has been recently shown in MCL (71). FoxO1 inhibitors are being developed for clinical testing in type 2 diabetes and cardiovascular diseases (82–84). Several teams, including us, have treated small animals with the FoxO1 inhibitor AS1842856 and reported good tolerance of the inhibitor (82, 85).

**Figure 8:**
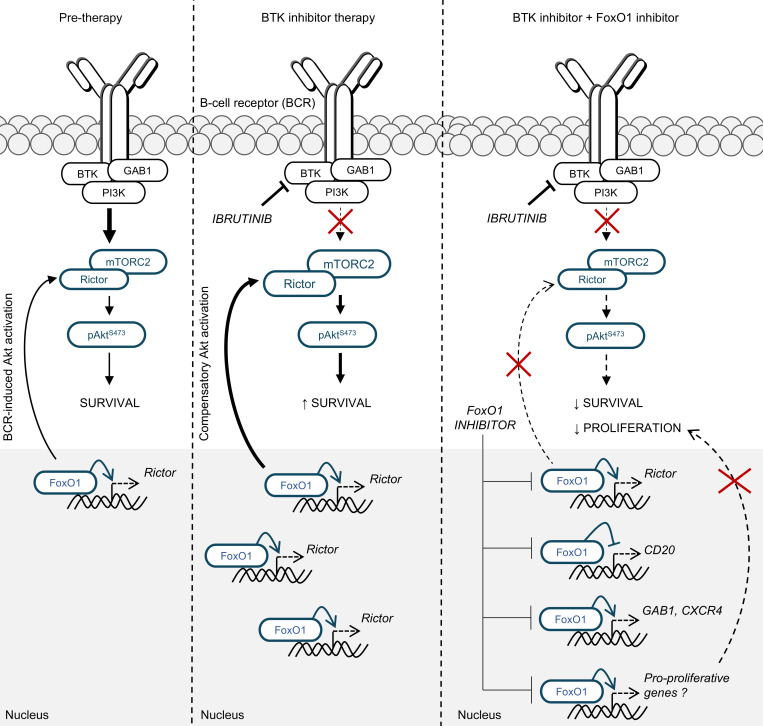
Role of FoxO1/Rictor/pAkt^S473^ axis in the adaptation to BTK inhibition. BTK inhibitor treatment leads to FoxO1 induction. FoxO1 subsequently binds to the *Rictor* promoter and increases its expression, which supports mTORC2 complex activity. mTORC2 directly phosphorylates Akt at S473 independently of BCR-associated kinases BTK and PI3K. This adaptation mechanism to BTK inhibitor treatment can be overcome by FoxO1 inhibition, which leads to apoptosis of cells and inhibition of proliferation.

In summary, we have described the FoxO1/Rictor axis as being involved in a BTK and PI3Kδ-independent mechanism inducing pAkt^S473^ activity in CLL cells during BCR inhibitor therapy. This is associated with CLL cell survival and a more prominent lymphocytosis on therapy (summarized in Figure 8). This represents early nongenetic adaptation mechanism to BTK inhibition in CLL and adds to other previously described mechanisms in B cell lymphomas. We have also shown that FoxO1 is a potential therapeutic target in CLL.

## Methods

### Sex as a biological variable.

In experiments with primary CLL cells, the sex was not considered as a biological variable and samples from both sexes were used corresponding to the natural incidence in the population. For competitive growth assay of MEC1 cells in mice, both sexes of mice were used and the results were consistent irrespectively of the use of male versus female mice. For in vivo testing of ibrutinib/FoxO1 inhibitor treatment, only female mice were selected to obtain maximal consistency in engraftment based on our previous experience, but the results should not be influenced by use of one animal sex.

### CLL samples and cell lines.

Purified CLL samples contained 95% or more of CD5^+^19^+^ cells (see Supplemental Methods). MEC1 and HS5 cells were obtained from the DSMZ and ATCC, respectively.

### Gene expression.

For RNA-Seq analysis, libraries were prepared as described previously (23, 37) using the TruSeq Stranded messenger mRNA (mRNA) LT Sample Prep Kit (Illumina) and sequenced with the NextSeq 500/550 High Output, version 2.5, Kit (Supplemental Methods).

### Cell treatments, competitive assay, and coculture.

MEC1 cells (1 × 10^6^/ml) and CLL cells (10 × 10^6^/ml) were treated with ibrutinib (2 μM for MEC1, 1 μM for primary CLL cells), acalabrutinib (5 μM for MEC1, 1 μM for primary CLL cells), pirtobrutinib (2 μM for MEC1, 1 μM for primary CLL cells), idelalisib (2 μM for MEC1, 1 μM for primary CLL cells), MK-2206 (Akt inhibitor, 1.25, 2.5, 5 and 10 μM), AS1842856 (FoxO1 inhibitor, 0.5 μM), or AZD8055 (mTOR inhibitor, 0.5 μM; all Selleckchem, pirtobrutinib, MedChemExpress). Cells were stimulated with soluble or bead-bound (Dynabeads M-270-Epoxy; Thermo Fisher Scientific) goat F(ab′)2 anti-human IgM (anti-IgM, Southern Biotechnology) (23, 37, 61) (see Supplemental Methods). For competitive growth assay, MEC1^WT^ and MEC1^FoxO1-KO^ or MEC1^Rictor-KO^ clones were traced with a plasmid encoding GFP or AZURIT, mixed in a 1:1 ratio (wt:KO) and treated with DMSO or ibrutinib/idelalisib. For coculture experiments, CLL cells loaded with CFSE were seeded onto γ-irradiated HS5^WT^ or HS5^CD40L,IL-4,IL-21^ cells (20:1 ratio) (47) and treated with DMSO or inhibitors (5/10 days). For details, see Supplemental Methods.

### Genome-wide binding of FoxO1 by CUT&RUN.

CUT&RUN was performed according to the protocol using protein A/G–Micrococcal Nuclease (pA/G-MNase) fusion protein (Addgene ID:123461) and Triton X-100–based nuclear extraction (86) (see Supplemental Methods).

### Cell line–derived xenograft mouse models.

For competitive in vivo growth assay, MEC1^WT^ and MEC1^FoxO1-KO^ clones were traced with a plasmid encoding GFP or AZURIT and were mixed in a 1:1 ratio and injected into the tail vein of NSG (NOD.Cg-*Prkdc^scid^* *Il2rg^tm1Wjl^*/SzJ, The Jackson Laboratory) mice. Mice were sacrificed 3 weeks after the MEC1 injection, and spleen, liver, blood, and bone marrow were analyzed for the ratio of GFP^+^/AZURIT^+^ cells by flow cytometry. For details, see Supplemental Methods.

For in vivo testing of ibrutinib/FoxO1 inhibitor treatment, GFP-positive MEC1 cells (3 × 10^6^) were intravenously injected into female NRG mice (NOD.Cg-*Rag1^tm1Mom^* *Il2rg^tm1Wjl^*/SzJ, The Jackson Laboratory). Mice were divided randomly after 3 days of leukemia engraftment into 2 groups and treated with either ibrutinib or a combination of ibrutinib and FoxO1 inhibitor. Mice were sacrificed 14 days after the MEC1 injection, and bone marrow was analyzed to determine the percentage of GFP^+^ cells. For details, see Supplemental Methods.

### Statistics.

Apart from NGS analysis (Supplemental Methods), all statistical analyses were performed with Prism, version 8.0.1 (GraphPad). Data in graphs represent mean ± SEM.

### Study approval.

The institutional review board (Ethics Committee of University Hospital, Brno, Czechia) approved the study, and samples were obtained with written, informed consent according to the Declaration of Helsinki.

### Data availability.

The RNA-Seq analysis results may be found in a data supplement available with the online version of this article. Data have been deposited at the European Genome-phenome Archive (EGA), which is hosted by the European Bioinformatics Institute (EBI) and the Centre for Genomic Regulation (CRG) (EGAS50000000620 and EGAS50000000621). Values for all data points in graphs are reported in the Supporting Data Values file. For other original data or detailed protocols, contact the corresponding author.

## Author contributions

LO designed the study, performed experiments, analyzed the data, and wrote the article. VS, KH, PP, EH, GC, LK, and PFZ performed experiments. GMP performed the NGS experiments. DF analyzed the data. JV, ZK, RV, and KS performed in vivo experiments with immunodeficient mice. JO analyzed the NGS data. VS, AMD, SC and JIMS performed the CUT&RUN experiment and analyzed the data. AP, KP, SP, MS, FV, DL, SMF, MSD, JRB, MD, FF, and JM provided samples and clinical data. MM designed the study, interpreted the data, and wrote the article. All authors edited and approved the article for submission.

## Supplementary Material

Supplemental data

Unedited blot and gel images

Supporting data values

## Version 1 10/22/2024

In-Press Preview

## Version 2 12/02/2024

Electronic publication
