# Supplementary material for: FoxO1/Rictor axis induces a nongenetic adaptation to ibrutinib via Akt activation in chronic lymphocytic leukemia
Source: J Clin Invest. 2024 Oct 22;134(23):e173770. doi: 10.1172/JCI173770 (PMC11601945; doi:10.1172/JCI173770)
Supplement: Supplemental data [file jci-134-173770-s148.pdf]

Supplemental Materials

Ondrisova et al.

Correspondence:

Marek Mraz, M.D., Ph.D.
Associate Professor of Oncology
Central European Institute of Technology, Masaryk University
Kamenice 5, 625 00 Brno, Czech Republic
E-mail: marek.mraz@email.cz
Tel.: +420 549498143

1) Supplemental Methods

2) Supplemental Figures: 14

3) Supplemental Tables: 8

1) Supplemental Methods

CLL samples and cell lines

Primary CLL samples were collected with written informed consent according to the Declaration of Helsinki, and the Institutional Review Board approved the study. Peripheral blood samples were obtained from patients who did not receive any therapy for at least six months (except the ibrutinib/idelalisib treated patients). The samples from ibrutinib/acalabrutinib/idelalisib-treated patients were collected prior to the drug administration and during therapy at the time points indicated in the figure legend and supplemental Table 8. Primary CLL samples were purified by negative selection with RosetteSep Human B Cell Enrichment Cocktail (Stemcell Technologies) and RosetteSep Human CD3 Depletion Cocktail (Stemcell Technologies) and Ficoll-Paque PLUS (Sigma Aldrich) according to manufacturer's protocol. In some cases of ibrutinib or idelalisib treated patients, PBMCs were isolated by density gradient centrifugation (CLL 1-12) as described previously (1) or isolated and subsequently purified by magnetic separation using anti-CD3 MicroBeads (Miltenyi Biotec) (CLL 19-23; 60-65; 110-112) as described previously (2). Final purity was ≥95% of CD5+19+ cells in all cases (evaluated by flow cytometry). Purified primary CLL cells were cultivated in RPMI-1640 (Sigma-Aldrich) supplemented with 20% fetal bovine serum (Biosera) and 100 $U \cdot ml^{-1}$ / 100 $\mu g \cdot ml^{-1}$ of penicillin/streptomycin (Sigma Aldrich) and in 5% CO_2 at 37 °C. *BTK* and *PLCG2* mutations in BTK inhibitor-resistant patients were detected as described previously (3).

Cell culture

MEC1 cell line was obtained from the German Collection of Microorganisms and Cell Cultures (DSMZ) and was cultivated in Iscove's Modified Dulbecco's Medium (IMDM, Biosera) supplemented with 10% fetal bovine serum (Biosera) and 100 $U \cdot ml^{-1}$ / 100 $\mu g \cdot ml^{-1}$ penicillin/streptomycin (Sigma Aldrich) and further cultivated in 5% CO_2 at 37ºC. HS5 and HEK293T cell lines were obtained from American Type Culture Collection (ATCC) and both were cultured in Dulbecco's Modified Eagle's Medium (DMEM, Biosera) supplemented with 10% fetal bovine serum (Biosera) and 100 $U \cdot ml^{-1}$ / 100 $\mu g \cdot ml^{-1}$ penicillin/streptomycin (Sigma Aldrich) in 5% CO_2 at 37ºC. For co-culturing CLL cells, $HS5^{wild\text{-}type}$ or $HS5^{CD40L,IL\text{-}4,IL\text{-}21}$ cells were γ-irradiated (20 Gy), seeded 5×10^4 cells / cm^2 into 0.1% gelatin coated plates or dishes (TPP) and kept at 37 °C with 5 % CO_2 in DMEM media for 24 hrs. On the following day CLL cells were added in a 20:1 ratio (CLL:HS5) in suitable amount of RPMI media containing RPMI-1640 (Biosera) supplemented with 20% fetal bovine serum (Biosera), 100 U·ml−1/100 $\mu g \cdot ml^{-1}$ penicillin/streptomycin (Sigma Aldrich), 2 mM L-Glutamine (Biosera) and 70 nM 2-β-mercaptoethanol (Sigma Aldrich).

Plasmid constructs and genome editing

The gRNA sequences targeting the *Rictor* and *FoxO1* gene were designed in Benchling (Biology Software, 2019) online tool. The sequence 5'-CACCGTTACCTCGGGTCAGATCCAG-3´ for Rictor-gRNA or 5'-CACCGGGGCTGTGCGGGGACTTCCA-3' for FoxO1-gRNA was cloned into pSpCas9(BB)-2A-GFP vector (#48138, Addgene). To obtain *Rictor* or *FoxO1* knock-outs, the MEC1 wild-type cells were electroporated (Neon Transfection System, Thermo Fisher Scientific; 1500 V / 20 ms / 2 pulses) with prepared plasmids. After 48 hrs viable cells were sorted for GFP-positivity, expanded, sorted for single cells, expanded and individual clones were validated for absence of Rictor or FoxO1 on protein level.

To prepare $HS5^{CD40L,IL\text{-}4,IL\text{-}21}$, cells were transduced with constructs containing the i) coding sequence (CDS) for human IL-4 and puromycin cloned into the pAIP lentiviral vector (Addgene plasmid #74169), ii) CDS for human CD40L and blasticidin resistance cloned into the pEZ-Lv197 lentiviral vector (Genecopoeia/THP medical products, #EX-G0117-Lv197), and iii) CDS for human IL-21 cloned into pBMN-IRES-LyT2 retroviral vector (gift from Dr. D. Hodson, University of Cambridge, Cambridge, United Kingdom). Constructs were propagated in Stbl3 E. coli (Invitrogen). For transducing HS5 cells, viral packaging plasmids δR8.91 or gag/pol (lentiviral or retroviral), VSV-G and L-GFP (gift from Dr. M. Smida, CEITEC MU, Brno, Czech Republic) were used as follows: 3538 ng δR8.91 or gag/pol, 462 ng VSV-G (envelope), 400 ng L-GFP (transfection efficacy) and 2 µg of a gene-of-interest construct was used to transfect each 6 cm^2 dish of HEK-293T cells, after mixing with 50 µl of Opti-MEM media (Gibco), 7 µl of DharmaFect Duo (Dharmacon) and 2 ml DMEM (Biosera). After 24 hrs cells were washed with fresh DMEM media. The virus supernatant was collected after 24, 36, 48 and 60 hrs and the media filtered through a 0.45 µM filter and 10 µg/ml of polybrene (Sigma Aldrich) was

directly put onto HS5 cells seeded in two 6 cm^2 Petri dishes (TPP). The media was replaced the next day and the selection with 10 µg/ml puromycin (Sigma Aldrich) or 10 µg/ml blasticidin (Gibco) was started 1-2 days later and lasted for up to one week. Cells were maintained at 37 °C with 5 % CO_2 in DMEM media with 10 % fetal bovine serum without antibiotics.

For in vitro growth assay MEC1$^{wild\text{-}type}$ and clones of MEC1$^{FoxO1\text{-}KO}$ cell lines were transduced by GFP or AZURIT encoding vectors (both obtained from Addgene; #36083 and #36086, respectively) as described above. Cells were subsequently sorted for GFP or AZURIT positive cells.

For transient FoxO1 overexpression, MEC1 *FoxO1* cells were electroporated (Neon Transfection System, Thermo Fisher Scientific; 1500 V / 20 ms / 2 pulses) with pcDNA3 Flag FoxO1 constitutively active AAA mutant plasmid (Addgene, #13508) or pcDNA3.1-FLAG (Addgene, #210342) and cells were collected 48 hours after electroporation.

To prepare MEC1 *FoxO1*-KO cells with doxycycline-inducible FoxO1 overexpression, *FoxO1* gene from pcDNA3 Flag FoxO1 plasmid (Addgene, #13507) was amplified using 5'-TGGAGAATTGGCTAGCATGGCCGAAGCGCCTCAGGTGGTGGAGATCGACCCG-3´ and 5'-CCCCAACCCCGGATCCTCAGCCTGACACCCAGCTATG-3´ primers and Q5® Hot Start High-Fidelity 2X Master Mix (New England Biolabs) and cloned into pCW57-MCS1-P2A-MCS2 plasmid (Addgene, #80921). Empty pCW57-MCS1-P2A-MCS2 plasmid was used as control. MEC1 cells were then transduced by the plasmid in the same way as HS5$^{CD40L,IL\text{-}4,IL\text{-}21}$ as described above.

Competitive growth assay, cell treatments and stimulations

MEC1$^{wild\text{-}type}$ and MEC1$^{FoxO1\text{-}KO}$ clones were traced with a plasmid encoding GFP or AZURIT, respectively (and *vice versa* to eliminate potential bias from constructs), and their equal numbers were mixed in a 1:1 ratio (validated by flow cytometry) and seeded with DMSO or ibrutinib or idelalisib (both 2 µM, Selleckchem). For MEC1$^{wild\text{-}type}$ and MEC1$^{Rictor\text{-}KO}$ clones competitive growth assay, MEC1$^{wild\text{-}type}$ traced with a plasmid encoding GFP or AZURIT and unstained MEC1$^{Rictor\text{-}KO}$ clones were mixed in a 1:1 ratio and seeded with DMSO or ibrutinib (2 µM, Selleckchem). Fresh media with DMSO or ibrutinib was added to the cells three times a week and changes in ratios (KO vs wt) were measured by flow cytometry after 1 to 4 weeks of the treatment.

For in vitro treatments, MEC1 cells (1×10^6/ml) and primary CLL cells (10×10^6/ml) were treated with ibrutinib (2 µM for MEC1, 1 µM for primary CLL cells), acalabrutinib (5 µM for MEC1, 1 µM for primary CLL cells), pirtobrutinib (2 µM for MEC1, 1 µM for primary CLL cells), idelalisib (2 µM for MEC1, 1 µM for primary CLL cells), MK-2206 (Akt inhibitor, 1.25, 2.5, 5 and 10 µM), AS1842856 (FoxO1 inhibitor, 0.5 µM), AZD8055 (mTOR inhibitor, 0.5 µM; all Selleckchem, pirtobrutinib MedChemExpress).

For BCR activation by bead-bound anti-IgM, Dynabeads M-270 Epoxy were coated according to Dynabeads Antibody Coupling Kit (Thermo Fisher Scientific) with goat F(ab')2 anti-human IgM (4, 5) (10 µg of antibody/mg beads) at 37°C overnight and then resuspended in the recommended buffer at

a final bead concentration of 10 mg of antibody coupled beads/ml. Primary CLL cells (10×10^6/ml) were incubated with bead-bound anti-IgM or the isotype control in complete RPMI-1640 medium for 3 hrs at 37°C (at a 2:1 beads to cell ratio). For short-term BCR activation the BCR was crosslinked for 10 min by soluble goat F(ab′)2 anti-human IgM (anti-IgM, Southern Biotechnology; 20 µg/ml) (2).

mRNA profiling (RNAseq) and data analysis

Total RNA was isolated by TRIzol (Molecular Research Center) as described previously (6). The sequencing of poly(A) transcriptome was performed as described previously (7). Briefly, libraries for samples CLL16, CLL39, CLL47, CLL50, CLL52-53, CLL60-65, and CLL68 were prepared using TruSeq Stranded mRNA LT Sample Prep Kit (Illumina) according to the manufacturer instructions. The final libraries were diluted to the desired concentration of 2 pM and were used to prepare a paired-end flow cell using a cBot System (Illumina) and then sequenced with Illumina NextSeq 500/550 High Output v2.5 kit (Illumina) generating 75-bp paired-end sequences. For samples CLL15, CLL19-23, and CLL43, libraries were prepared using TruSeq RNA Access Kit (Illumina) according to the manufacturer instructions. The final libraries were diluted to the desired concentration of 9 pM and sequenced with Illumina HiSeq 1000 (Illumina) generating 50-bp paired-end sequences.

The quality of the sequencing data was checked using the FastQC (8) (v0.11.3) and Kraken package (9) (v13-274). The presence of adapters was scanned using Minion and Swan (Kraken package, v13-274). Pre-processing of the raw reads was done using Trimmomatic (10) (v0.36) in the following steps (i) *N* and very low-quality bases (Phred < 3) from both 5' and 3' ends were removed, (ii) poly(A) and poly(T) tails were removed, (iii) sequencing adapters were removed, (iv) reads were trimmed to a maximal length of 75 bp, (v) low quality ends with average Phred score < 5 of 4 consecutive bases were trimmed using the sliding window approach, (vi) reads shorter than 15 bp and without a proper pairing after the pre-processing were removed. The pre-processed reads were then mapped to the *H. sapiens* reference genome (Ensembl release 84, primary assembly (11)) together with corresponding Ensembl genome annotation (Ensembl release 84) using STAR (12) (v2.5.2a) in 2-pass mapping approach. Exon-mapped reads were summarized to genes using STAR (a similar approach to the HTSeq-count (13) '*union*'). Only uniquely mapped reads were used. Strandedness of the sequencing protocol was considered and '*reverse*' counts were used. Differential gene expression was analyzed using DESeq2 (14) (v1.13.8) Bioconductor package for genes with a base mean count >200 per million. P-values were adjusted for multiple testing using the Benjamini-Hochberg method. Heatmaps were generated using matrix visualization software Morpheus by Broad Institute (RRID:SCR_017386). Data has been deposited at the European Genome-phenome Archive (EGA), which is hosted by the EBI and the CRG, under accession numbers EGAS50000000620 and EGAS50000000621.

BTK/PLCG2 variants were identified from RNAseq data as described previously (7). Briefly, quality check of raw paired-end fastq reads was carried out by FastQC v0.11.9 (15). The adapters were not removed due to their negligible amount. Quality trimming of raw fastq reads was performed using

Trimmomatic v0.39 (10). Cleaned RNA-Seq reads were mapped against the genomic reference GRCh38 using the STAR v2.7.3a (16). Mapped PCR duplicates were identified and removed using Picard v2.27.1 (17). The gene counts were produced using featureCounts from Subread v2.0 (18). Quality control after alignment concerning the number and percentage of uniquely- and multi-mapped reads, mapped regions, read coverage distribution, strand specificity, and PCR duplication was performed using several tools namely RSeQC v4.0.0 (19), Picard toolkit v2.25.6 and Qualimap v2.2.2 (20). Mutant-specific small variants (indels and SNVs) were called as somatic tumor-only. For aggregating calls from Strelka (21), VarDict (22), MuTect2 (23), and VarScan2 (24) in-house script was used. The resulting variants were annotated using Variant Effect Predictor (25).

Genome-wide binding of FoxO1 by CUT&RUN

CUT&RUN was done following the standard Janssens *et al*. protocol (26) with some modifications Nuclei from 0.5 million MEC1 cells were used per condition. Briefly, upon washes nuclei were extracted using a nuclear extraction buffer (20 mM Hepes pH 7.9, 10 mM KCl, 0.1% Triton X-100, 20% glycerol, 1 nM $MnCl_2$, and 0.5 mM spermidine) with Complete EDTA-free protease inhibitors (Roche), incubated 10 min on ice, and recovered by centrifugation 3 min, 600xg at 4 ºC. Nuclei were resuspended in 100 µl/sample nuclear extraction buffer and conjugated with 10 µl/sample of activated Concanavalin-coated magnetic beads (Bangs Laboratories). Binding of antibodies was performed overnight with 50 µl of antibody buffer (containing a final concentration of 0.01% of digitonin) and an antibody dilution of 1:50. Binding of pAG-Mnase was done for 2 hrs in 150 µl of buffer (containing 0.05% of digitonin) and 700 ng/ml of the pAG-Mnase protein (kindly shared with us by Dr Manuel Serrano, IRB, Barcelona, Spain). To start chromatin digestion freshly prepared 0.1 M $CaCl_2$ was added to each sample, mixed by pipetting and incubated for 30 min on ice. To stop the reaction 100 ul of Stop Buffer was used, followed by 30 min incubation at 37ºC with gentle mixing. Supernatant containing the digested chromatin was kept and DNA was extracted with Phenol Chloroform extraction.

Libraries were prepared following Nan Lui protocol(27) with the NEBNext Ultra II DNA library preparation kit (Cat# E7645S, Illumina) and were sequenced with Illumina NextSeq 500, High output v2.5 kit generating 75-bp paired-end sequences. The quality of the sequencing data was checked using FastQC (v0.11.9). Processing of the data was done using the cutruntools2.0 (28), using default parameters and the option of keeping 120 bp fragments. Peak calling was done using MACS2 (version 2.2.6), on deduplicated reads, using the IgG negative control (#5415, Cell Signaling). A cut off of 0.01 false discovery rate was used as a threshold and paired-end was taken into consideration. Genomic annotation of peaks was done using ChIPseeker R package. For motif analysis a window of +/- 200 bp around peak summits was analyzed using Centrimo (29) with default parameters.

Flow cytometry and cell sorting

Cell viability was investigated by DiOC6 (3,3′-dihexyloxacarbocyanine iodide) together with PI (propidium iodide) staining (Thermo Fisher Scientific) as described previously (30). Viability of thawed CLL cells from patients treated with ibrutinib in vivo and afterwards treated with FoxO1 inhibitor, ibrutinib or their combination in vitro was investigated by 7-AAD Viability Staining (eBioscience) together with DiOC6 staining (Thermo Fisher Scientific).

For cell cycle analysis, the cells were fixed in ice-cold 70% ethanol, washed twice with PBS, and stained with Vindelov's reagent (10mM TRIS pH 8.0, 10mM NaCl, 50 ng/mL propidium iodide, 40ng/mL RNase A) for 30 min at 37 °C. The cell cycle analysis was performed in FlowJov10.8.1 (FlowJo, LLC) using univariate Watson (Pragmatic) model.

For cell-surface staining, primary CLL cells or cell lines were labeled with one or combination of antibodies against: CD184 [CXCR4] (BV421, clone 12G5; BioLegend), CD20 (APC-eFluor 780, clone 2H7; eBioScience), CD5 (APC, clone L17F12; eBioScience). All measurements were performed on FACS Verse (BD Biosciences-US) and cell sorting on BD FACSAria Fusion (BD Biosciences-US).

Immunoblotting

Cells were lysed in lysis buffer (1% SDS, 50 mM TRIS-HCL pH 6.8, 10% glycerol) with phosphatase and protease inhibitors (Sigma Aldrich) and protein concentration was determined using DC Protein Assay (BioRad). Equal amounts of protein were separated by SDS-PAGE and transferred to the PVDF membrane (0.45 μm pore size, Millipore). The membranes were incubated with the Cell Signaling antibodies against the following immobilized proteins: Rictor (#2114, 1:2000), mTOR (#2983, 1:2000), pmTOR (#2974, 1:1000), FoxO1 (#2880, 1:2000), pFoxO1 (#2599, 1:1000), Akt (#2920, 1:2000), $pAkt^{S473}$ (#4060, 1:1000), GSK3α/β (#5676, 1:2000), $pAkt^{T308}$ (#13038, 1:1000), pBTK (#5082, 1:1000), BTK (#3533,1:2000), GAB1 (#3232, 1:2000), pGSK3α/β (#9331 1:2000), cMYC (#5605, 1:2000), GAPDH (#2118, 1:2000) or with anti-vinculin antibody (sc73614, 1:2000, Santa Cruz). Secondary horse-radish peroxidase (HRP)-conjugated anti-mouse or anti-rabbit antibodies (both Cell Signaling) were used to detect primary antibodies. Immunocomplexes were detected using ECL (BioRad), and the chemiluminescent signal was digitally detected with UVItec Alliance 4.7 (UVItec).

WST-1 assay

5×10^5 MEC1 cells or 5×10^4 primary CLL cells were seeded in 200 μl of media with addition of chosen inhibitors. After 48 hrs, 10 μl of Cell Proliferation Reagent WST-1 (#5015944001, Merck) was added to each condition. Cells were incubated for additional 0.5 to 4 hrs and the absorbance was measured according to the manufacturer's instructions.

CFSE proliferation assay

Purified CLL cells were loaded with cell trace dye (CSFE Dye, Invitrogen) as previously described (31). Briefly, up to 5×10^7 CLL cells were resuspended in 400 μl of PBS in 1.5 ml tube and 80 μl of PBS was put into the top of the tube. 0.5 μl of 5 mM CSFE dye was resuspended in the top and immediately mixed with the cell suspension. After 5 min incubation at room temperature, cells were washed two times with 5 % FBS in PBS. Cells were then seeded onto γ-irradiated $HS5^{wild\text{-}type}$ or $HS5^{CD40L,IL\text{-}4,IL\text{-}21}$ cells in a ratio 20:1 (CLL:HS5) (32), co-cultured with DMSO or inhibitors and analyzed 5 or 10 days later by flow cytometry. Proliferation was analyzed by Proliferation platform in FlowJo v10.8 with manual correction. Rate of proliferation was plotted as precursor frequency which represents the probability that a cell will divide at least once (33).

Cell line-derived xenograft mouse models

For competitive in vivo growth assay, $MEC1^{wild\text{-}type}$ and $MEC1^{FoxO1\text{-}KO}$ clones were traced with a plasmid encoding GFP or AZURIT, respectively (and *vice versa* to eliminate potential bias from constructs), and their equal numbers were mixed in a 1:1 ratio (1.5×10^6 per wild-type and the same number per FoxO1-KO clone) and injected into tail vein of NSG (NOD.Cg-$Prkdc^{scid}$ $Il2rg^{tm1Wjl}$/SzJ, The Jackson Laboratory) mice (2 males and 2 females, 5-month old). Mice were sacrificed 3 weeks after the MEC1 injection, and spleen, liver, blood, and bone marrow were analyzed for the ratio of GFP+:AZURIT+ cells by flow cytometry. The results were consistent irrespectively of the use of male vs female mice.

For in vivo testing of ibrutinib/FoxO1 inhibitor treatment, GFP positive MEC1 ($3x10^6$) were intravenously injected into female NRG mice (NOD.Cg-$Rag1^{tm1Mom}$ $Il2rg^{tm1Wjl}$/SzJ, The Jackson Laboratory, 2 to 3-month old). Female mice were selected to obtain maximal consistency in engraftment based on our previous experience, but the results should not be influenced by use of one animal sex. Mice were divided randomly after three days of leukemia engraftment into two groups and treated with either ibrutinib (n=9) or a combination of ibrutinib and FoxO1 inhibitor (n=9). Mice were sacrificed 14 days after the MEC1 injection, and bone marrow was analyzed for the percentage of GFP+ cells (schematic of the experiment in Figure A). Ibrutinib (MedChemExpress) was dissolved in drinking water with 1% HP-β-CD at a concentration 0.08 mg/ml, which is equivalent to a dose of 25 mg/kg/day. FoxO1 inhibitor (AS1842856, MedChemExpress) was dissolved (5% DMSO, 50% PEG-300, 5% Tween-80) and injected intraperitoneally daily at a dose of 30 mg/kg/day. Flow cytometry assessed the percentage of MEC1 cells in the organ as the percentage of GFP+ cells out of viable cells.

Statistical analysis

Apart from NGS analysis, all statistical analyses were performed with Prism v8.0.1 (GraphPad). Values of $P< 0.05$ were considered statistically significant. Data in graphs represent mean ± SEM. Enrichment of signaling pathways was performed using Enrichr tool (34) (the Ma'ayan Lab) visualizing KEGG pathways 2021.

2) Supplemental Figures

Supplemental Figure 1

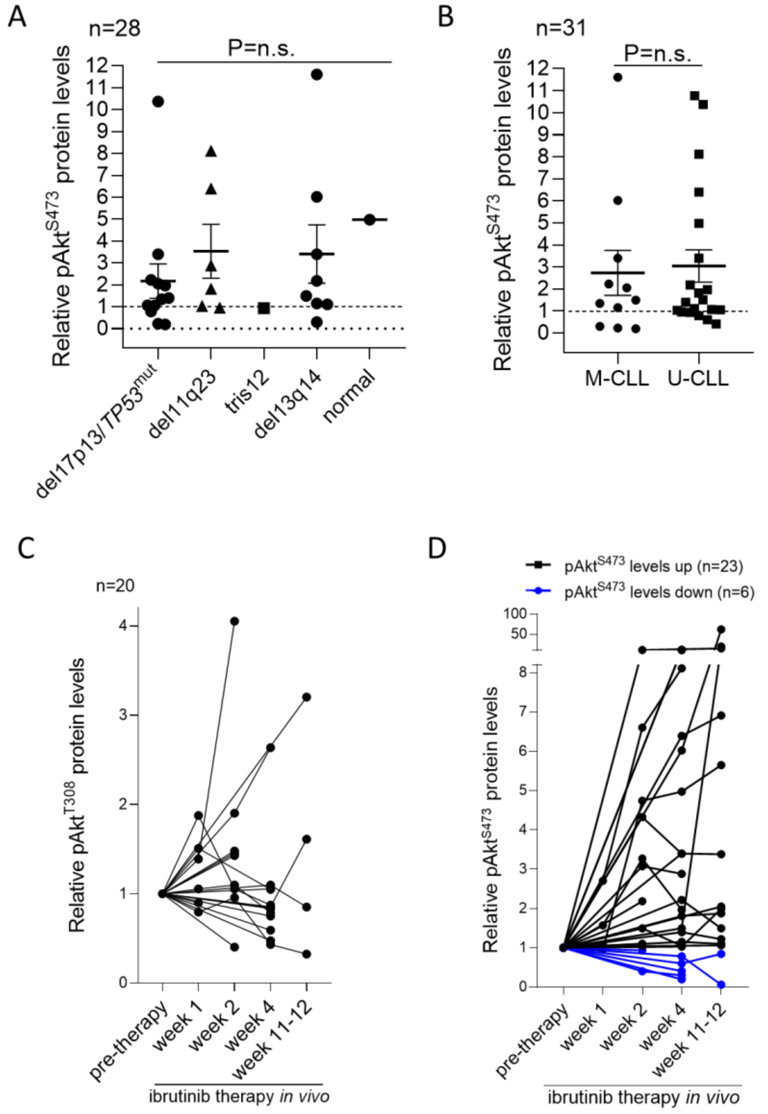

Supplemental Figure 1. (A) Relative pAkt^{S473} protein levels in CLL samples obtained during ibrutinib treatment in vivo and stratified according to FISH abnormalities (a hierarchical Dohner classification). Statistical difference was calculated using one-way ANOVA. **(B)** Relative pAkt^{S473} protein levels in CLL samples obtained during ibrutinib treatment in vivo and stratified according to IGHV status. M-CLL ≤98% homology with germinal IGHV sequence; U-CLL >98% homology with germinal IGHV sequence. Statistical difference was calculated using unpaired t-test. **(C)** Densitometric quantification of relative pAkt^{T308} protein levels in primary CLL samples obtained before and during ibrutinib treatment in vivo (n=20). **(D)** Relative pAkt^{S473} protein levels in CLL samples obtained during ibrutinib treatment in vivo whose relative lymphocyte counts (ALC) are shown in Fig. 1E. Black lines indicate samples with upregulated/stable pAkt^{S473} levels (n=23), blue lines indicate samples with downregulated pAkt^{S473} levels (n=6).

Supplemental Figure 2

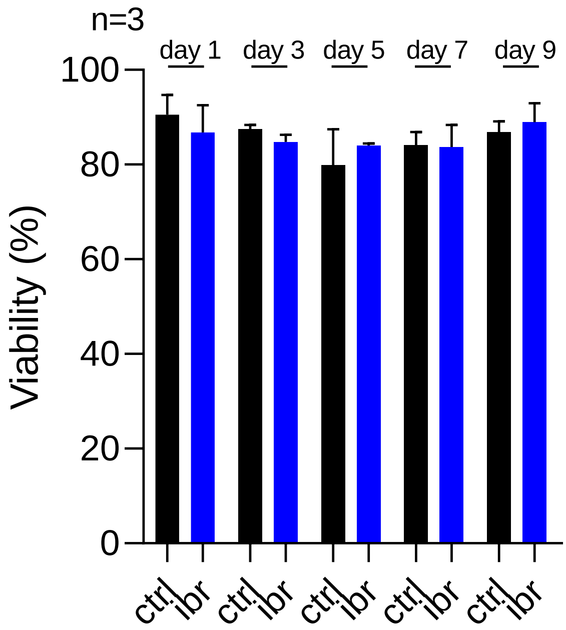

Supplemental Figure 2. Viability of MEC1 cells treated with ibrutinib in vitro as compared to vehicle (2 µM, n=3, 1-9 days).

Supplemental Figure 3

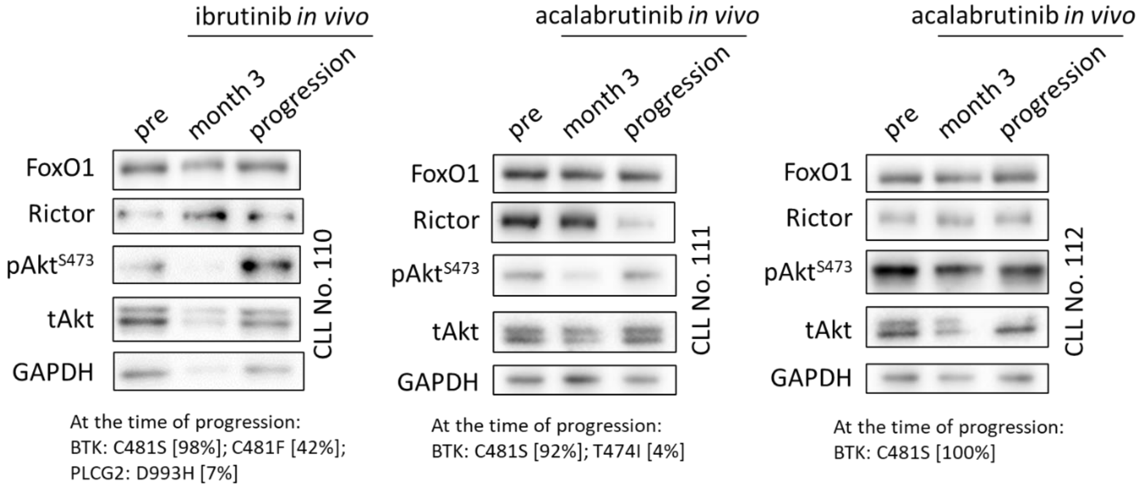

Supplemental Figure 3. Immunoblots of primary CLL samples before and after 3 months of BTK inhibitor therapy (n=1 for ibrutinib, n=2 for acalabrutinib) and at the time of progression with *BTK* and *PLCG2* mutation status indicated at the bottom (variant allele frequency in brackets).

Supplemental Figure 4

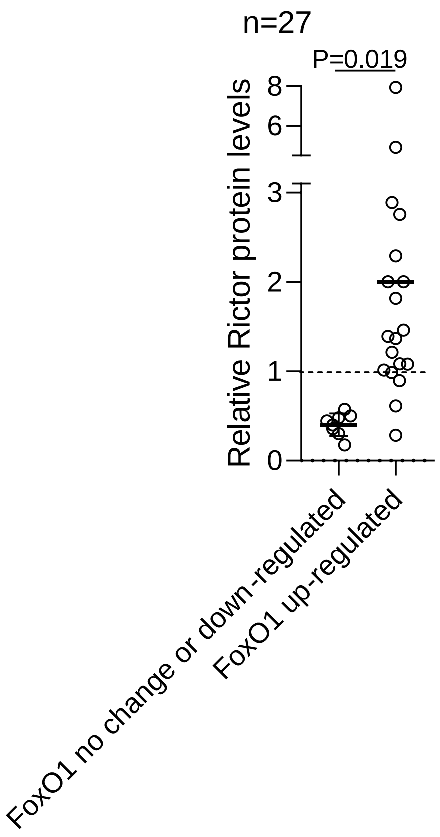

Supplemental Figure 4. Relative Rictor protein levels after ibrutinib therapy in vivo in patients with downregulation of FoxO1 (fold change < 0.9), no change of FoxO1 levels (fold change 0.9-1.1), or upregulation of FoxO1 (fold change > 1.1) after therapy. P-value was calculated using unpaired t-test.

Supplemental Figure 5

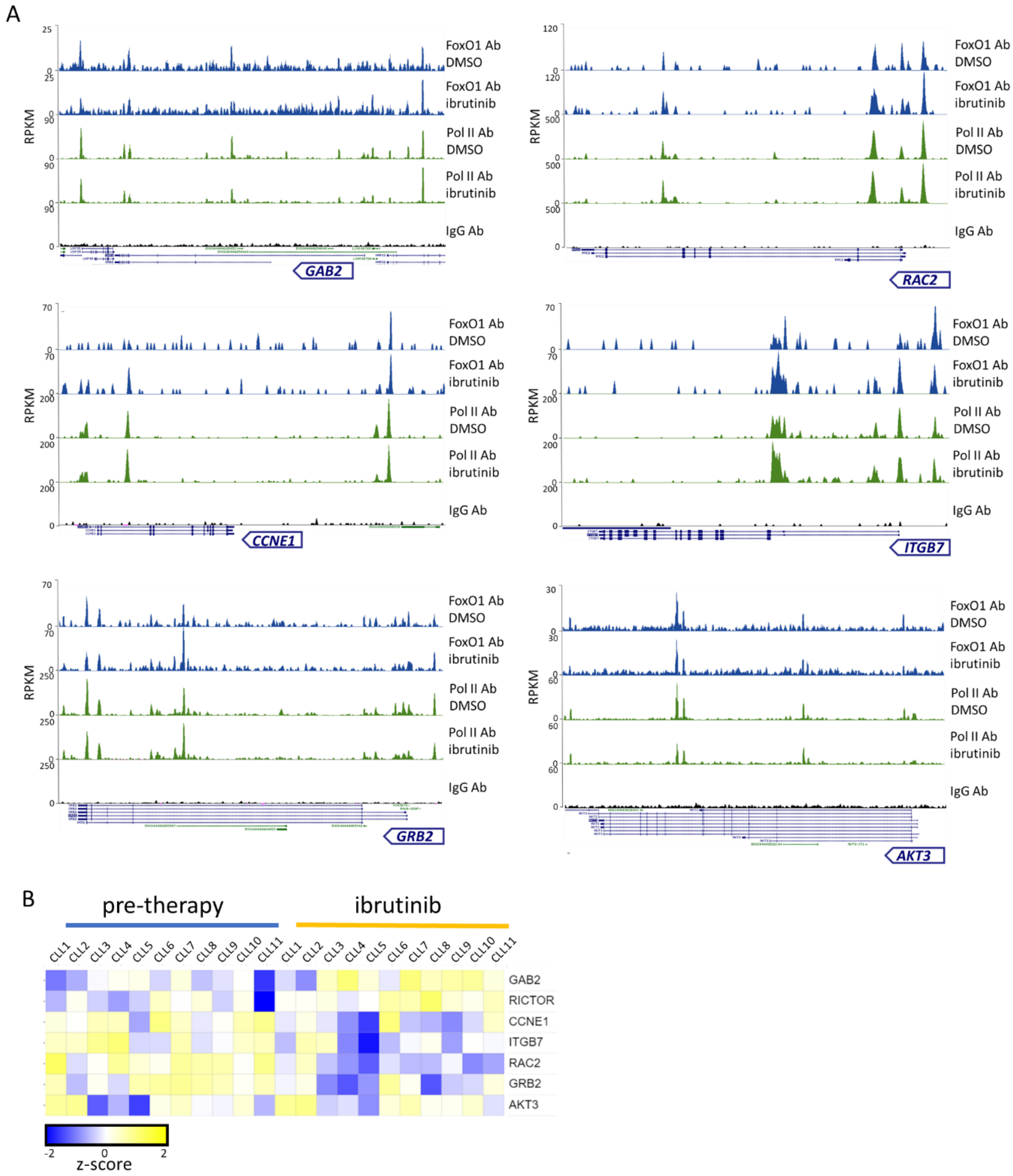

Supplemental Figure 5. (A) The peaks of FoxO1 binding (CUT&RUN) in regulatory regions of selected genes in MEC1 cells treated with vehicle DMSO (FoxO1 Ab DMSO) or MEC1 cells treated with ibrutinib (FoxO1 Ab ibrutinib; 1 μM, 6 days). The selected genes are involved in PI3K/Akt pathway and are significantly changed during ibrutinib therapy in vivo (see [B]). RPKM stands for reads per kilobase per transcript per million reads mapped. **(B)** Heatmap of selected FoxO1 bound differentially expressed genes (see [A]) from samples obtained before and after ibrutinib treatment in vivo (P adj < 0.05; base mean > 100; n=11 pairs). Lower expression is indicated in blue, higher expression in yellow.

Supplemental Figure 6

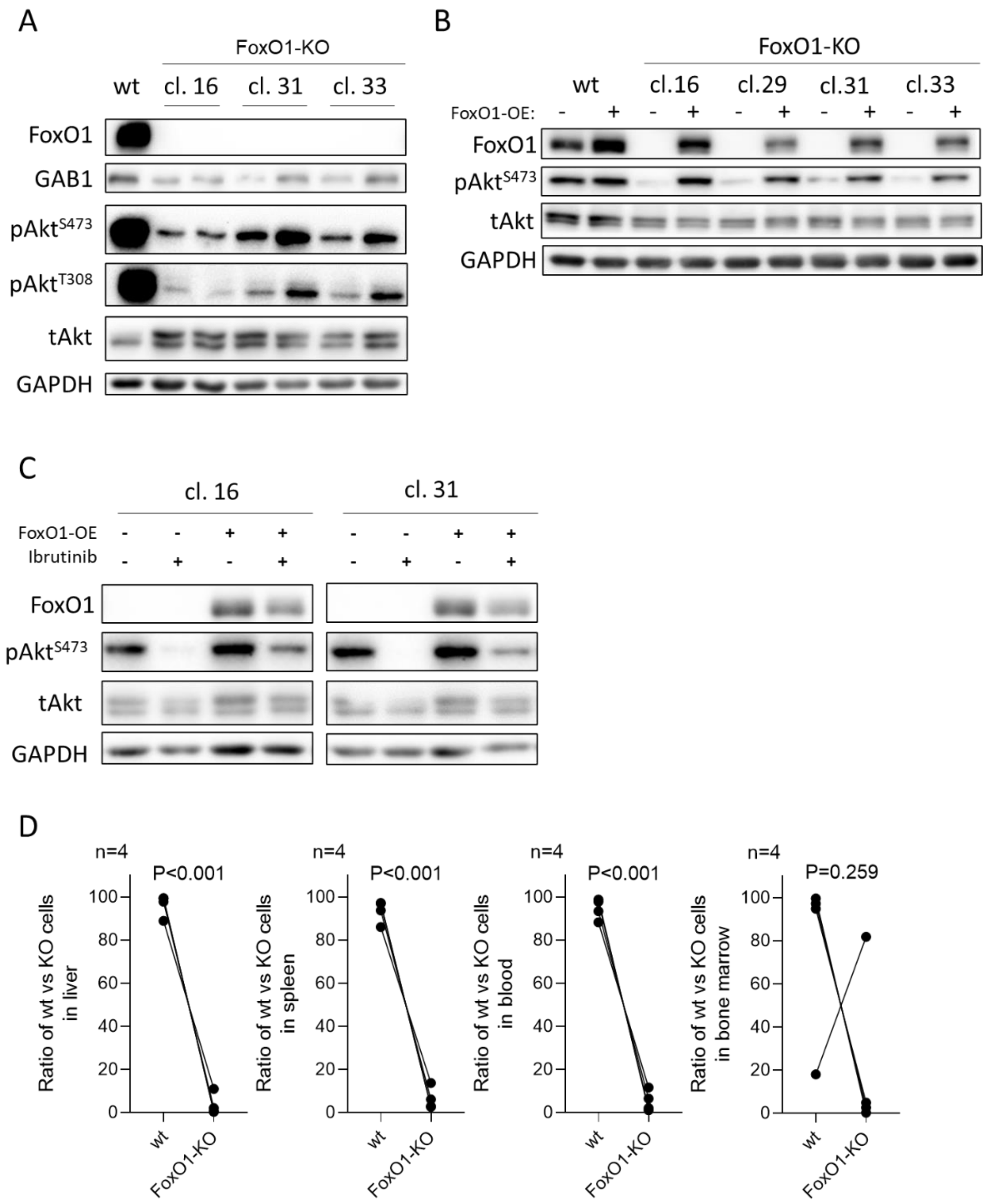

Supplemental Figure 6. (A) Representative immunoblot of *FoxO1*-KO MEC1 clones (cl. 16, 31, 33) in comparison to control wild-type MEC1 cells. **(B)** Representative immunoblot of wild-type and *FoxO1*-KO MEC1 clones 48 hrs after transfection with empty plasmid (FoxO1-OE "-") or plasmid overexpressing constitutively active FoxO1 (FoxO1-OE "+"). **(C)** *FoxO1*-KO MEC1 clones (cl. 16 and 31) transduced with doxycycline-inducible empty or FoxO1 overexpressing (FoxO1-OE) plasmid treated with doxycycline (0.5 µg/ml) and ibrutinib (2 µM) or vehicle for 5 days. **(D)** Relative number of cells in a competitive growth assay of $MEC1^{wt}$ and $MEC1^{FoxO1\text{-}KO}$ (clones 29 and 31). $MEC1^{wt}$ and $MEC1^{FoxO1\text{-}KO}$ were traced with GFP or AZURIT (or vice versa) and mixed in 1:1 ratio (1.5×10^6 per clone) and injected into the tail vein of NSG mice (n=4, n=2 per clone [29 or 31]). Mice were sacrificed after 3 weeks, and the ratio of $MEC1^{wt}$ vs. $MEC1^{FoxO1\text{-}KO}$ in liver, spleen, blood, and bone marrow was assessed by flow cytometry. P-values were calculated using paired t-test.

Supplemental Figure 7

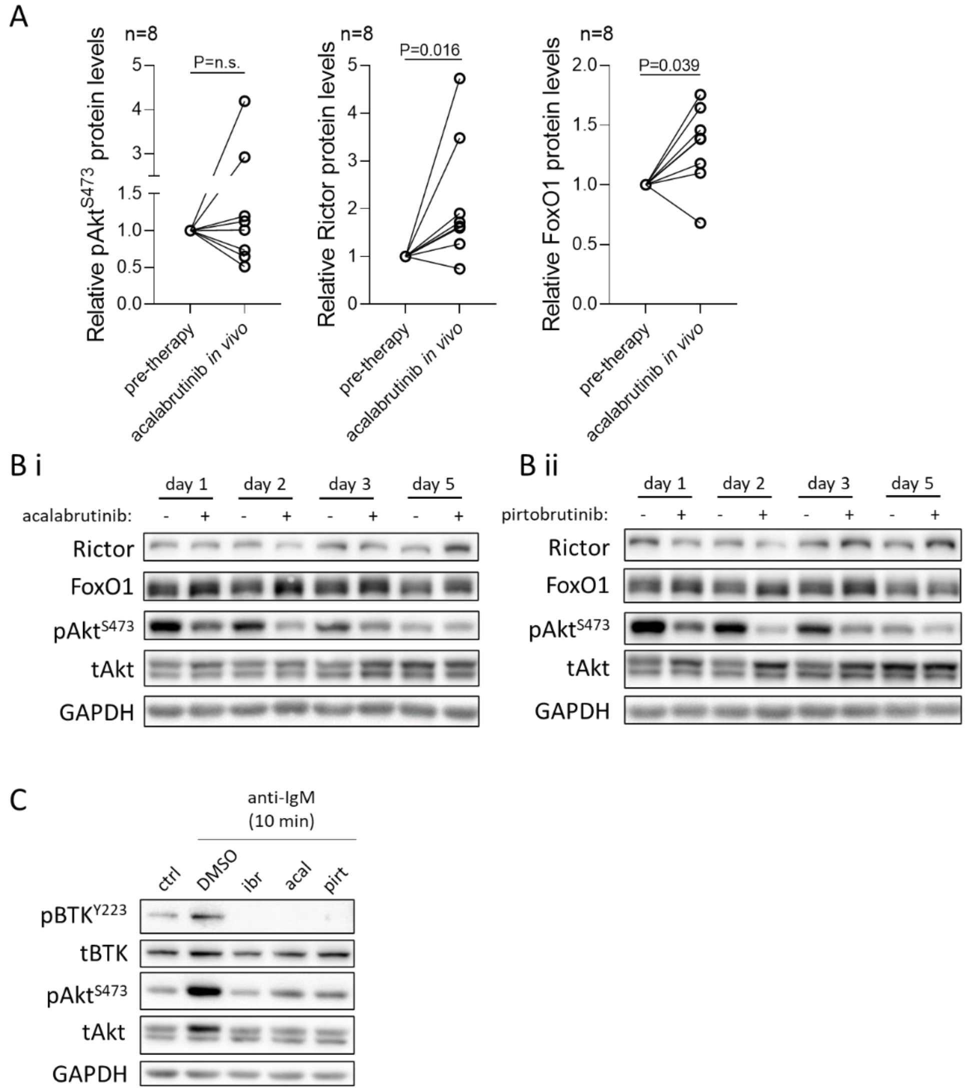

Supplemental Figure 7. (A) Densitometric quantification of relative pAkt^{S473}, Rictor and FoxO1 protein level analyzed by immunoblot in primary samples obtained from CLL patients before and during treatment with acalabrutinib in vivo (1-3 months, n=8). P-values were calculated using Wilcoxon test. **(B)** Representative immunoblot of MEC1 cells treated with **[i]** acalabrutinib (5 μM, 1-5 days) or **[ii]** pirtobrutinib (2 μM, 1-5 days). **(C)** Representative immunoblot of MEC1 cells treated with ibrutinib (ibr, 2 μM), acalabrutinib (acal, 2 μM), pirtobrutinib (pirt, 2 μM), or vehicle for 24 hrs and then BCR-stimulated with soluble anti-IgM (10 min, 10 μg/ml). For patient characteristics, see supplemental Table 8.

Supplemental Figure 8

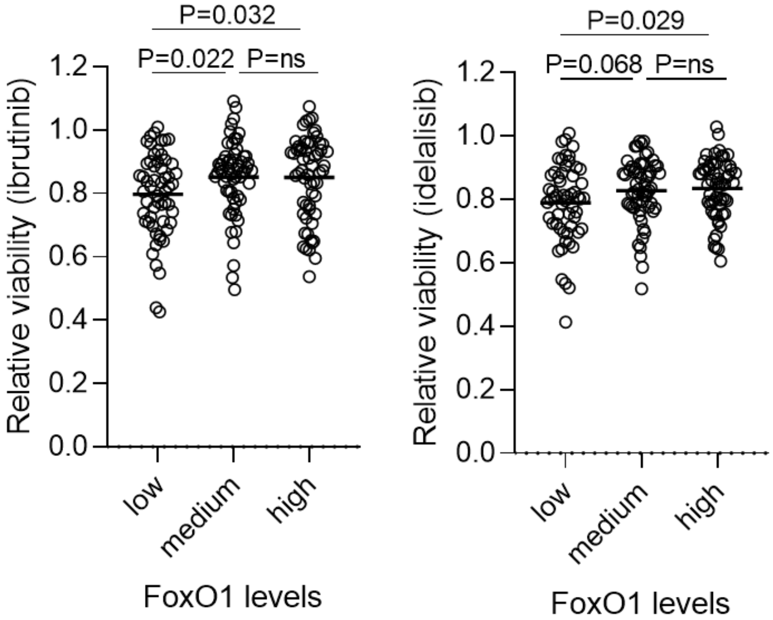

Supplemental Figure 8. Relative viability of primary CLL cells treated with ibrutinib (left) or idelalisib (right) in vitro (both 2 μM) divided into terciles based on *FoxO1* mRNA levels. Data were extracted from Lu *et al.*, 2022 (35). P-values were calculated using unpaired t-test.

Supplemental Figure 9

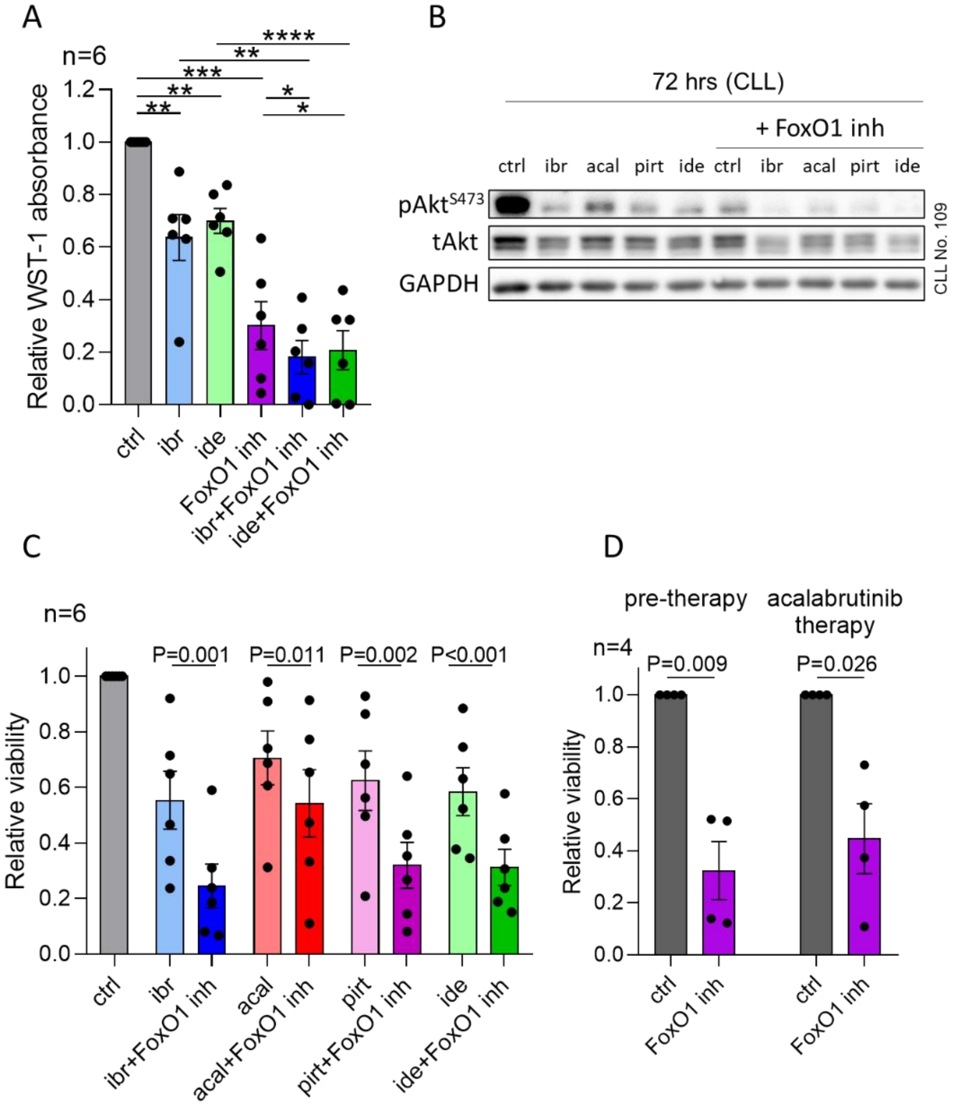

Supplemental Figure 9. (A) Relative WST-1 absorbance in CLL cells (n=6) treated with ibrutinib (ibr, 1 µM), idelalisib (ide, 1 µM), FoxO1 inhibitor (FoxO1 inh, 0.5 µM), or their combination (48 hrs). * stands for P≤0.05; ** stands for P≤0.01; *** stands for P≤0.001; **** stands for P≤0.0001. **(B)** Representative immunoblot of primary CLL cells pre-treated with ibrutinib (ibr), acalabrutinib (acal), pirtobrutinib (pirt) or idelalisib (ide; all 1 µM) for 24 hrs and subsequently treated with FoxO1 inhibitor (FoxO1 inh, 0.5 µM) added to the culture for an additional 48 hrs. **(C)** Relative viability of primary CLL cells (n=6) pre-treated with ibrutinib (ibr), acalabrutinib (acal), pirtobrutinib (pirt) or idelalisib (ide; all 1 µM) for 24 hrs and subsequently treated with FoxO1 inhibitor (FoxO1 inh, 0.5 µM) added to the culture for an additional 48 hrs. **(D)** Relative viability of paired CLL samples (n=4) obtained before and during 1 (n=2) or 3 months (n=2) of acalabrutinib therapy in vivo. Cells were treated with FoxO1 inhibitor (FoxO1 inh, 0.5 µM) for 72 hrs. For patient characteristics, see supplemental Table 8. P-values were calculated using paired t-test throughout the whole Supplemental Figure 9.

Supplemental Figure 10

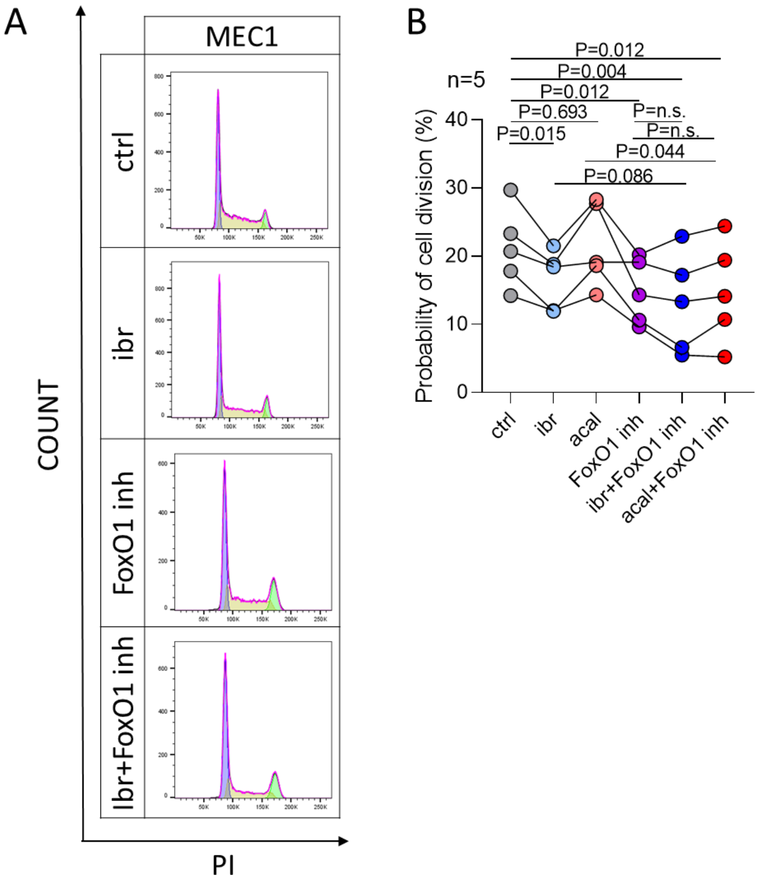

Supplemental Figure 10. (A) Representative histograms of cell cycle analysis (PI staining) of MEC1 cells treated with ibrutinib (ibr, 2 µM), or FoxO1 inhibitor (FoxO1 inh, 0.5 µM), or their combination (96 hrs). **(B)** Proliferation of primary CLL cells (n=5) indicated by the probability that cells will divide at least once (calculated from precursor frequency). CLL cells were co-cultured with stromal cells $HS5^{CD40L,IL\text{-}4,IL\text{-}21}$ and treated with ibrutinib (ibr, 1 µM), acalabrutinib (acal, 1 µM), or FoxO1 inhibitor (FoxO1 inh, 0.5 µM), or their combination for 5 days. P-values were calculated using paired t-test. For patient characteristics, see supplemental Table 8.

Supplemental Figure 11

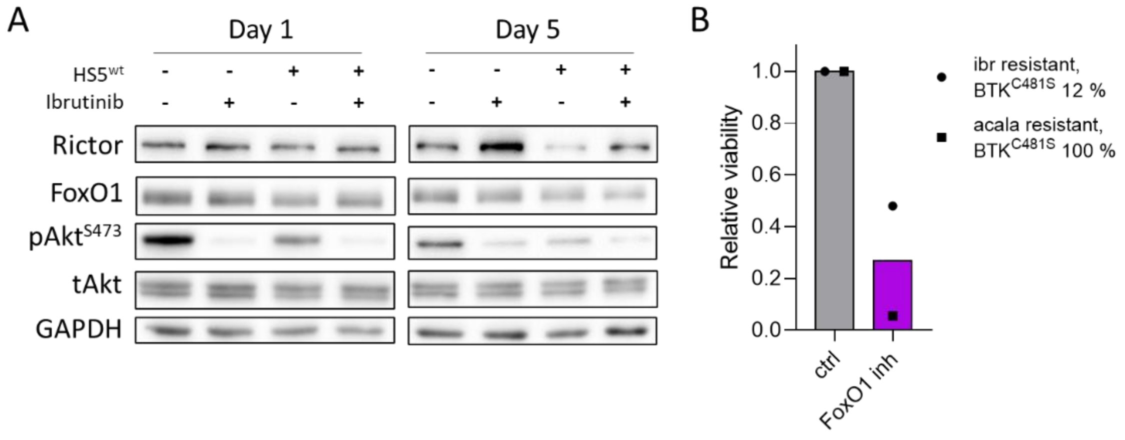

Supplemental Figure 11. (A) Representative immunoblot of MEC1 cells treated with ibrutinib (2 µM) and cultured on plastic or in co-culture with irradiated $HS5^{WT}$ cells (1 and 5 days). MEC1 cells were seeded in 3:1 ratio (MEC1:HS5). **(B)** Relative viability of primary CLL cells obtained for patients at the time of progression on BTK inhibitors treated with FoxO1 inhibitor (FoxO1 inh, 0.5 µM) for 72 hrs.

Supplemental Figure 12

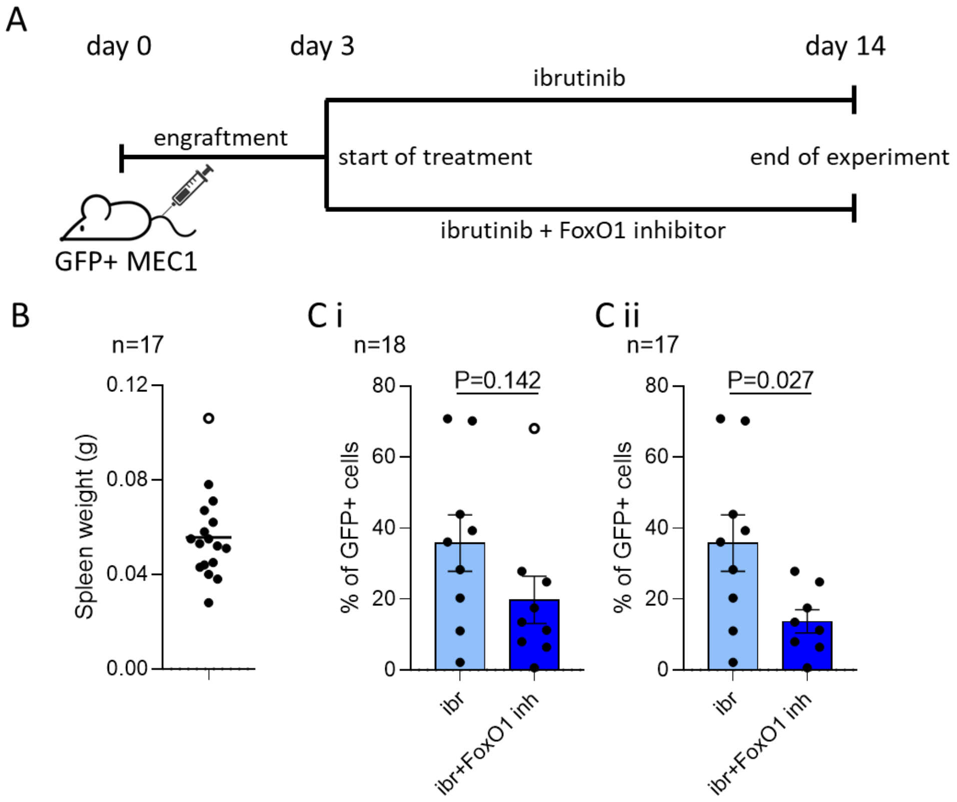

Supplemental Figure 12. (A) Scheme of the in vivo experiment with MEC1 cells transplanted to immunodeficient NRG mice. **(B)** Spleen weight in mice used in the experiment (n=17, spleen weight of 1 mouse unknown). An open circle marks the outlier organ (spleen) identified statistically by Grubbs method (Alpha = 0.05). **(C)** Percentage of viable GFP positive MEC1 cells (7-AAD negative) in bone marrow of NRG mice treated by ibrutinib (ibr, 25 mg/kg/day) or combination of ibrutinib (25 mg/kg/day) plus FoxO1 inhibitor (FoxO1 inh, 30 mg/kg/day). The **(Ci)** represents all 18 mice, and **(Cii)** represents 17 mice with the exclusion of the one outlier mouse in [B]. An open circle in [Ci] marks the outlier mice. P-values were calculated using unpaired t-test.

Supplemental Figure 13

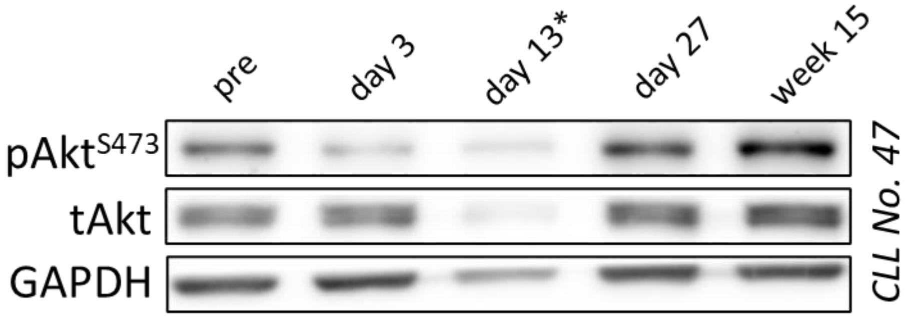

Supplemental Figure 13. Representative pAkt^{S473} immunoblot of a primary CLL sample obtained before and during ibrutinib treatment in vivo, including a very early time point (day 3).
*sample from day 13 (on therapy) is likely underloaded on the immunoblot, but the immunoblot could not be repeated due to a lack of material.

Supplemental Figure 14

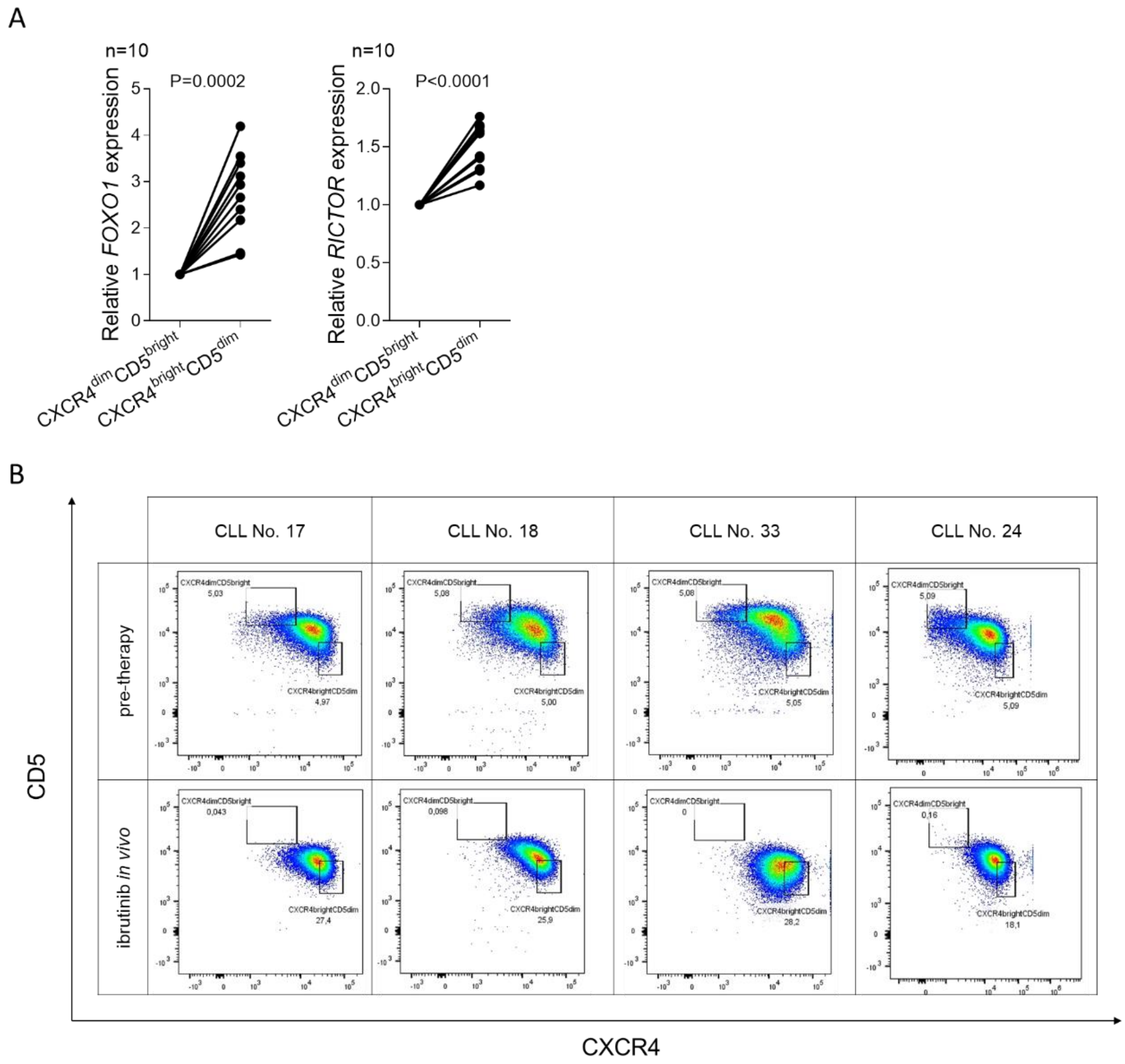

Supplemental Figure 14. (A) Relative expression of *FoxO1* and *Rictor* mRNAs in CXCR4/CD5 subpopulations obtained in paired samples from 10 patients. A sorting strategy for obtaining CXCR4/CD5 subpopulations was described previously (2, 5). P-values were calculated using paired t-test. **(B)** The shifts in intraclonal CXCR4/CD5 subpopulations in CLL samples (n=4) obtained before (top) or during ibrutinib therapy in vivo (3-8 weeks of therapy; bottom).

3) Supplemental Tables

Supplemental Table 1: List of top differentially expressed genes during ibrutinib therapy in vivo (top 250 upregulated and top 250 downregulated mRNAs)

Gene ID	Gene name	Base Mean (reads per million)	Fold Change (log2)*	Adjusted P-value
ENSG00000165507	C10orf10	103.2	1.217992	1.35E-05
ENSG00000152953	STK32B	360.0	0.931744	0.001187
ENSG00000180287	PLD5	345.0	0.931189	4.69E-05
ENSG00000161714	PLCD3	175.7	0.892518	0.0014
ENSG00000099958	DERL3	603.0	0.86836	0.000167
ENSG00000070882	OSBPL3	1296.9	0.848972	0.001361
ENSG00000010278	CD9	197.4	0.825038	0.001156
ENSG00000205710	C17orf107	315.1	0.800993	0.000265
ENSG00000134245	WNT2B	333.6	0.792452	0.000904
ENSG00000171246	NPTX1	1276.6	0.788703	0.006577
ENSG00000179088	C12orf42	393.6	0.779687	0.004003
ENSG00000159433	STARD9	3326.0	0.771129	0.00015
ENSG00000102053	ZC3H12B	628.2	0.766312	0.000289
ENSG00000166348	USP54	391.0	0.752302	8.45E-05
ENSG00000272398	CD24	5504.8	0.748053	6.55E-05
ENSG00000184271	POU6F1	612.5	0.740701	4.69E-05
ENSG00000151623	NR3C2	1175.2	0.739431	0.007305
ENSG00000128596	CCDC136	126.6	0.733246	0.008348
ENSG00000204001	LCN8	504.2	0.731636	0.00479
ENSG00000150672	DLG2	1390.6	0.706398	0.00479
ENSG00000118432	CNR1	429.7	0.674792	0.01467
ENSG00000178075	GRAMD1C	512.6	0.671551	0.007832
ENSG00000161040	FBXL13	144.6	0.667591	0.003264
ENSG00000197044	ZNF441	2237.0	0.665786	0.000672
ENSG00000187792	ZNF70	373.2	0.664856	0.007385
ENSG00000198932	GPRASP1	4305.3	0.657235	0.003067
ENSG00000168026	TTC21A	3640.6	0.655718	0.000171
ENSG00000174469	CNTNAP2	449.5	0.648414	0.038996
ENSG00000139304	PTPRQ	215.7	0.64829	0.010489
ENSG00000100490	CDKL1	2166.2	0.645073	0.013967
ENSG00000076555	ACACB	2157.4	0.644478	0.000426
ENSG00000184226	PCDH9	8123.5	0.634127	0.023167
ENSG00000050030	KIAA2022	232.0	0.630462	0.033253
ENSG00000131042	LILRB2	998.7	0.627954	0.000351
ENSG00000111266	DUSP16	187.2	0.625274	0.033241
ENSG00000123411	IKZF4	158.4	0.618365	0.006111
ENSG00000117020	AKT3	1472.6	0.609996	0.035401
ENSG00000186350	RXRA	2001.6	0.609032	0.025436
ENSG00000187240	DYNC2H1	1091.9	0.608358	0.007804
ENSG00000170482	SLC23A1	303.8	0.606358	0.003594
ENSG00000188026	RILPL1	148.8	0.605288	0.020521
ENSG00000101236	RNF24	233.8	0.604484	0.004385
ENSG00000113369	ARRDC3	11321.5	0.602083	0.00012
ENSG00000110318	CEP126	616.2	0.601834	0.014077
ENSG00000262621	LA16c-306E5.2	113.4	0.601029	0.016819
ENSG00000182463	TSHZ2	341.2	0.600725	0.019711
ENSG00000265972	TXNIP	94590.5	0.588013	0.003612
ENSG00000108984	MAP2K6	1906.7	0.585139	0.005653
ENSG00000256574	OR13A1	310.5	0.583124	0.001861
ENSG00000165029	ABCA1	10492.4	0.581847	0.023203
ENSG00000168477	TNXB	359.7	0.577742	0.033591
ENSG00000169762	TAPT1	3206.5	0.573893	0.00125

ENSG00000173530	TNFRSF10D	570.9	0.572443	0.014084
ENSG00000159788	RGS12	142.4	0.57075	0.016646
ENSG00000145864	GABRB2	1565.7	0.570059	0.022619
ENSG00000112182	BACH2	7294.8	0.569345	0.000267
ENSG00000170915	PAQR8	255.3	0.568654	0.038441
ENSG00000138641	HERC3	6792.1	0.5621	0.012299
ENSG00000170049	KCNAB3	145.5	0.558673	0.023072
ENSG00000118263	KLF7	266.4	0.558263	0.005557
ENSG00000164300	SERINC5	5085.7	0.557253	0.027353
ENSG00000135749	PCNXL2	1913.6	0.555857	0.02176
ENSG00000185800	DMWD	588.4	0.555252	0.004777
ENSG00000105948	TTC26	417.6	0.547296	0.000406
ENSG00000055118	KCNH2	913.7	0.54726	0.011077
ENSG00000100629	CEP128	475.9	0.546792	0.009957
ENSG00000152818	UTRN	56338.7	0.545833	0.003571
ENSG00000101846	STS	274.5	0.544243	1.35E-05
ENSG00000150907	FOXO1	3453.0	0.542957	0.000456
ENSG00000178917	ZNF852	942.6	0.542681	0.036825
ENSG00000082269	FAM135A	802.7	0.542014	0.00066
ENSG00000213923	CSNK1E	4612.6	0.541285	0.004421
ENSG00000176438	SYNE3	4738.7	0.540995	0.002266
ENSG00000033327	GAB2	4616.3	0.539883	0.001197
ENSG00000198879	SFMBT2	633.2	0.539576	0.024628
ENSG00000131697	NPHP4	327.3	0.538899	0.000584
ENSG00000165115	KIF27	1052.5	0.537889	0.006288
ENSG00000234284	ZNF879	366.0	0.537848	0.036438
ENSG00000163611	SPICE1	2157.6	0.536863	0.011442
ENSG00000072364	AFF4	12339.9	0.535352	0.012698
ENSG00000196712	NF1	7960.4	0.535228	0.000286
ENSG00000146776	ATXN7L1	873.6	0.533652	0.002006
ENSG00000164970	FAM219A	284.4	0.530713	0.001135
ENSG00000206530	CFAP44	2375.2	0.52926	0.006817
ENSG00000170222	ADPRM	862.3	0.527325	1.04E-05
ENSG00000106537	TSPAN13	506.1	0.527293	0.033975
ENSG00000125844	RRBP1	10655.6	0.523047	0.020721
ENSG00000126705	AHDC1	833.2	0.522966	0.014924
ENSG00000146757	ZNF92	11403.6	0.522492	0.029026
ENSG00000197841	ZNF181	956.9	0.521547	0.001923
ENSG00000131375	CAPN7	3522.1	0.518943	0.011614
ENSG00000163346	PBXIP1	6916.4	0.517252	0.007653
ENSG00000163001	CFAP36	514.9	0.513344	0.020547
ENSG00000138443	ABI2	1256.9	0.513276	0.002659
ENSG00000066923	STAG3	412.4	0.512728	0.017803
ENSG00000181074	OR52N4	252.5	0.511234	0.006388
ENSG00000196074	SYCP2	869.4	0.509674	0.001187
ENSG00000144645	OSBPL10	6584.8	0.504423	0.012967
ENSG00000146535	GNA12	2765.4	0.500055	0.004462
ENSG00000160961	ZNF333	1113.6	0.498869	0.017863
ENSG00000188636	LDOC1L	208.6	0.498661	0.031418
ENSG00000184384	MAML2	4107.7	0.498261	0.021419
ENSG00000174516	PELI3	165.0	0.497848	0.03275
ENSG00000180855	ZNF443	433.8	0.497835	0.031031
ENSG00000185591	SP1	5239.9	0.4968	0.005145
ENSG00000138078	PREPL	3382.4	0.495485	0.00517
ENSG00000001167	NFYA	1626.7	0.494984	0.001502
ENSG00000160201	U2AF1	310.7	0.494403	0.004536
ENSG00000160199	PKNOX1	740.2	0.49405	0.002736
ENSG00000134460	IL2RA	2330.4	0.493945	0.03385
ENSG00000122126	OCRL	1205.0	0.49331	0.002595
ENSG00000111752	PHC1	987.4	0.49199	0.021407

ENSG00000114859	CLCN2	237.3	0.491197	5.89E-05
ENSG00000004399	PLXND1	1851.8	0.491155	0.025615
ENSG00000147044	CASK	5384.3	0.490916	0.000204
ENSG00000083168	KAT6A	10727.1	0.490613	0.036279
ENSG00000145990	GFOD1	2396.7	0.490445	0.040702
ENSG00000135482	ZC3H10	254.0	0.489345	0.034664
ENSG00000124126	PREX1	10056.7	0.489288	0.001156
ENSG00000136451	VEZF1	2314.2	0.489224	3.58E-05
ENSG00000108557	RAI1	2674.2	0.489221	0.011414
ENSG00000166435	XRRA1	784.7	0.488806	0.002871
ENSG00000138002	IFT172	1045.2	0.487143	0.007396
ENSG00000072134	EPN2	158.1	0.485377	0.03385
ENSG00000136870	ZNF189	1193.4	0.484981	0.022448
ENSG00000144445	KANSL1L	1367.6	0.484079	0.024004
ENSG00000196172	ZNF681	1015.9	0.482274	0.01844
ENSG00000165959	CLMN	4182.0	0.481872	0.045158
ENSG00000158321	AUTS2	1426.4	0.480441	0.000324
ENSG00000116127	ALMS1	11227.5	0.479909	0.032248
ENSG00000156313	RPGR	2455.8	0.479453	0.0063
ENSG00000166762	CATSPER2	1692.7	0.477297	0.002403
ENSG00000133812	SBF2	2360.8	0.476597	0.004633
ENSG00000118922	KLF12	2014.6	0.475517	0.000111
ENSG00000130338	TULP4	2563.6	0.473296	0.009957
ENSG00000148429	USP6NL	3495.8	0.471528	0.001235
ENSG00000093072	CECR1	4109.7	0.471494	0.000262
ENSG00000171130	ATP6V0E2	306.3	0.470564	0.043874
ENSG00000101605	MYOM1	1179.8	0.469797	0.024525
ENSG00000107614	TRDMT1	427.5	0.468194	0.012967
ENSG00000100197	CYP2D6	126.6	0.464538	0.031502
ENSG00000167037	SGSM1	2772.8	0.46369	0.043765
ENSG00000196440	ARMCX4	769.0	0.46349	0.004914
ENSG00000132510	KDM6B	4239.9	0.461477	0.032822
ENSG00000154122	ANKH	882.8	0.460336	0.009274
ENSG00000164663	USP49	213.7	0.457665	0.000659
ENSG00000126970	ZC4H2	709.7	0.457582	0.015976
ENSG00000104375	STK3	185.8	0.457383	0.045992
ENSG00000170145	SIK2	891.3	0.457211	0.00517
ENSG00000204262	COL5A2	655.7	0.455773	0.047628
ENSG00000189180	ZNF33A	5426.5	0.455483	0.003438
ENSG00000198887	SMC5	4811.6	0.454904	0.002882
ENSG00000171606	ZNF274	4314.6	0.454362	0.020465
ENSG00000143322	ABL2	1417.1	0.453674	0.001991
ENSG00000189144	ZNF573	490.2	0.453576	0.042446
ENSG00000182134	TDRKH	159.2	0.453181	0.024351
ENSG00000139278	GLIPR1	1163.9	0.453048	0.006864
ENSG00000143669	LYST	38161.0	0.452706	0.023167
ENSG00000149292	TTC12	1486.6	0.452647	0.000289
ENSG00000100784	RPS6KA5	1372.8	0.451861	0.001704
ENSG00000196263	ZNF471	599.1	0.446267	0.01294
ENSG00000170448	NFXL1	1007.6	0.446166	0.019397
ENSG00000136828	RALGPS1	4614.4	0.44427	0.018201
ENSG00000167202	TBC1D2B	513.2	0.443767	0.000491
ENSG00000161996	WDR90	267.7	0.443085	0.038996
ENSG00000136144	RCBTB1	1583.5	0.443062	0.032437
ENSG00000123104	ITPR2	19063.3	0.44133	0.036508
ENSG00000166263	STXBP4	548.7	0.440741	0.017806
ENSG00000127334	DYRK2	1456.3	0.440107	0.00479
ENSG00000141068	KSR1	486.6	0.43851	0.047628
ENSG00000163291	PAQR3	280.6	0.437775	0.001284
ENSG00000171988	JMJD1C	33907.4	0.436705	0.045772

ENSG00000143970	ASXL2	4216.4	0.436563	0.026391
ENSG00000143924	EML4	10953.2	0.435329	0.000534
ENSG00000204569	PPP1R10	3748.0	0.43436	0.036438
ENSG00000048707	VPS13D	26851.6	0.43249	0.032962
ENSG00000198752	CDC42BPB	976.7	0.432001	0.001852
ENSG00000196369	SRGAP2B	420.9	0.431559	0.017117
ENSG00000188171	ZNF626	963.0	0.43146	0.031719
ENSG00000187824	TMEM220	235.3	0.430899	0.032888
ENSG00000166501	PRKCB	38714.2	0.430875	0.006699
ENSG00000140386	SCAPER	5973.0	0.430845	0.001346
ENSG00000174428	GTF2IRD2B	1586.5	0.429942	0.006995
ENSG00000239521	GATS	1560.6	0.429624	0.025298
ENSG00000186260	MKL2	2746.3	0.429219	0.031418
ENSG00000125827	TMX4	2988.3	0.428564	0.002878
ENSG00000140543	DET1	242.8	0.428488	0.044889
ENSG00000135093	USP30	639.8	0.42831	0.018571
ENSG00000173273	TNKS	4668.6	0.427366	0.017972
ENSG00000123374	CDK2	445.8	0.424963	0.009086
ENSG00000172915	NBEA	3199.1	0.424904	0.045092
ENSG00000109790	KLHL5	3082.2	0.424135	0.042084
ENSG00000124374	PAIP2B	513.7	0.423044	0.005959
ENSG00000221968	FADS3	1839.6	0.422568	0.004517
ENSG00000102043	MTMR8	769.0	0.422155	0.01473
ENSG00000116539	ASH1L	19469.5	0.42112	0.049766
ENSG00000128881	TTBK2	4762.3	0.420958	0.018584
ENSG00000130518	KIAA1683	3896.3	0.420024	0.033604
ENSG00000130684	ZNF337	2252.8	0.419499	0.014152
ENSG00000137504	CREBZF	3813.3	0.419468	0.031467
ENSG00000157796	WDR19	3686.7	0.417247	0.040498
ENSG00000164953	TMEM67	672.7	0.417069	0.022635
ENSG00000112599	GUCA1B	164.5	0.416927	0.031799
ENSG00000251369	ZNF550	898.1	0.416038	0.007832
ENSG00000134013	LOXL2	419.8	0.415928	0.045158
ENSG00000166432	ZMAT1	4357.1	0.414914	0.03444
ENSG00000198453	ZNF568	807.9	0.414536	0.007874
ENSG00000074755	ZZEF1	8808.7	0.412376	0.029647
ENSG00000133030	MPRIP	5002.0	0.412277	0.003741
ENSG00000149115	TNKS1BP1	310.1	0.411955	0.025182
ENSG00000107611	CUBN	2673.6	0.411563	0.010967
ENSG00000174738	NR1D2	2327.0	0.411412	0.002926
ENSG00000111666	CHPT1	5025.7	0.40934	0.022332
ENSG00000177683	THAP5	938.8	0.408136	0.038565
ENSG00000116977	LGALS8	1294.6	0.407521	5.55E-05
ENSG00000163378	EOGT	802.4	0.406624	0.001059
ENSG00000089280	FUS	38768.7	0.406296	0.002882
ENSG00000010404	IDS	2305.9	0.405822	0.000367
ENSG00000113658	SMAD5	1767.7	0.405324	0.02294
ENSG00000176225	RTTN	2923.7	0.40507	0.014844
ENSG00000134109	EDEM1	8386.2	0.403641	0.042641
ENSG00000114904	NEK4	1565.7	0.40332	0.009925
ENSG00000165169	DYNLT3	336.8	0.402793	0.040772
ENSG00000145687	SSBP2	1651.8	0.401934	0.006836
ENSG00000136141	LRCH1	3576.3	0.40184	0.015117
ENSG00000125633	CCDC93	4087.2	0.401676	0.014969
ENSG00000008300	CELSR3	1115.8	0.400711	0.023669
ENSG00000144357	UBR3	7209.8	0.400169	0.021407
ENSG00000135541	AHI1	3891.6	0.399936	0.002128
ENSG00000172765	TMCC1	782.9	0.39818	0.016764
ENSG00000110888	CAPRIN2	3762.4	0.397866	0.000424
ENSG00000119699	TGFB3	336.0	0.397364	0.02082

ENSG00000147894	C9orf72	1464.0	0.397313	0.000555
ENSG00000173889	PHC3	19618.2	0.395639	0.031467
ENSG00000167703	SLC43A2	1994.7	0.394992	0.038795
ENSG00000232653	GOLGA8N	239.5	0.393011	0.019068
ENSG00000018189	RUFY3	2330.9	0.392992	0.042231
ENSG00000164440	TXLNB	367.2	0.392	0.02983
ENSG00000204130	RUFY2	2202.7	0.391038	0.006775
ENSG00000214413	BBIP1	719.8	0.390755	0.014438
ENSG00000182022	CHST15	3316.8	0.390753	0.024525
ENSG00000106479	ZNF862	1697.4	0.389262	0.000324
ENSG00000176155	CCDC57	828.5	0.386807	0.016491
ENSG00000104427	ZC2HC1A	817.7	0.386654	0.015757
ENSG00000067182	TNFRSF1A	620.9	0.385869	0.044309
ENSG00000169057	MECP2	3278.1	0.385623	0.026826
ENSG00000120948	TARDBP	2639.3	0.384526	0.00988
ENSG00000166704	ZNF606	1132.0	0.384454	0.027254
ENSG00000107371	EXOSC3	501.3	0.383346	0.047628
ENSG00000151422	FER	1551.5	0.383168	0.045158
ENSG00000185201	IFITM2	1175.8	-0.50686	0.011857
ENSG00000166145	SPINT1	523.5	-0.50692	0.005368
ENSG00000175334	BANF1	1313.1	-0.50733	0.023023
ENSG00000166446	CDYL2	357.8	-0.50771	0.049884
ENSG00000146094	DOK3	955.1	-0.50825	0.036279
ENSG00000145088	EAF2	888.9	-0.50989	0.036077
ENSG00000137767	SQRDL	382.1	-0.51077	0.038795
ENSG00000178035	IMPDH2	1947.0	-0.5112	0.013267
ENSG00000162302	RPS6KA4	301.1	-0.51398	0.002595
ENSG00000136573	BLK	24967.5	-0.51494	0.001731
ENSG00000176101	SSNA1	525.8	-0.5161	0.026472
ENSG00000065485	PDIA5	146.4	-0.5178	0.006575
ENSG00000167797	CDK2AP2	314.8	-0.51791	0.035071
ENSG00000139193	CD27	2701.4	-0.51817	0.000265
ENSG00000143653	SCCPDH	795.8	-0.5187	0.003474
ENSG00000138758	SEPT11	646.7	-0.51939	0.018727
ENSG00000134508	CABLES1	596.1	-0.51951	0.045772
ENSG00000079999	KEAP1	249.1	-0.51975	0.02406
ENSG00000132185	FCRLA	4510.8	-0.52065	0.005707
ENSG00000103415	HMOX2	515.7	-0.52127	0.015123
ENSG00000111816	FRK	705.8	-0.52192	0.037784
ENSG00000139725	RHOF	397.1	-0.52358	0.044566
ENSG00000111639	MRPL51	296.9	-0.52444	0.014712
ENSG00000168899	VAMP5	642.2	-0.52478	0.012787
ENSG00000125457	MIF4GD	832.4	-0.52547	0.012967
ENSG00000110697	PITPNM1	1697.2	-0.52693	0.000802
ENSG00000072954	TMEM38A	110.6	-0.529	0.014221
ENSG00000105364	MRPL4	609.4	-0.52902	0.000235
ENSG00000100271	TTLL1	160.3	-0.53083	0.013031
ENSG00000099822	HCN2	112.7	-0.53241	0.006229
ENSG00000164088	PPM1M	2029.0	-0.53246	0.000659
ENSG00000100348	TXN2	598.1	-0.53323	0.006288
ENSG00000023191	RNH1	1960.6	-0.53364	0.023135
ENSG00000124256	ZBP1	758.4	-0.53466	0.035119
ENSG00000141905	NFIC	408.1	-0.53478	3.99E-05
ENSG00000169442	CD52	8950.5	-0.53611	0.00988
ENSG00000146232	NFKBIE	1069.7	-0.53777	0.004536
ENSG00000166394	CYB5R2	152.6	-0.53803	0.032561
ENSG00000111328	CDK2AP1	160.0	-0.53888	0.023167
ENSG00000141858	SAMD1	272.1	-0.53963	0.023277
ENSG00000111716	LDHB	2682.8	-0.54001	0.003571
ENSG00000149781	FERMT3	2060.6	-0.54001	0.000119

ENSG00000157193	LRP8	257.6	-0.54055	0.032981
ENSG00000196743	GM2A	2694.8	-0.54079	0.000376
ENSG00000185522	LMNTD2	214.0	-0.54242	0.006487
ENSG00000136810	TXN	418.8	-0.54327	0.037385
ENSG00000103653	CSK	6537.2	-0.54409	0.000144
ENSG00000160446	ZDHHC12	182.1	-0.5457	0.02777
ENSG00000116663	FBXO6	158.0	-0.54663	0.042446
ENSG00000170684	ZNF296	156.9	-0.54673	0.029625
ENSG00000103528	SYT17	1074.9	-0.54675	0.005195
ENSG00000198938	MT-CO3	55166.7	-0.54752	0.000412
ENSG00000154589	LY96	310.4	-0.54798	0.024853
ENSG00000172824	CES4A	384.7	-0.54855	0.018229
ENSG00000167751	KLK2	267.9	-0.54973	0.035157
ENSG00000138629	UBL7	1195.3	-0.55002	0.004763
ENSG00000256269	HMBS	419.1	-0.55292	0.018229
ENSG00000118640	VAMP8	1202.5	-0.55332	0.004385
ENSG00000148180	GSN	444.7	-0.55427	0.037844
ENSG00000051180	RAD51	204.0	-0.55508	0.041093
ENSG00000239713	APOBEC3G	2981.8	-0.55516	0.016939
ENSG00000166557	TMED3	459.5	-0.5596	0.003067
ENSG00000085117	CD82	2296.9	-0.56	0.001062
ENSG00000067836	ROGDI	191.1	-0.56114	0.002313
ENSG00000160094	ZNF362	539.6	-0.56229	0.010344
ENSG00000156253	RWDD2B	119.6	-0.5649	0.028472
ENSG00000243477	NAT6	181.2	-0.56666	0.038795
ENSG00000204392	LSM2	373.9	-0.56709	0.011829
ENSG00000163932	PRKCD	2206.7	-0.57012	0.001649
ENSG00000076604	TRAF4	2642.3	-0.57265	0.00986
ENSG00000243811	APOBEC3D	1529.2	-0.57413	0.000493
ENSG00000069399	BCL3	883.3	-0.57582	0.007305
ENSG00000086730	LAT2	488.7	-0.57679	0.000456
ENSG00000028137	TNFRSF1B	4686.6	-0.57903	0.001533
ENSG00000164674	SYTL3	328.5	-0.57934	0.033058
ENSG00000137880	GCHFR	292.0	-0.58154	0.031118
ENSG00000204475	NCR3	314.5	-0.58203	0.02983
ENSG00000188677	PARVB	466.5	-0.58208	0.026391
ENSG00000063241	ISOC2	123.5	-0.58441	0.02983
ENSG00000198736	MSRB1	197.3	-0.5846	0.000324
ENSG00000107833	NPM3	163.2	-0.58531	0.044462
ENSG00000087266	SH3BP2	2711.4	-0.58673	0.009382
ENSG00000087448	KLHL42	325.3	-0.58844	0.000266
ENSG00000100889	PCK2	625.3	-0.58965	0.001163
ENSG00000030582	GRN	6823.0	-0.58993	0.004653
ENSG00000101255	TRIB3	136.9	-0.5915	0.007721
ENSG00000141337	ARSG	288.1	-0.59263	0.024351
ENSG00000187994	RINL	558.0	-0.59627	0.007832
ENSG00000152689	RASGRP3	8035.9	-0.59698	0.003844
ENSG00000103089	FA2H	199.8	-0.59765	0.026991
ENSG00000108515	ENO3	464.9	-0.59786	0.004738
ENSG00000233276	GPX1	1292.6	-0.5985	0.007914
ENSG00000160999	SH2B2	472.0	-0.59876	0.011611
ENSG00000136732	GYPC	787.0	-0.59879	0.001156
ENSG00000109971	HSPA8	17927.6	-0.6006	0.00098
ENSG00000135925	WNT10A	306.4	-0.60084	0.023203
ENSG00000197629	MPEG1	1924.9	-0.60204	0.023444
ENSG00000154217	PITPNC1	1185.6	-0.6024	0.020743
ENSG00000240505	TNFRSF13B	864.2	-0.60256	0.007121
ENSG00000157593	SLC35B2	1225.1	-0.60553	0.004003
ENSG00000137101	CD72	1913.9	-0.60838	0.007259
ENSG00000113758	DBN1	178.9	-0.60964	0.031969

ENSG00000137309	HMGA1	1625.7	-0.60986	0.005871
ENSG00000165644	COMTD1	100.3	-0.61062	0.038795
ENSG00000146192	FGD2	3145.6	-0.61155	0.001163
ENSG00000103152	MPG	515.0	-0.61204	0.002603
ENSG00000198715	GLMP	392.0	-0.61404	2.02E-05
ENSG00000142512	SIGLEC10	1771.4	-0.61485	0.004517
ENSG00000105369	CD79A	17443.6	-0.6175	0.001133
ENSG00000151725	CENPU	277.6	-0.61787	0.000716
ENSG00000172292	CERS6	485.9	-0.61863	0.018229
ENSG00000137936	BCAR3	471.9	-0.61958	0.001156
ENSG00000111669	TPI1	7524.2	-0.61967	0.000617
ENSG00000131747	TOP2A	159.0	-0.6199	0.034761
ENSG00000121807	CCR2	235.2	-0.62063	0.036867
ENSG00000169508	GPR183	1226.1	-0.62154	0.009104
ENSG00000178741	COX5A	706.4	-0.62319	0.002561
ENSG00000065057	NTHL1	145.1	-0.62419	0.005978
ENSG00000177106	EPS8L2	241.4	-0.62942	0.011981
ENSG00000102878	HSF4	105.0	-0.63132	0.017704
ENSG00000122862	SRGN	3538.5	-0.63225	0.005281
ENSG00000254709	IGLL5	1929.5	-0.63264	0.003799
ENSG00000169413	RNASE6	405.6	-0.63407	0.014844
ENSG00000171533	MAP6	372.6	-0.63426	0.041002
ENSG00000005884	ITGA3	428.5	-0.63642	0.001649
ENSG00000182199	SHMT2	3250.4	-0.6367	0.000456
ENSG00000151651	ADAM8	799.7	-0.63855	0.026516
ENSG00000213585	VDAC1	1302.3	-0.63999	0.001166
ENSG00000056736	IL17RB	298.7	-0.64079	0.036438
ENSG00000116586	LAMTOR2	976.4	-0.64101	0.001594
ENSG00000161013	MGAT4B	2624.3	-0.64173	0.00106
ENSG00000103257	SLC7A5	375.6	-0.64291	0.009086
ENSG00000125454	SLC25A19	480.2	-0.64324	0.000626
ENSG00000142765	SYTL1	2303.7	-0.64394	0.001991
ENSG00000002745	WNT16	196.8	-0.64589	0.021862
ENSG00000062524	LTK	1176.4	-0.64613	0.012967
ENSG00000158481	CD1C	103.5	-0.64755	0.031502
ENSG00000122176	FMOD	5110.4	-0.64785	0.038795
ENSG00000163808	KIF15	191.7	-0.6498	0.017888
ENSG00000138623	SEMA7A	322.0	-0.65104	0.028984
ENSG00000277632	CCL3	414.2	-0.65357	0.010694
ENSG00000173531	MST1	185.0	-0.6537	6.55E-05
ENSG00000166886	NAB2	153.1	-0.6551	0.014221
ENSG00000140105	WARS	2526.6	-0.65709	0.003391
ENSG00000011478	QPCTL	208.7	-0.65903	0.002491
ENSG00000136261	BZW2	480.8	-0.65935	0.001833
ENSG00000182481	KPNA2	827.6	-0.66006	0.000111
ENSG00000157470	FAM81A	118.1	-0.66023	0.020219
ENSG00000042753	AP2S1	480.8	-0.66055	0.009453
ENSG00000155368	DBI	2430.6	-0.66069	0.005981
ENSG00000100292	HMOX1	1430.8	-0.66148	0.008617
ENSG00000104998	IL27RA	523.9	-0.66152	0.000672
ENSG00000151552	QDPR	193.3	-0.66453	0.003997
ENSG00000142252	GEMIN7	440.1	-0.66475	0.000609
ENSG00000108578	BLMH	535.8	-0.66759	0.002595
ENSG00000146066	HIGD2A	818.0	-0.66877	3.58E-05
ENSG00000077782	FGFR1	535.2	-0.6703	0.021407
ENSG00000166689	PLEKHA7	338.6	-0.67093	0.024004
ENSG00000275395	FCGBP	1780.8	-0.67777	0.014924
ENSG00000128487	SPECC1	180.0	-0.67955	0.005827
ENSG00000148908	RGS10	745.5	-0.68001	0.002128
ENSG00000174123	TLR10	375.2	-0.6812	0.023167

ENSG00000126264	HCST	437.0	-0.68379	0.016646
ENSG00000160856	FCRL3	5575.5	-0.68797	0.023203
ENSG00000162144	CYB561A3	5319.5	-0.68861	0.000227
ENSG00000105492	SIGLEC6	176.7	-0.68918	0.025174
ENSG00000122694	GLIPR2	245.4	-0.69159	0.013497
ENSG00000167930	FAM234A	206.7	-0.69271	0.006435
ENSG00000130598	TNNI2	160.2	-0.69966	0.002739
ENSG00000162241	SLC25A45	1148.9	-0.7017	0.000173
ENSG00000042493	CAPG	1334.4	-0.703	0.005537
ENSG00000155090	KLF10	555.1	-0.70327	0.005145
ENSG00000198712	MT-CO2	41091.6	-0.70593	1.35E-05
ENSG00000139880	CDH24	108.7	-0.70753	0.014682
ENSG00000198840	MT-ND3	14449.5	-0.70894	6.55E-05
ENSG00000188191	PRKAR1B	203.2	-0.70963	0.000193
ENSG00000134333	LDHA	4492.1	-0.71219	0.000698
ENSG00000136026	CKAP4	2444.9	-0.71397	0.000807
ENSG00000135776	ABCB10	2208.9	-0.71563	0.000109
ENSG00000167264	DUS2	2742.6	-0.71819	0.000382
ENSG00000106868	SUSD1	640.9	-0.7189	0.004286
ENSG00000112137	PHACTR1	700.5	-0.72291	0.001594
ENSG00000128604	IRF5	1235.4	-0.72822	0.000111
ENSG00000159399	HK2	612.5	-0.72823	0.003064
ENSG00000197888	UGT2B17	497.6	-0.73601	0.011442
ENSG00000111679	PTPN6	20080.3	-0.73672	0.002301
ENSG00000137571	SLCO5A1	200.1	-0.73844	0.012617
ENSG00000162631	NTNG1	167.2	-0.74183	0.012596
ENSG00000112759	SLC29A1	311.8	-0.74516	0.005104
ENSG00000198176	TFDP1	981.2	-0.7493	0.00033
ENSG00000011590	ZBTB32	1600.7	-0.75001	0.002006
ENSG00000120875	DUSP4	177.1	-0.75457	0.012596
ENSG00000189159	HN1	310.9	-0.7598	0.003223
ENSG00000151014	NOCT	104.6	-0.76026	0.001631
ENSG00000166825	ANPEP	116.9	-0.76034	0.01096
ENSG00000112320	SOBP	240.4	-0.76493	0.008742
ENSG00000130590	SAMD10	142.2	-0.76948	2.63E-05
ENSG00000126246	IGFLR1	234.1	-0.77108	0.004536
ENSG00000187608	ISG15	207.6	-0.77111	0.00442
ENSG00000112149	CD83	2738.7	-0.7712	0.000356
ENSG00000105173	CCNE1	278.2	-0.77502	0.000498
ENSG00000177685	CRACR2B	291.9	-0.77892	0.006966
ENSG00000165996	HACD1	184.0	-0.78151	0.004214
ENSG00000146386	ABRACL	167.3	-0.7831	0.000124
ENSG00000166510	CCDC68	104.3	-0.7831	0.002739
ENSG00000196923	PDLIM7	172.2	-0.78606	0.000373
ENSG00000120708	TGFBI	6427.9	-0.78821	0.003474
ENSG00000104921	FCER2	10884.1	-0.79264	0.000456
ENSG00000124772	CPNE5	1287.3	-0.79714	0.007462
ENSG00000101187	SLCO4A1	576.4	-0.80239	0.003571
ENSG00000188389	PDCD1	199.8	-0.80712	0.000311
ENSG00000130429	ARPC1B	2532.3	-0.80892	0.000172
ENSG00000177697	CD151	158.6	-0.80894	0.00479
ENSG00000128340	RAC2	7805.1	-0.80898	0.000111
ENSG00000065911	MTHFD2	546.1	-0.81097	6.55E-05
ENSG00000183765	CHEK2	570.2	-0.81124	0.00012
ENSG00000226979	LTA	168.3	-0.81609	0.00089
ENSG00000112667	DNPH1	262.9	-0.81644	0.000134
ENSG00000147535	PLPP5	1975.7	-0.82109	0.002926
ENSG00000167483	FAM129C	7332.6	-0.8311	7.53E-05
ENSG00000196189	SEMA4A	2926.0	-0.83593	1.73E-05
ENSG00000049449	RCN1	151.1	-0.84425	0.001429

ENSG00000101460	MAP1LC3A	724.6	-0.84758	0.00132
ENSG00000141753	IGFBP4	1284.4	-0.86353	0.000111
ENSG00000137460	FHDC1	508.1	-0.87005	1.6E-06
ENSG00000179841	AKAP5	128.3	-0.87563	0.001939
ENSG00000278195	SSTR3	105.5	-0.87706	0.002739
ENSG00000168734	PKIG	1028.3	-0.87818	1.04E-05
ENSG00000011028	MRC2	140.8	-0.89058	0.002266
ENSG00000115956	PLEK	3839.3	-0.89322	9.57E-05
ENSG00000108797	CNTNAP1	1126.8	-0.89533	1.75E-05
ENSG00000175274	TP53I11	927.0	-0.90588	0.000182
ENSG00000158050	DUSP2	547.3	-0.90817	0.0014
ENSG00000048462	TNFRSF17	121.9	-0.93473	0.00026
ENSG00000163599	CTLA4	4363.4	-0.96152	1.84E-05
ENSG00000140678	ITGAX	6410.3	-0.97909	0.000109
ENSG00000102575	ACP5	1331.3	-1.00003	1.65E-05
ENSG00000083454	P2RX5	595.1	-1.00465	3.99E-05
ENSG00000162896	PIGR	1501.0	-1.01605	0.000271
ENSG00000156738	MS4A1	11298.8	-1.01789	7.28E-06
ENSG00000116771	AGMAT	191.1	-1.02132	6.55E-05
ENSG00000168081	PNOC	495.5	-1.02224	3.99E-05
ENSG00000186810	CXCR3	160.7	-1.03704	0.000171
ENSG00000157388	CACNA1D	729.0	-1.04794	0.00015
ENSG00000186818	LILRB4	587.1	-1.06133	9.85E-05
ENSG00000115129	TP53I3	314.2	-1.06889	3.36E-05
ENSG00000189058	APOD	635.5	-1.06897	3.99E-05
ENSG00000064886	CHI3L2	328.7	-1.07918	0.000108
ENSG00000173621	LRFN4	191.2	-1.11393	1.35E-05
ENSG00000239961	LILRA4	511.1	-1.25426	6.95E-07

*250 most up-regulated genes (positive fold change) and 250 most down-regulated genes (negative fold change) during ibrutinib therapy in vivo

Supplemental Table 2: GO term analysis of differentially expressed genes during ibrutinib therapy in vivo

GO term*	Description	P-value	FDR q-value	Enrichment	N	B	n	b
GO:0030155	regulation of cell adhesion	5.47E-05	3.57E-01	2.55	1010	50	182	23
GO:0007165	signal transduction	1.21E-04	3.94E-01	1.53	1010	276	177	74
GO:1904889	regulation of excitatory synapse assembly	2.59E-04	5.63E-01	84.17	1010	2	12	2
GO:0002250	adaptive immune response	2.66E-04	4.33E-01	2.19	1010	22	378	18
GO:2000479	regulation of cAMP-dependent protein kinase activity	3.36E-04	4.38E-01	17.12	1010	3	59	3
GO:0061041	regulation of wound healing	3.43E-04	3.72E-01	6.16	1010	5	164	5
GO:0006955	immune response	5.04E-04	4.69E-01	2.5	1010	68	125	21
GO:0042594	response to starvation	6.67E-04	5.43E-01	3	1010	12	281	10
GO:0050853	B cell receptor signaling pathway	8.70E-04	6.30E-01	2.43	1010	10	416	10
GO:0002768	immune response-regulating cell surface receptor signaling pathway	9.52E-04	6.20E-01	2.44	1010	37	201	18
GO:0010677	negative regulation of cellular carbohydrate metabolic process	9.73E-04	5.77E-01	31.56	1010	2	32	2
GO:0045912	negative regulation of carbohydrate metabolic process	9.73E-04	5.29E-01	31.56	1010	2	32	2
GO:0034164	negative regulation of toll-like receptor 9 signaling pathway	9.90E-04	4.97E-01	1	10	1010	1	1
GO:0034156	negative regulation of toll-like receptor 7 signaling pathway	9.90E-04	4.61E-01	1	10	1010	1	1
GO:0034155	regulation of toll-like receptor 7 signaling pathway	9.90E-04	4.30E-01	1	10	1010	1	1
GO:0032687	negative regulation of interferon-alpha production	9.90E-04	4.03E-01	1	10	1010	1	1

*GO Term analysis was performer by GOrilla online tool and showed GO Terms are referring to group „Processes“.

Enrichment = (b/n) / (B/N)

N - is the total number of genes
B - is the total number of genes associated with a specific GO term
n - is the number of genes in the top of the user's input genes
b - is the number of genes in the intersection

Supplemental Table 3: List of genes involved in PI3K/Akt signalling whose expression is changed during ibrutinib therapy in vivo (adjusted P-value < 0.05; base mean > 100).

Gene ID	Gene name	Base Mean (reads per million)	Fold Change (log2)*	Adjusted P-value
ENSG00000049089	COL9A2	7063.7	-0.50467	0.000111
ENSG00000128340	RAC2	7805.1	-0.80898	0.000111
**ENSG00000150907**	**FOXO1**	**3453**	**0.542957**	**0.000456**
ENSG00000105173	CCNE1	278.2	-0.77502	0.000498
ENSG00000100889	PCK2	625.3	-0.58965	0.001163
ENSG00000033327	GAB2	4616.3	0.539883	0.001197
ENSG00000005884	ITGA3	428.5	-0.63642	0.001649
ENSG00000143384	MCL1	8554.3	-0.38992	0.003391
ENSG00000132155	RAF1	5603.9	0.235334	0.006157
ENSG00000184481	FOXO4	614.5	0.290364	0.006424
ENSG00000177455	CD19	10126.7	-0.47905	0.008917
ENSG00000123374	CDK2	445.8	0.424963	0.009086
ENSG00000096384	HSP90AB1	36356.1	-0.42299	0.018562
ENSG00000077782	FGFR1	535.2	-0.6703	0.021407
ENSG00000186350	RXRA	2001.6	0.609032	0.025436
ENSG00000139626	ITGB7	5095.8	-0.4401	0.026024
ENSG00000137713	PPP2R1B	1246.5	0.225718	0.029029
**ENSG00000164327**	**RICTOR**	**9290.4**	**0.360697**	**0.032784**
ENSG00000168477	TNXB	359.7	0.577742	0.033591
ENSG00000134460	IL2RA	2330.4	0.493945	0.03385
ENSG00000177885	GRB2	9921.5	-0.30021	0.033857
ENSG00000117020	AKT3	1472.6	0.609996	0.035401
ENSG00000177105	RHOG	543	-0.46489	0.037189
ENSG00000165699	TSC1	5566.1	0.269777	0.042556
ENSG00000068971	PPP2R5B	943.3	-0.39999	0.048169

* positive numbers mean a relative upregulation during ibrutinib therapy in vivo

Used gene sets: Reactome_PI3K_AKT_signalling_in_cancer (no. M27162) and KEGG: PI3K-Akt signaling pathway in Homo sapiens (has04151)

Supplemental Table 4: List of genes that are positively regulated by FoxO1 (36) and upregulated after ibrutinib treatment and genes that are negatively regulated by FoxO1 (36) and downregulated after ibrutinib treatment.

Gene ID	Gene name	Base Mean (reads per million)	Fold Change (log2)*	Adjusted P-value
ENSG00000113369	ARRDC3	11321.5	0.602083	0.00012
ENSG00000168026	TTC21A	3640.6	0.655718	0.000171
ENSG00000112182	BACH2	7294.8	0.569345	0.000267
ENSG00000196712	NF1	7960.4	0.535228	0.000286
ENSG00000150907	FOXO1	3453	0.542957	0.000456
ENSG00000197044	ZNF441	2237	0.665786	0.000672
ENSG00000134245	WNT2B	333.6	0.792452	0.000904
ENSG00000164754	RAD21	7400.1	0.301939	0.001184
ENSG00000100784	RPS6KA5	1372.8	0.451861	0.001704
ENSG00000083896	YTHDC1	9892	0.367052	0.002776
ENSG00000125827	TMX4	2988.3	0.428564	0.002878
ENSG00000133030	MPRIP	5002	0.412277	0.003741
ENSG00000213923	CSNK1E	4612.6	0.541285	0.004421
ENSG00000102081	FMR1	7606.5	0.285384	0.00479
ENSG00000168944	CEP120	3187.9	0.314058	0.006817
ENSG00000145687	SSBP2	1651.8	0.401934	0.006836
ENSG00000163346	PBXIP1	6916.4	0.517252	0.007653
ENSG00000100629	CEP128	475.9	0.546792	0.009957
ENSG00000144645	OSBPL10	6584.8	0.504423	0.012967
ENSG00000214413	BBIP1	719.8	0.390755	0.014438
ENSG00000198408	MGEA5	26595	0.202157	0.016265
ENSG00000135093	USP30	639.8	0.42831	0.018571
ENSG00000080839	RBL1	1352.9	0.33874	0.019494
ENSG00000184226	PCDH9	8123.5	0.634127	0.023167
ENSG00000165029	ABCA1	10492.4	0.581847	0.023203
ENSG00000175727	MLXIP	2786.7	0.348421	0.023657
ENSG00000004399	PLXND1	1851.8	0.491155	0.025615
ENSG00000164327	RICTOR	9290.4	0.360697	0.032784
ENSG00000083168	KAT6A	10727.1	0.490613	0.036279
ENSG00000076685	NT5C2	4787.5	0.26132	0.037234
ENSG00000167703	SLC43A2	1994.7	0.394992	0.038795
ENSG00000174469	CNTNAP2	449.5	0.648414	0.038996
ENSG00000115464	USP34	33103.1	0.352577	0.039517
ENSG00000109790	KLHL5	3082.2	0.424135	0.042084
ENSG00000132842	AP3B1	9381.7	0.280561	0.042446
ENSG00000165699	TSC1	5566.1	0.269777	0.042556
ENSG00000134109	EDEM1	8386.2	0.403641	0.042641
ENSG00000104375	STK3	185.8	0.457383	0.045992
ENSG00000198625	MDM4	12785.6	0.300532	0.047206
ENSG00000104064	GABPB1	3217.5	0.281621	0.0495
ENSG00000156738	MS4A1	11298.8	-1.01789	7.28E-06

ENSG00000168734	PKIG	1028.3	-0.87818	1.04E-05
ENSG00000103653	CSK	6537.2	-0.54409	0.000144
ENSG00000130429	ARPC1B	2532.3	-0.80892	0.000172
ENSG00000162144	CYB561A3	5319.5	-0.68861	0.000227
ENSG00000085117	CD82	2296.9	-0.56	0.001062
ENSG00000051108	HERPUD1	5129.5	-0.4679	0.003264
ENSG00000028277	POU2F2	3874.9	-0.43416	0.006532
ENSG00000065485	PDIA5	146.4	-0.5178	0.006575
ENSG00000197157	SND1	8736	-0.23763	0.006775
ENSG00000137101	CD72	1913.9	-0.60838	0.007259
ENSG00000177455	CD19	10126.7	-0.47905	0.008917
ENSG00000115919	KYNU	2299.7	-0.42295	0.009957
ENSG00000162631	NTNG1	167.2	-0.74183	0.012596
ENSG00000137571	SLCO5A1	200.1	-0.73844	0.012617
ENSG00000172292	CERS6	485.9	-0.61863	0.018229
ENSG00000197629	MPEG1	1924.9	-0.60204	0.023444
ENSG00000141337	ARSG	288.1	-0.59263	0.024351
ENSG00000168275	COA6	127.5	-0.43628	0.025451
ENSG00000107968	MAP3K8	1284.4	-0.42511	0.027211
ENSG00000158481	CD1C	103.5	-0.64755	0.031502
ENSG00000117676	RPS6KA1	2705.1	-0.31708	0.033419
ENSG00000177885	GRB2	9921.5	-0.30021	0.033857
ENSG00000054611	TBC1D22A	5574.7	-0.26855	0.033975
* positive numbers mean a relative upregulation during ibrutinib therapy in vivo				

Supplemental Table 5: List of top differentially expressed genes during idelalisib therapy in vivo (top 250 upregulated and top 250 downregulated mRNAs)

Gene ID	Gene name	Base Mean (reads per million)	Fold Change (log2)*	Adjusted P-value
ENSG00000150672	DLG2	769.1	1.264081	2.74E-26
ENSG00000179088	C12orf42	445.4	1.185332	1.99E-12
ENSG00000204003	AL355987.1	154.5	1.059731	2.20E-06
ENSG00000214140	PRCD	302.1	1.051967	3.37E-08
ENSG00000102053	ZC3H12B	807.5	1.034461	7.18E-15
ENSG00000099958	DERL3	1859.1	1.028104	6.48E-10
ENSG00000213923	CSNK1E	8470.1	1.026098	1.73E-12
ENSG00000169762	TAPT1	2653.3	0.982449	1.65E-17
ENSG00000172794	RAB37	2525.7	0.97666	4.68E-10
ENSG00000226742	HSBP1L1	204.4	0.950384	1.89E-08
ENSG00000186350	RXRA	3513.3	0.948381	1.12E-06
ENSG00000135116	HRK	2536.5	0.928979	6.06E-06
ENSG00000196814	MVB12B	223.0	0.897331	3.36E-06
ENSG00000108551	RASD1	168.1	0.89666	3.81E-05
ENSG00000161036	LRWD1	4437.9	0.893363	8.81E-14
ENSG00000121966	CXCR4	62466.9	0.892807	9.29E-16
ENSG00000007516	BAIAP3	7823.0	0.886991	1.72E-09
ENSG00000120896	SORBS3	1182.3	0.880919	1.68E-12
ENSG00000128596	CCDC136	110.9	0.874584	6.03E-08
ENSG00000159788	RGS12	184.1	0.848664	1.34E-05
ENSG00000185022	MAFF	201.9	0.810917	9.23E-05
ENSG00000124074	ENKD1	213.1	0.806044	4.50E-05
ENSG00000213231	TCL1B	310.6	0.787017	8.21E-06
ENSG00000169435	RASSF6	241.5	0.785385	0.000636
ENSG00000174469	CNTNAP2	1772.2	0.785148	5.00E-05
ENSG00000099953	MMP11	849.6	0.783706	2.63E-05
ENSG00000010278	CD9	173.6	0.779133	0.000165
ENSG00000187922	LCN10	1571.6	0.778609	0.004124
ENSG00000166348	USP54	436.1	0.770367	1.37E-07
ENSG00000182871	COL18A1	7425.0	0.766373	0.005436
ENSG00000132003	ZSWIM4	237.3	0.761512	1.19E-06
ENSG00000055118	KCNH2	1715.3	0.759768	2.92E-10
ENSG00000139112	GABARAPL1	397.7	0.755038	1.48E-07
ENSG00000140807	NKD1	162.1	0.754672	7.12E-05
ENSG00000118432	CNR1	1558.3	0.749754	0.00083
ENSG00000095397	WHRN	5399.2	0.744095	9.88E-07
ENSG00000088256	GNA11	200.4	0.740503	8.74E-05
ENSG00000204580	DDR1	2066.2	0.738208	4.39E-06
ENSG00000158106	RHPN1	587.1	0.734025	2.48E-06
ENSG00000204001	LCN8	1360.7	0.728148	0.000356
ENSG00000198885	ITPRIPL1	432.5	0.724125	0.000244
ENSG00000188026	RILPL1	189.0	0.721395	8.70E-07
ENSG00000174175	SELP	1297.6	0.714987	2.77E-06
ENSG00000179859	AC104581.1	240.8	0.711447	5.37E-07
ENSG00000002587	HS3ST1	1038.7	0.709852	0.000277
ENSG00000119900	OGFRL1	1668.2	0.70643	2.78E-10
ENSG00000130787	HIP1R	3155.1	0.705077	7.54E-06
ENSG00000110944	IL23A	193.9	0.70169	5.70E-05
ENSG00000135299	ANKRD6	324.8	0.699331	8.69E-06
ENSG00000188747	NOXA1	106.9	0.698524	1.53E-05
ENSG00000105948	TTC26	223.5	0.697349	7.42E-06
ENSG00000196712	NF1	2244.9	0.696343	7.02E-13
ENSG00000112182	BACH2	12531.8	0.693618	1.99E-12
ENSG00000171246	NPTX1	2073.7	0.689928	7.14E-06
ENSG00000100490	CDKL1	1068.2	0.688818	0.000143

ENSG00000183397	C19orf71	166.3	0.681669	5.51E-05
ENSG00000204262	COL5A2	259.6	0.675584	0.000697
ENSG00000159388	BTG2	20252.0	0.675199	4.29E-08
ENSG00000126351	THRA	2154.5	0.671959	1.60E-05
ENSG00000103253	HAGHL	456.4	0.670425	0.000593
ENSG00000142675	CNKSR1	102.4	0.662638	0.000801
ENSG00000272398	CD24	11489.4	0.660637	0.001764
ENSG00000081059	TCF7	1866.8	0.659158	7.37E-05
ENSG00000102870	ZNF629	267.6	0.649431	0.000276
ENSG00000111666	CHPT1	1986.1	0.648648	2.24E-16
ENSG00000173530	TNFRSF10D	782.0	0.647348	1.27E-05
ENSG00000049246	PER3	262.9	0.64729	0.000283
ENSG00000157216	SSBP3	539.8	0.646527	3.53E-05
ENSG00000185112	FAM43A	127.2	0.646034	0.006203
ENSG00000184897	H1FX	7381.8	0.644537	0.001708
ENSG00000060138	YBX3	23013.6	0.643215	5.64E-06
ENSG00000185950	IRS2	989.5	0.642416	6.35E-05
ENSG00000136161	RCBTB2	235.0	0.639198	0.000189
ENSG00000120129	DUSP1	16642.5	0.638884	6.18E-05
ENSG00000071051	NCK2	4007.4	0.637784	1.26E-06
ENSG00000198832	SELENOM	388.1	0.632728	3.51E-05
ENSG00000161249	DMKN	373.0	0.631	0.000596
ENSG00000132510	KDM6B	2378.4	0.623355	5.17E-07
ENSG00000178075	GRAMD1C	315.8	0.622838	0.007034
ENSG00000066923	STAG3	664.9	0.620669	5.22E-07
ENSG00000198324	FAM109A	219.7	0.619234	0.002089
ENSG00000262621	AC025283.2	162.6	0.6162	0.000221
ENSG00000166707	ZCCHC18	2168.4	0.615462	0.000188
ENSG00000273820	USP27X	229.5	0.614062	0.001775
ENSG00000104064	GABPB1	3426.9	0.613887	3.79E-20
ENSG00000117318	ID3	2528.1	0.613881	0.000107
ENSG00000198932	GPRASP1	4427.0	0.611997	9.48E-10
ENSG00000111266	DUSP16	168.1	0.61165	0.002757
ENSG00000175727	MLXIP	5277.0	0.611606	1.26E-06
ENSG00000152495	CAMK4	313.9	0.611413	0.027955
ENSG00000172071	EIF2AK3	1925.9	0.609746	5.79E-07
ENSG00000172780	RAB43	2869.9	0.605911	5.03E-07
ENSG00000134294	SLC38A2	9318.7	0.60263	2.35E-09
ENSG00000173825	TIGD3	115.3	0.597147	0.000336
ENSG00000256574	OR13A1	716.1	0.596684	3.56E-07
ENSG00000162591	MEGF6	5220.6	0.592172	2.79E-08
ENSG00000084693	AGBL5	733.5	0.588711	3.23E-06
ENSG00000166532	RIMKLB	1627.1	0.587423	2.20E-06
ENSG00000103066	PLA2G15	1026.4	0.586378	4.88E-07
ENSG00000141682	PMAIP1	6367.2	0.584374	2.54E-09
ENSG00000072364	AFF4	10437.1	0.583801	3.82E-16
ENSG00000100629	CEP128	218.4	0.582022	7.96E-07
ENSG00000278535	DHRS11	184.2	0.581762	0.000422
ENSG00000168675	LDLRAD4	1057.8	0.581216	1.67E-06
ENSG00000145451	GLRA3	147.5	0.578777	0.003927
ENSG00000254870	ATP6V1G2-DDX39B	298.7	0.577889	0.005791
ENSG00000165115	KIF27	407.0	0.569002	9.93E-09
ENSG00000139946	PELI2	629.1	0.568391	0.000297
ENSG00000112139	MDGA1	1656.1	0.567867	0.001299
ENSG00000164442	CITED2	386.9	0.567848	0.006538
ENSG00000131941	RHPN2	395.9	0.564424	0.003937
ENSG00000108984	MAP2K6	1797.0	0.564284	2.42E-05
ENSG00000004399	PLXND1	3685.6	0.562241	2.03E-06
ENSG00000099860	GADD45B	1986.8	0.560005	0.013255
ENSG00000163001	CFAP36	468.2	0.557512	6.56E-06

ENSG00000122970	IFT81	119.1	0.556875	0.010623
ENSG00000198369	SPRED2	185.1	0.556293	0.010899
ENSG00000011275	RNF216	7274.8	0.555379	3.52E-08
ENSG00000141506	PIK3R5	784.7	0.554884	0.000441
ENSG00000141013	GAS8	228.4	0.553939	0.000209
ENSG00000119669	IRF2BPL	1084.9	0.553873	9.12E-06
ENSG00000101190	TCFL5	591.2	0.553024	0.000588
ENSG00000132205	EMILIN2	490.9	0.552138	0.001557
ENSG00000196843	ARID5A	1189.1	0.549857	0.00084
ENSG00000259384	GH1	123.6	0.54956	0.008298
ENSG00000118263	KLF7	2059.7	0.549276	0.002367
ENSG00000164300	SERINC5	4155.4	0.548144	1.13E-05
ENSG00000116717	GADD45A	233.9	0.548081	0.003264
ENSG00000007314	SCN4A	442.9	0.541399	5.49E-05
ENSG00000152518	ZFP36L2	8927.6	0.540895	1.93E-08
ENSG00000117519	CNN3	161.6	0.54063	0.010668
ENSG00000101605	MYOM1	626.1	0.538838	1.17E-05
ENSG00000198879	SFMBT2	1160.3	0.538161	0.000534
ENSG00000187824	TMEM220	158.4	0.536399	0.00548
ENSG00000164136	IL15	403.4	0.535979	0.00268
ENSG00000196476	C20orf96	322.7	0.533349	0.006158
ENSG00000176714	CCDC121	113.8	0.531971	0.020243
ENSG00000164938	TP53INP1	6538.3	0.531357	0.02476
ENSG00000168026	TTC21A	4283.3	0.530847	1.56E-11
ENSG00000104081	BMF	19738.4	0.530439	3.37E-08
ENSG00000182022	CHST15	7986.3	0.528256	1.76E-06
ENSG00000131042	LILRB2	1427.3	0.528106	2.53E-07
ENSG00000162063	CCNF	147.4	0.527767	0.000797
ENSG00000125844	RRBP1	6840.6	0.525922	0.006079
ENSG00000134109	EDEM1	12857.7	0.525088	4.48E-12
ENSG00000169962	TAS1R3	331.4	0.523804	0.012107
ENSG00000107902	LHPP	1652.6	0.52136	0.043163
ENSG00000126091	ST3GAL3	423.4	0.520776	1.40E-06
ENSG00000127528	KLF2	4505.1	0.518945	0.000546
ENSG00000159433	STARD9	4440.5	0.518695	0.000127
ENSG00000198467	TPM2	259.2	0.518147	0.003997
ENSG00000120832	MTERF2	563.2	0.517093	0.003927
ENSG00000196584	XRCC2	106.4	0.51582	0.016507
ENSG00000104866	PPP1R37	1206.5	0.514182	0.000172
ENSG00000145990	GFOD1	4841.9	0.514161	6.82E-06
ENSG00000165113	GKAP1	338.8	0.513645	0.000475
ENSG00000170915	PAQR8	796.7	0.512645	0.013945
ENSG00000152465	NMT2	271.6	0.512163	2.13E-05
ENSG00000133302	SLF1	451.9	0.511666	0.000638
ENSG00000109787	KLF3	929.0	0.511655	0.00136
ENSG00000196275	GTF2IRD2	1706.9	0.510052	7.14E-11
ENSG00000156313	RPGR	564.0	0.509769	5.29E-05
ENSG00000131375	CAPN7	2474.6	0.509167	1.18E-05
ENSG00000125740	FOSB	24529.8	0.507647	0.016365
ENSG00000182324	KCNJ14	117.0	0.507273	0.000634
ENSG00000138606	SHF	124.1	0.507	0.013488
ENSG00000171223	JUNB	10594.2	0.5057	0.023617
ENSG00000255150	EID3	539.6	0.504443	0.000301
ENSG00000142188	TMEM50B	2192.2	0.503791	9.78E-10
ENSG00000111886	GABRR2	206.9	0.50234	0.000741
ENSG00000189057	FAM111B	1802.4	0.501564	0.031736
ENSG00000128011	LRFN1	113.4	0.501298	0.011449
ENSG00000157514	TSC22D3	45655.7	0.499516	0.002299
ENSG00000126368	NR1D1	370.5	0.499498	0.008351
ENSG00000005206	SPPL2B	3205.9	0.499387	6.94E-05

ENSG00000258539	AC068896.1	287.8	0.497951	0.002073
ENSG00000167393	PPP2R3B	1287.3	0.497228	0.000613
ENSG00000124126	PREX1	10888.3	0.496943	2.28E-05
ENSG00000001167	NFYA	2961.1	0.496843	3.19E-06
ENSG00000184271	POU6F1	1323.8	0.496509	7.22E-08
ENSG00000196172	ZNF681	484.9	0.495694	0.016036
ENSG00000221968	FADS3	2652.9	0.494424	9.24E-06
ENSG00000160961	ZNF333	1539.8	0.493527	4.13E-06
ENSG00000039523	RIPOR1	9765.4	0.491621	1.32E-06
ENSG00000246922	UBAP1L	1346.1	0.490126	9.51E-05
ENSG00000170345	FOS	15965.3	0.488643	0.004355
ENSG00000100219	XBP1	2036.0	0.488479	7.97E-05
ENSG00000133878	DUSP26	202.5	0.485069	0.004421
ENSG00000106459	NRF1	1288.3	0.484947	2.19E-09
ENSG00000108557	RAI1	1595.4	0.484855	0.002929
ENSG00000173950	XXYLT1	209.3	0.482887	0.000368
ENSG00000163346	PBXIP1	8421.0	0.482036	0.00015
ENSG00000143322	ABL2	1882.0	0.480599	3.07E-18
ENSG00000131153	GINS2	101.2	0.479319	0.015811
ENSG00000106537	TSPAN13	343.5	0.478074	0.005689
ENSG00000106624	AEBP1	4914.0	0.477044	0.001114
ENSG00000079337	RAPGEF3	9881.2	0.474986	0.001324
ENSG00000083812	ZNF324	635.2	0.474743	0.000291
ENSG00000132326	PER2	659.2	0.474649	0.000653
ENSG00000111752	PHC1	4489.6	0.473787	1.43E-05
ENSG00000163535	SGO2	133.9	0.472138	0.022691
ENSG00000169105	CHST14	636.7	0.471184	0.004331
ENSG00000173334	TRIB1	2058.7	0.47104	0.028753
ENSG00000165029	ABCA1	2139.4	0.470323	0.002083
ENSG00000213015	ZNF580	1261.3	0.469531	2.74E-06
ENSG00000150347	ARID5B	9318.3	0.468927	0.000499
ENSG00000204569	PPP1R10	3316.4	0.468535	0.001818
ENSG00000110987	BCL7A	1357.2	0.466932	0.007058
ENSG00000115109	EPB41L5	597.1	0.466734	0.005222
ENSG00000196689	TRPV1	598.8	0.466718	0.000565
ENSG00000166501	PRKCB	45216.1	0.466176	9.41E-07
ENSG00000174483	BBS1	633.7	0.465621	0.0002
ENSG00000206530	CFAP44	2376.8	0.465528	6.64E-06
ENSG00000160223	ICOSLG	5142.0	0.465501	0.000144
ENSG00000177606	JUN	41371.4	0.465068	0.044188
ENSG00000139746	RBM26	8840.2	0.463598	8.53E-09
ENSG00000143669	LYST	10772.8	0.461937	7.83E-06
ENSG00000144445	KANSL1L	915.7	0.461704	9.52E-08
ENSG00000123374	CDK2	999.4	0.458141	6.35E-05
ENSG00000119138	KLF9	3322.0	0.457189	0.005829
ENSG00000146856	AGBL3	122.5	0.456664	0.021922
ENSG00000160991	ORAI2	17975.7	0.455049	0.000136
ENSG00000204388	HSPA1B	1235.2	0.454919	0.002702
ENSG00000174428	GTF2IRD2B	3156.0	0.454321	7.23E-14
ENSG00000112144	ICK	1171.1	0.454097	0.000257
ENSG00000163611	SPICE1	1690.6	0.453617	5.40E-07
ENSG00000140386	SCAPER	1223.2	0.452598	6.51E-06
ENSG00000123411	IKZF4	333.7	0.450826	0.00069
ENSG00000215912	TTC34	307.7	0.450401	0.010764
ENSG00000121879	PIK3CA	3075.9	0.449979	3.52E-06
ENSG00000136158	SPRY2	212.7	0.44964	0.03528
ENSG00000239521	GATS	1218.7	0.449615	1.18E-05
ENSG00000247315	ZCCHC3	1531.4	0.448425	5.31E-06
ENSG00000160209	PDXK	1071.5	0.447983	0.000335
ENSG00000133612	AGAP3	1113.0	0.447288	0.00025

ENSG00000157796	WDR19	3796.3	0.447116	8.48E-06
ENSG00000165507	C10orf10	603.2	0.446465	4.20E-05
ENSG00000141665	FBXO15	103.8	0.446048	0.017762
ENSG00000141425	RPRD1A	1514.1	0.445037	1.70E-06
ENSG00000135108	FBXO21	2323.9	0.444675	5.98E-05
ENSG00000133030	MPRIP	8284.5	0.444543	0.000318
ENSG00000135924	DNAJB2	5086.1	0.443562	4.45E-10
ENSG00000132842	AP3B1	3593.0	0.442942	9.82E-05
ENSG00000130304	SLC27A1	340.7	0.441619	0.013809
ENSG00000136237	RAPGEF5	585.6	0.440425	0.003621
ENSG00000122126	OCRL	937.8	0.440409	1.21E-05
ENSG00000242689	CNTF	121.4	0.440241	0.003265
ENSG00000119280	C1orf198	149.7	0.440071	0.014846
ENSG00000162430	SELENON	1470.9	0.439132	0.000406
ENSG00000164691	TAGAP	16638.2	0.437756	6.22E-05
ENSG00000110448	CD5	3562.7	-0.57179	1.82E-09
ENSG00000104774	MAN2B1	5199.2	-0.57241	2.18E-06
ENSG00000010219	DYRK4	974.4	-0.57326	1.05E-07
ENSG00000198648	STK39	194.7	-0.57487	0.001436
ENSG00000104998	IL27RA	1377.0	-0.57539	6.78E-05
ENSG00000117411	B4GALT2	212.8	-0.57551	2.86E-05
ENSG00000244509	APOBEC3C	3975.2	-0.5759	5.43E-11
ENSG00000196296	ATP2A1	174.5	-0.57749	0.004232
ENSG00000077549	CAPZB	2161.9	-0.57812	1.02E-10
ENSG00000164885	CDK5	150.0	-0.57826	0.000317
ENSG00000197879	MYO1C	2447.1	-0.57885	0.007243
ENSG00000145088	EAF2	1264.9	-0.57923	0.000402
ENSG00000136717	BIN1	2057.2	-0.57996	1.41E-07
ENSG00000104921	FCER2	20132.9	-0.57999	1.58E-06
ENSG00000173531	MST1	383.1	-0.58128	0.000101
ENSG00000126264	HCST	640.0	-0.58147	2.40E-06
ENSG00000112137	PHACTR1	908.8	-0.58237	0.00061
ENSG00000155366	RHOC	2003.9	-0.58606	9.68E-05
ENSG00000076604	TRAF4	3512.1	-0.58608	0.003023
ENSG00000023191	RNH1	3987.3	-0.59136	1.99E-12
ENSG00000110002	VWA5A	389.2	-0.59251	8.02E-06
ENSG00000168878	SFTPB	1729.5	-0.59276	3.40E-05
ENSG00000111679	PTPN6	15879.0	-0.59468	2.15E-06
ENSG00000256269	HMBS	431.5	-0.59702	1.95E-07
ENSG00000173621	LRFN4	418.7	-0.59792	0.001331
ENSG00000049192	ADAMTS6	1589.7	-0.59874	2.38E-06
ENSG00000103415	HMOX2	841.3	-0.60113	1.24E-12
ENSG00000125508	SRMS	101.4	-0.60199	0.000187
ENSG00000115919	KYNU	910.3	-0.60399	1.00E-07
ENSG00000101082	SLA2	166.9	-0.60498	0.000567
ENSG00000137936	BCAR3	738.3	-0.60696	8.09E-07
ENSG00000154518	ATP5G3	1393.1	-0.60835	1.72E-08
ENSG00000087266	SH3BP2	6317.9	-0.61238	3.16E-06
ENSG00000166886	NAB2	702.9	-0.61345	0.000258
ENSG00000163808	KIF15	123.6	-0.61366	0.002474
ENSG00000101213	PTK6	131.8	-0.61367	0.00133
ENSG00000170667	RASA4B	366.7	-0.61375	7.39E-06
ENSG00000122862	SRGN	1966.9	-0.61423	5.94E-06
ENSG00000101336	HCK	775.8	-0.61483	0.000745
ENSG00000182866	LCK	3171.9	-0.6158	3.63E-08
ENSG00000136960	ENPP2	446.0	-0.61683	5.94E-05
ENSG00000151413	NUBPL	361.3	-0.61707	1.29E-06
ENSG00000146094	DOK3	2404.5	-0.61748	5.38E-07
ENSG00000125434	SLC25A35	437.9	-0.61996	1.35E-06
ENSG00000111640	GAPDH	16329.2	-0.62008	2.59E-12

ENSG00000197043	ANXA6	8322.0	-0.62071	9.33E-10
ENSG00000103187	COTL1	7068.7	-0.62119	9.57E-09
ENSG00000183765	CHEK2	418.9	-0.62325	1.21E-08
ENSG00000122873	CISD1	116.4	-0.62364	0.000467
ENSG00000137959	IFI44L	1237.5	-0.62492	0.031814
ENSG00000111639	MRPL51	680.1	-0.62522	5.92E-08
ENSG00000111816	FRK	131.6	-0.62605	0.002536
ENSG00000112759	SLC29A1	197.6	-0.62706	0.000148
ENSG00000163344	PMVK	461.9	-0.62962	8.13E-15
ENSG00000173208	ABCD2	311.2	-0.63041	1.34E-05
ENSG00000182379	NXPH4	114.2	-0.63214	0.000699
ENSG00000198513	ATL1	144.2	-0.63282	0.000654
ENSG00000126246	IGFLR1	3483.0	-0.63324	1.24E-07
ENSG00000101298	SNPH	132.7	-0.63356	0.000667
ENSG00000163932	PRKCD	2016.2	-0.63426	4.00E-10
ENSG00000111696	NT5DC3	1112.5	-0.63443	5.64E-06
ENSG00000103316	CRYM	112.5	-0.63706	0.00568
ENSG00000062282	DGAT2	207.8	-0.63946	4.91E-05
ENSG00000172575	RASGRP1	592.9	-0.64093	0.001207
ENSG00000157593	SLC35B2	2070.7	-0.64119	1.23E-07
ENSG00000129467	ADCY4	206.6	-0.64181	0.0001
ENSG00000163637	PRICKLE2	142.6	-0.64268	0.005717
ENSG00000173898	SPTBN2	733.1	-0.64286	0.000704
ENSG00000164674	SYTL3	199.2	-0.64364	4.12E-06
ENSG00000125454	SLC25A19	1069.0	-0.64381	9.06E-07
ENSG00000119411	BSPRY	300.5	-0.64406	7.79E-06
ENSG00000152782	PANK1	182.1	-0.64656	0.000336
ENSG00000128604	IRF5	2928.3	-0.64948	1.50E-13
ENSG00000175928	LRRN1	858.9	-0.6508	2.85E-06
ENSG00000048462	TNFRSF17	124.9	-0.65101	0.000982
ENSG00000171314	PGAM1	2966.4	-0.65173	3.44E-10
ENSG00000129595	EPB41L4A	154.9	-0.65186	0.004919
ENSG00000196189	SEMA4A	2240.9	-0.65261	2.40E-06
ENSG00000198720	ANKRD13B	208.4	-0.65273	0.007364
ENSG00000185760	KCNQ5	490.9	-0.65328	0.000433
ENSG00000132185	FCRLA	10220.2	-0.65624	1.16E-08
ENSG00000184924	PTRHD1	451.4	-0.65654	4.51E-08
ENSG00000102174	PHEX	1518.5	-0.65664	8.64E-07
ENSG00000198178	CLEC4C	184.1	-0.65821	0.00111
ENSG00000132718	SYT11	508.2	-0.66007	0.00019
ENSG00000111328	CDK2AP1	308.0	-0.66179	6.10E-05
ENSG00000250151	ARPC4-TTLL3	596.6	-0.6619	7.52E-10
ENSG00000239672	NME1	430.3	-0.66277	7.54E-06
ENSG00000120708	TGFBI	6429.0	-0.66302	0.004192
ENSG00000160856	FCRL3	19465.1	-0.66474	0.00226
ENSG00000016391	CHDH	2154.4	-0.66489	0.000462
ENSG00000122694	GLIPR2	438.3	-0.66722	4.43E-09
ENSG00000105173	CCNE1	221.4	-0.6688	0.000966
ENSG00000139626	ITGB7	7507.1	-0.66886	9.34E-08
ENSG00000139880	CDH24	276.6	-0.66926	6.57E-06
ENSG00000102575	ACP5	2508.3	-0.67043	1.45E-07
ENSG00000159399	HK2	377.7	-0.67158	6.22E-05
ENSG00000167483	FAM129C	18097.2	-0.67167	1.13E-06
ENSG00000151689	INPP1	279.9	-0.67205	1.11E-08
ENSG00000267228	AC012254.2	200.5	-0.67599	2.21E-05
ENSG00000166839	ANKDD1A	153.6	-0.67635	0.000168
ENSG00000013016	EHD3	2050.2	-0.6764	6.32E-14
ENSG00000188322	SBK1	180.7	-0.67728	0.000631
ENSG00000160446	ZDHHC12	363.6	-0.67909	4.21E-07
ENSG00000188130	MAPK12	228.7	-0.68295	0.001522

ENSG00000163406	SLC15A2	1553.5	-0.68613	3.58E-05
ENSG00000140451	PIF1	128.2	-0.68758	4.32E-05
ENSG00000100889	PCK2	1229.8	-0.68985	5.09E-10
ENSG00000173559	NABP1	2005.5	-0.69392	5.25E-05
ENSG00000172824	CES4A	365.1	-0.69482	0.000321
ENSG00000175489	LRRC25	227.5	-0.69742	0.000115
ENSG00000151490	PTPRO	1916.0	-0.69861	5.73E-06
ENSG00000101265	RASSF2	4909.3	-0.69926	4.16E-09
ENSG00000118640	VAMP8	1827.5	-0.70346	1.48E-12
ENSG00000185669	SNAI3	106.6	-0.70358	0.000208
ENSG00000177106	EPS8L2	379.1	-0.70449	3.63E-11
ENSG00000100097	LGALS1	1450.9	-0.70572	4.55E-08
ENSG00000184009	ACTG1	21558.9	-0.70626	4.04E-12
ENSG00000187994	RINL	1741.8	-0.70654	1.21E-08
ENSG00000075240	GRAMD4	476.5	-0.70894	0.000123
ENSG00000163683	SMIM14	2608.4	-0.71102	1.99E-11
ENSG00000087589	CASS4	452.6	-0.71276	1.56E-11
ENSG00000143549	TPM3	11848.6	-0.7162	5.01E-14
ENSG00000187510	PLEKHG7	125.8	-0.71701	0.001058
ENSG00000130598	TNNI2	192.5	-0.71782	2.50E-07
ENSG00000204839	MROH6	184.7	-0.71806	5.29E-05
ENSG00000198715	GLMP	481.8	-0.71844	1.79E-08
ENSG00000109919	MTCH2	635.4	-0.71886	4.77E-18
ENSG00000182489	XKRX	677.3	-0.72102	3.18E-06
ENSG00000159335	PTMS	324.2	-0.72189	7.78E-05
ENSG00000130429	ARPC1B	7039.6	-0.72606	1.98E-09
ENSG00000167264	DUS2	2766.2	-0.72639	1.24E-07
ENSG00000161243	FBXO27	119.3	-0.72689	0.000839
ENSG00000177697	CD151	342.3	-0.73074	3.90E-06
ENSG00000267120	AD000671.2	438.3	-0.73119	1.80E-09
ENSG00000146386	ABRACL	357.5	-0.73153	1.57E-07
ENSG00000100473	COCH	1569.5	-0.73258	6.68E-05
ENSG00000138031	ADCY3	809.8	-0.73365	1.29E-06
ENSG00000156738	MS4A1	21927.1	-0.73796	0.002089
ENSG00000188677	PARVB	615.5	-0.74081	6.90E-13
ENSG00000169413	RNASE6	891.8	-0.74117	0.000114
ENSG00000179388	EGR3	279.6	-0.74134	0.010696
ENSG00000198520	C1orf228	917.3	-0.7429	1.15E-05
ENSG00000005844	ITGAL	3895.8	-0.74393	1.80E-09
ENSG00000108175	ZMIZ1	313.4	-0.74433	9.21E-06
ENSG00000178537	SLC25A20	263.5	-0.74497	8.15E-06
ENSG00000254415	SIGLEC14	286.1	-0.74578	4.11E-08
ENSG00000123360	PDE1B	729.9	-0.74903	6.06E-06
ENSG00000002745	WNT16	335.6	-0.75136	4.02E-05
ENSG00000105808	RASA4	1531.1	-0.75274	2.42E-05
ENSG00000030582	GRN	6008.5	-0.75631	1.54E-10
ENSG00000050438	SLC4A8	257.5	-0.75936	0.000182
ENSG00000089127	OAS1	2421.5	-0.76141	1.29E-07
ENSG00000100577	GSTZ1	580.9	-0.76224	1.98E-09
ENSG00000115129	TP53I3	702.9	-0.76235	0.00022
ENSG00000273047	AL121845.2	143.6	-0.76266	0.000114
ENSG00000177685	CRACR2B	955.4	-0.76353	3.25E-07
ENSG00000115902	SLC1A4	213.9	-0.76769	5.59E-05
ENSG00000184293	CLECL1	1537.6	-0.76825	2.32E-08
ENSG00000177301	KCNA2	167.8	-0.77062	5.28E-05
ENSG00000196975	ANXA4	414.3	-0.77388	5.73E-06
ENSG00000198176	TFDP1	1385.2	-0.77485	5.35E-07
ENSG00000189159	JPT1	385.1	-0.77487	1.05E-07
ENSG00000277632	CCL3	127.6	-0.77536	0.0005
ENSG00000184060	ADAP2	124.6	-0.77537	0.000302

ENSG00000189233	NUGGC	535.5	-0.77565	5.30E-06
ENSG00000196154	S100A4	2739.5	-0.77616	1.50E-10
ENSG00000107833	NPM3	186.6	-0.77797	5.13E-05
ENSG00000183963	SMTN	211.7	-0.78109	1.05E-05
ENSG00000149577	SIDT2	7723.4	-0.78139	7.07E-07
ENSG00000158406	HIST1H4H	184.8	-0.78378	1.83E-05
ENSG00000139625	MAP3K12	315.5	-0.78507	5.79E-07
ENSG00000263961	C1orf186	1921.6	-0.79285	1.45E-13
ENSG00000162407	PLPP3	150.3	-0.79403	0.000112
ENSG00000166510	CCDC68	110.7	-0.79738	0.000115
ENSG00000239732	TLR9	252.8	-0.80004	3.38E-08
ENSG00000142512	SIGLEC10	2827.0	-0.80161	2.25E-12
ENSG00000268041	AC010616.1	133.4	-0.8027	7.55E-07
ENSG00000137460	FHDC1	424.7	-0.80328	2.74E-10
ENSG00000145703	IQGAP2	425.4	-0.81195	9.86E-07
ENSG00000070614	NDST1	1188.4	-0.81227	8.73E-15
ENSG00000112290	WASF1	277.0	-0.81304	1.80E-09
ENSG00000216490	IFI30	1439.0	-0.81818	1.42E-11
ENSG00000181885	CLDN7	118.6	-0.81849	5.52E-06
ENSG00000158050	DUSP2	1370.2	-0.82005	2.67E-06
ENSG00000187912	CLEC17A	6895.8	-0.82636	6.02E-11
ENSG00000077585	GPR137B	197.5	-0.82689	2.84E-06
ENSG00000229474	PATL2	4409.6	-0.82924	2.81E-08
ENSG00000116701	NCF2	1370.1	-0.83032	1.53E-06
ENSG00000115956	PLEK	3176.6	-0.83213	7.10E-08
ENSG00000074416	MGLL	118.0	-0.83527	1.48E-05
ENSG00000065911	MTHFD2	471.3	-0.8367	6.39E-08
ENSG00000111331	OAS3	1143.7	-0.84136	2.90E-08
ENSG00000166689	PLEKHA7	389.1	-0.84293	3.54E-06
ENSG00000222009	BTBD19	126.6	-0.84661	5.64E-06
ENSG00000149591	TAGLN	2521.1	-0.84865	1.75E-07
ENSG00000173366	AC097637.1	373.6	-0.85104	5.84E-08
ENSG00000137101	CD72	2873.5	-0.85365	5.64E-06
ENSG00000083454	P2RX5	2818.7	-0.85474	4.20E-07
ENSG00000196743	GM2A	3341.9	-0.85588	8.73E-15
ENSG00000160712	IL6R	1129.9	-0.85642	7.17E-08
ENSG00000162144	CYB561A3	17336.1	-0.85899	2.67E-10
ENSG00000134955	SLC37A2	283.4	-0.8595	4.88E-07
ENSG00000188191	PRKAR1B	208.3	-0.86171	6.16E-10
ENSG00000042753	AP2S1	640.4	-0.86594	9.78E-24
ENSG00000146215	CRIP3	522.1	-0.86742	4.00E-10
ENSG00000163687	DNASE1L3	146.6	-0.87189	3.36E-05
ENSG00000151651	ADAM8	2642.6	-0.8729	2.84E-09
ENSG00000042493	CAPG	2733.3	-0.87393	3.52E-08
ENSG00000203896	LIME1	309.1	-0.878	7.53E-11
ENSG00000124256	ZBP1	1317.6	-0.89724	1.88E-07
ENSG00000239961	LILRA4	2422.5	-0.89758	0.000109
ENSG00000155846	PPARGC1B	618.6	-0.89819	1.57E-07
ENSG00000155367	PPM1J	442.3	-0.90049	3.54E-06
ENSG00000105492	SIGLEC6	280.4	-0.90163	6.21E-06
ENSG00000142765	SYTL1	6343.6	-0.90363	7.14E-11
ENSG00000162896	PIGR	2648.3	-0.90923	1.08E-05
ENSG00000188389	PDCD1	243.5	-0.91553	3.98E-07
ENSG00000257950	P2RX5-TAX1BP3	117.2	-0.91961	6.15E-07
ENSG00000116771	AGMAT	274.6	-0.91978	1.82E-08
ENSG00000197629	MPEG1	3710.1	-0.9255	1.69E-06
ENSG00000142798	HSPG2	628.7	-0.929	1.69E-07
ENSG00000128340	RAC2	11122.0	-0.93041	6.15E-14
ENSG00000146192	FGD2	6614.3	-0.93591	5.71E-12
ENSG00000141337	ARSG	437.4	-0.9372	7.37E-07

ENSG00000183091	NEB	994.8	-0.94189	2.94E-07
ENSG00000278195	SSTR3	579.9	-0.944	4.13E-06
ENSG00000106868	SUSD1	608.8	-0.94422	4.25E-08
ENSG00000204983	PRSS1	115.4	-0.94544	4.96E-05
ENSG00000108797	CNTNAP1	2209.3	-0.9472	1.10E-09
ENSG00000174123	TLR10	1390.4	-0.9479	1.56E-05
ENSG00000175274	TP53I11	2491.0	-0.95085	8.49E-10
ENSG00000255274	TMPRSS4-AS1	132.0	-0.97155	5.67E-06
ENSG00000124772	CPNE5	3000.0	-0.97386	6.85E-07
ENSG00000133067	LGR6	194.9	-0.98899	1.60E-08
ENSG00000138623	SEMA7A	796.1	-1.01141	7.16E-15
ENSG00000140678	ITGAX	6514.0	-1.02185	1.28E-10
ENSG00000168081	PNOC	1473.0	-1.03917	6.92E-13
ENSG00000179841	AKAP5	191.3	-1.04161	5.10E-07
ENSG00000103089	FA2H	156.6	-1.04236	1.42E-09
ENSG00000129226	CD68	755.8	-1.05114	2.30E-10
ENSG00000158481	CD1C	139.9	-1.05848	6.00E-07
ENSG00000064886	CHI3L2	642.3	-1.0748	1.80E-07
ENSG00000011028	MRC2	209.3	-1.07719	1.78E-07
ENSG00000204396	VWA7	130.5	-1.09495	1.45E-07
ENSG00000166428	PLD4	128.8	-1.11949	1.03E-07
ENSG00000172771	EFCAB12	261.5	-1.12059	1.38E-09
ENSG00000011590	ZBTB32	2725.1	-1.16681	6.32E-14
ENSG00000186810	CXCR3	489.5	-1.44369	7.82E-17
ENSG00000186818	LILRB4	700.2	-1.72761	6.21E-27

***250 most up-regulated genes (positive fold change) and 250 most down-regulated genes (negative fold change) during idelalisib therapy in vivo

Supplemental Table 6: GO term analysis of differentially expressed genes during idelalisib in vivo therapy (top 30 processes)

GO term*	Description	P-value	FDR q-value	Enrichment	N	B	n	b
GO:0002376	immune system process	3.34E-15	3.12E-11	1.5	2755	447	997	243
GO:0002252	immune effector process	8.35E-14	3.90E-10	1.73	2755	233	934	137
GO:0055086	nucleobase-containing small molecule metabolic process	2.20E-12	6.86E-09	1.93	2755	127	944	84
GO:0006955	immune response	3.03E-12	7.07E-09	2.19	2755	186	527	78
GO:0006887	exocytosis	1.16E-11	2.17E-08	1.78	2755	171	934	103
GO:0044281	small molecule metabolic process	1.17E-11	1.82E-08	1.56	2755	293	945	157
GO:0045055	regulated exocytosis	5.93E-11	7.91E-08	1.78	2755	161	934	97
GO:0046903	secretion	1.94E-10	2.26E-07	1.67	2755	199	934	113
GO:0032940	secretion by cell	2.14E-10	2.22E-07	1.69	2755	188	934	108
GO:0043299	leukocyte degranulation	7.11E-10	6.64E-07	1.83	2755	127	934	79
GO:0022610	biological adhesion	7.56E-10	6.42E-07	2.66	2755	106	439	45
GO:0007155	cell adhesion	7.56E-10	5.88E-07	2.66	2755	106	439	45
GO:0006753	nucleoside phosphate metabolic process	1.05E-09	7.57E-07	1.88	2755	110	944	71
GO:0009117	nucleotide metabolic process	1.14E-09	7.60E-07	1.89	2755	108	944	70
GO:0002275	myeloid cell activation involved in immune response	1.20E-09	7.45E-07	1.82	2755	130	934	80
GO:0002682	regulation of immune system process	1.97E-09	1.15E-06	1.75	2755	333	496	105
GO:0002274	myeloid leukocyte activation	2.23E-09	1.23E-06	1.77	2755	142	934	85

GO:0006952	defense response	2.52E-09	1.31E-06	2.02	2755	191	522	73
GO:0044282	small molecule catabolic process	2.58E-09	1.27E-06	2.04	2755	88	874	57
GO:0002366	leukocyte activation involved in immune response	3.84E-09	1.80E-06	1.72	2755	154	934	90
GO:0002263	cell activation involved in immune response	3.84E-09	1.71E-06	1.72	2755	154	934	90
GO:0051186	cofactor metabolic process	4.63E-09	1.96E-06	1.91	2755	98	944	64
GO:0002283	neutrophil activation involved in immune response	5.02E-09	2.04E-06	1.81	2755	122	934	75
GO:0045321	leukocyte activation	7.53E-09	2.93E-06	1.55	2755	216	997	121
GO:0043312	neutrophil degranulation	9.64E-09	3.60E-06	1.8	2755	121	934	74
GO:0002683	negative regulation of immune system process	1.21E-08	4.34E-06	2.67	2755	79	496	38
GO:0007165	signal transduction	1.44E-08	4.97E-06	1.34	2755	720	718	251
GO:0036230	granulocyte activation	2.30E-08	7.69E-06	1.77	2755	127	934	76
GO:0042119	neutrophil activation	2.51E-08	8.10E-06	1.77	2755	125	934	75
GO:1901135	carbohydrate derivative metabolic process	3.61E-08	1.12E-05	1.66	2755	162	944	92

*GO Term analysis was performer by GOrilla online tool and showed GO Terms are referring to group „Processes“.

Enrichment = (b/n) / (B/N)
N - is the total number of genes
B - is the total number of genes associated with a specific GO term
n - is the number of genes in the top of the user's input genes
b - is the number of genes in the intersection

Supplemental Table 7: List of genes involved in PI3K/Akt signalling whose expression is changed during idelalisib therapy in vivo (adjusted P-value < 0.05; base mean > 100).

Gene ID	Gene name*	Base Mean (reads per million)	Fold Change (log2)**	Adjusted P-value
ENSG00000128340	RAC2	11122	-0.93041	6.15E-14
ENSG00000100889	PCK2	1230	-0.68985	5.09E-10
ENSG00000182866	LCK	3171.9	-0.6158	3.63E-08
ENSG00000160712	IL6R	1129.9	-0.85642	7.17E-08
ENSG00000139626	ITGB7	7507.1	-0.66886	9.34E-08
ENSG00000186350	RXRA	3513.3	0.948381	1.12E-06
ENSG00000141968	VAV1	3215.8	-0.44028	1.76E-06
ENSG00000111276	CDKN1B	3643.5	0.423518	3.18E-06
ENSG00000121879	PIK3CA	3075.9	0.449979	3.52E-06
ENSG00000068971	PPP2R5B	1110.1	-0.546	4.19E-06
ENSG00000104812	GYS1	1845.1	-0.38716	5.37E-06
ENSG00000213231	TCL1B	310.6	0.787017	8.21E-06
ENSG00000184574	LPAR5	1252.6	-0.37624	1.12E-05
ENSG00000118046	STK11	2676.8	0.375179	3.52E-05
ENSG00000096968	JAK2	3725.7	-0.4422	6.11E-05
ENSG00000123374	CDK2	999.4	0.458141	6.35E-05
ENSG00000185950	IRS2	989.5	0.642416	6.35E-05
ENSG00000134460	IL2RA	3207.5	0.401875	8.85E-05
ENSG00000109458	GAB1	5219.9	0.405891	0.000114
ENSG00000105568	PPP2R1A	3756.9	-0.28079	0.000128
**ENSG00000150907**	**FOXO1**	**4549.6**	**0.434655**	**0.000148**
ENSG00000177105	RHOG	936.9	-0.4025	0.000176
ENSG00000174021	GNG5	655	-0.40449	0.000204
ENSG00000118971	CCND2	1816.6	-0.37347	0.000229
ENSG00000141564	RPTOR	1430.9	0.372897	0.000244
ENSG00000166225	FRS2	1623.4	0.295375	0.000285
ENSG00000141506	PIK3R5	784.7	0.554884	0.000441
ENSG00000186469	GNG2	3191.1	-0.29361	0.000534
ENSG00000167393	PPP2R3B	1287.3	0.497228	0.000613
ENSG00000132155	RAF1	6238.8	0.184951	0.000946
ENSG00000105173	CCNE1	221.4	-0.6688	0.000966
ENSG00000176533	GNG7	3677.1	0.31371	0.001003
ENSG00000033327	GAB2	8219.8	0.214818	0.003197
**ENSG00000164327**	**RICTOR**	**4947**	**0.282368**	**0.00398**
ENSG00000112640	PPP2R5D	1288.4	-0.19388	0.003992
ENSG00000118689	FOXO3	1653.9	0.354737	0.004963
ENSG00000100385	IL2RB	611.3	-0.47867	0.005409
ENSG00000105401	CDC37	8694.9	0.177377	0.005409
ENSG00000187840	EIF4EBP1	249.2	-0.35914	0.005551
ENSG00000114013	CD86	1309.1	-0.33558	0.00713
ENSG00000259384	GH1	123.6	0.54956	0.008298
ENSG00000135930	EIF4E2	2228.7	-0.24923	0.009772

ENSG00000137154	RPS6	76400	0.129004	0.009857
ENSG00000170962	PDGFD	606.5	-0.37458	0.010081
ENSG00000161638	ITGA5	578.1	0.429797	0.01013
ENSG00000198793	MTOR	5071.1	0.174079	0.011327
ENSG00000143384	MCL1	15436.3	-0.23623	0.011693
ENSG00000165699	TSC1	6041.1	0.154885	0.011865
ENSG00000166913	YWHAB	6444.4	-0.19701	0.013244
ENSG00000135424	ITGA7	107.1	-0.45908	0.013809
ENSG00000130702	LAMA5	4845.7	-0.32616	0.013867
ENSG00000184481	FOXO4	1489.1	0.266427	0.017169
ENSG00000128272	ATF4	15688.5	0.214615	0.018133
ENSG00000115966	ATF2	1832.6	0.161379	0.022351
ENSG00000167965	MLST8	901.3	-0.24166	0.027679
ENSG00000108443	RPS6KB1	1754.1	0.189615	0.028005
ENSG00000069966	GNB5	3948.7	0.215486	0.028932
ENSG00000175470	PPP2R2D	1372.3	0.208787	0.033285
ENSG00000136869	TLR4	154.5	-0.4825	0.034567
ENSG00000121594	CD80	111.4	-0.45781	0.038368

* underlined genes were changed during ibrutinib therapy in vivo as well
** positive numbers mean a relative upregulation during idelalisib therapy in vivo

Used gene sets: Reactome_PI3K_AKT_signalling_in_cancer (no. M27162) and KEGG: PI3K-Akt signaling pathway in Homo sapiens (has04151)

Supplemental Table 8: Characteristics of CLL patients

														**Proteins detected (A=Akt, R=Rictor, F=FoxO1)**					
**No.**	**Used in figure**	**Gender ***	**Age at the time of diagnosis**	**Rai stage (at sample collection)**	**del17p13 (%)**	**del11q23 (%)**	**trisomy 12 (%)**	**del13q14 (%)**	**IgHv status**	**% of IgHV homology**	**TP53**	**Treat-ment ****	**Timepoints for RNAseq if applicable**	**pre-therapy**	**week 1**	**week 2**	**week 4**	**week 6-8 (data not shown for A in ibr)**	**week 11-12**
1	1B, 1E, 2D, S1A, S1B, S1D	M	59	IV	neg	neg	neg	pos(80)	mut	96.26	wt	ibr		A, R			A, R		
2	2D	M	57	I	neg	neg	neg	neg	unmut	98.26	wt	ibr		A, R			A, R		A, R
3	2D	F	48	I	neg	pos(90)	neg	pos(95)	unmut	100	wt	ibr		A, R			A, R		A, R
4	1B, 1E, 2D, S1A, S1B, S1D	M	60	I	pos(15)	neg	pos(91)	neg	unmut	100	mut/del	ibr		A, R			A, R		A, R
5	1B, 1E, 2D, S1A, S1B, S1D	M	66	0	neg	neg	neg	pos(79)	mut	91.03	wt	ibr		A, R			A, R		A, R
6	1B, 1E, 2D, S1A, S1B, S1D	M	73	0	neg	neg	neg	pos(49)	mut	97.37	wt	ibr		A, R			A, R		A, R
7	1B, 1E, 2D, S1A, S1B, S1D	M	65	I	pos(58)	neg	neg	neg	unmut	100	mut/del	ibr		A, R			A, R		A, R
8	1B, 1E, 2D, S1A, S1B, S1D	M	61	II	neg	pos(95)	neg	neg	unmut	100	wt	ibr		A, R			A, R		A, R
9	1B, 1E, 2D, S1A, S1B, S1D	M	67	0	neg	neg	neg	pos(89)	mut	97.59	wt	ibr		A, R			A, R		A, R
10	1B, 1E, 2D, S1A, S1B, S1D	M	72	I	neg	pos(90)	neg	neg	unmut	100	wt	ibr		A, R			A, R		A, R
11	1B, 1E, 2D, S1A, S1B, S1D	M	48	I	neg	pos(99)	neg	neg	unmut	100	wt	ibr		A, R			A, R		A, R
12	1B, 1E, 2D, S1A, S1B, S1D	F	72	IV	pos(70)	neg	neg	neg	mut	93.88	mut/del	ibr		A, R			A, R		A, R

13	2D, 3B, S1C, S4	M	67	IA	neg	pos (86)	neg	pos (11)	unmut	100	wt	ibr		A, R, F			A, R, F		
14	2D, 3B, S4	M	43	ND	neg	pos (96)	pos (80)	neg	unmut	100	wt	ibr		A, R, F	A	A	A, R, F		A
15	1B, 1E, 2A, 3A, 3B, S1A, S1B, S1D	M	57	ND	neg	pos (12)	neg	pos (84)	mut	93.8	wt	ibr	pre, day 15	A, F		A	A, F		
16	1B, 1E, 2A, 2D, 3B, S1A, S1B, S1D, S4	M	64	III B	neg	neg	neg	neg	unmut	100	wt	ibr	pre, week 9	A, R, F	A	A	A, R, F		A
17	1A, 1B, 1E, 2D, 3B, 6G, S1B, S1C, S1D, S4, S14B	M	67	ND	neg	pos (95)	neg	neg	unmut	100	wt	ibr		A, R, F		A, R, F		A	A
18	1B, 1E, 2D, 3B, S1B, S1D, S4, S14B	M	52	IVA	neg	neg	neg	neg	unmut	100	wt	ibr		A, R, F			A, R, F		A
19	2A	M	34	I	pos (16)	neg	neg	neg	mut	96.6	mut/del	ibr	pre, day 15						
20	2A	F	51	IV	neg	neg	pos (52)	neg	unmut	100	ND	ibr	pre, day 15						
21	2A	M	75	I	neg	neg	neg	pos (79)	mut	96.6	ND	ibr	pre, week 12						
22	2A	M	56	I	pos (36)	neg	neg	pos (5)	unmut	100	mut/del	ibr	pre, week 5						
23	2A	F	67	IV	pos (94)	pos (94)	neg	neg	unmut	100	mut/del	ibr	pre, week 5						
24	1B, 2D, 3B, S1A, S1B, S4, S14B	M	58	ND	pos (90)	neg	neg	pos (60)	mut	92.27	mut/del	ibr		A, R, F		A, R, F			
25	2D, 3B, S1C, S4	F	68	IV	neg	neg	neg	neg	unmut	98.24	mut	ibr		A					A, R, F
26	1B, 1E, 2D, 3A, 3B, S1B, S1D, S4	M	51	IVA	neg	neg	neg	neg	unmut	100	wt	ibr		A, R, F			A, R, F	A	
27	2D, 3B, S1C, S4	F	64	ND	neg	neg	neg	pos (8)	mut	91.6	wt	ibr		A, R, F	A	A	A, R, F	A	
28	2D, 3B, 6G, S1C, S4	F	76	IIIB	neg	pos (18)	neg	neg	unmut	99.66	wt	ibr		R			R, F		
30	2D, 3B, S1C, S4	M	48	IVB	neg	neg	neg	pos (85)	unmut	100	wt	ibr		A, R, F	A		A, R, F		

31	1B, 1E, 2D, 3B, 6G, S1A, S1B, S1C, S1D, S4	F	45	III	pos (100)	neg	neg	pos (100)	mut	95.14	mut/del	ibr		A, R					A, R, F
32	1B, 1E, 1E, 2D, 3B, S1A, S1B, S1C, S1D, S4	M	37	ND	pos (85)	pos (95)	neg	pos (14)	unmut	100	mut/del	ibr		A, R, F			A, R, F		A
33	1E, 2D, 3B, S1C, S4, S14B	F	47	ND	neg	neg	neg	neg	unmut	ND	wt	ibr		A, R, F			A, R, F		
34	1B, 1E, 1F, 2D, 3B, S1A, S1B, S1C, S1D, S4	F	unknown	ND	pos	neg	neg	pos	mut	89.47	mut	ibr		A, R, F			A, R, F		
35	1F, 2D, 3B, 6G, S1C, S4	M	64	IIB	neg	pos (92)	neg	pos (96)	unmut	100	wt	ibr		A, R, F			A, R, F	A	
36	1A, 3B	M	57	IV	pos (60.5)	neg	neg	pos (96)	mut	92.18	mut/del	ibr		A, F		A, F			A
37	2D, 3B, 7F, G, S1C, S4, S11B	M	34	IA	neg	pos (74)	neg	pos (15)	unmut	100	mut	ibr		A, R, F	A	A	A, R, F	A	A
38	1B, 1E, 3B, S1A, S1B S1D	M	39	IVB	pos (12)	pos (85)	neg	pos (15)	unmut	100	mut/del	ibr		A, R, F	A	A	A, R, F	A	
39	1B, 1E, 2A, 2D, 3B, S1A, S1B, S1D, S4	M	59	IVB	neg	pos (80)	neg	pos (83)	unmut	100	wt	ibr		A, R, F	A	A	A, R, F		
40	1B, 1E, 2C, 2D, 3B, S1A, S1B, S1C, S1D, S4	M	56	ND	pos (62)	neg	neg	pos (64)	unmut	100	mut/del	ibr	pre, day 14	A, R, F		A		A, R, F	A
41	1B, 1E, 2D, 3B, S1A, S1B, S1C, S1D, S4	M	51	IA	neg	neg	neg	pos (88)	unmut	98.6	wt	ibr		A, R, F		A, R, F			
42	1B, 1E, 1F, 2D, 3B, S1A, S1B,	M	66	IVA	pos (18)	neg	neg	neg	unmut	100	mut/del	ibr		A, R, F			A, R, F		

	S1C, S1D, S4																		
43	1A, 1B, 1E, 2A, 3B, S1A, S1D	F	63	ND	neg	pos (31)	neg	pos (85)	biclon	100; 95.3	wt	ibr	pre, day 15	A, F		A	A, F		
44	2D, 3B, S1C, S4	M	60	IIA	neg	neg	neg	pos (88)	unmut	100	mut	ibr		A, R, F	A	A	A, R, F		
45	1B, 1E, 1F, 2D, 3A, 3B, S1A, S1B, S1C, S1D, S4	F	54	I	neg	neg	pos (85)	neg	unmut	100	wt	ibr		A, R, F		A, R, F		A	
46	1B, 1E, 2D, 3B, S1A, S1B, S1C, S1D, S4	F	70	ND	neg	neg	neg	pos (93)	unmut	100	wt	ibr		A, R, F		A, R, F			
47	1B, 1E, 2A, 2C, 2D, 3B, S1A, S1B, S1D, S4, S13	M	47	IB	pos (9)	neg	neg	neg	unmut	100	mut/del	ibr	pre, week 2	A, R, F		A	A, R, F		
48	1B, 1E, S1C, S1D	M	71	IVB	pos (85)	pos (15)	neg	neg	unmut	100	mut/del	ibr		A	A				
49	1B, 1E, 2D, 3B, 6G, S1A, S1B, S1D, S4	M	34	IIIA	neg	neg	neg	pos (94)	biclon (mut; mut)	92.93; 94.44	mut	ibr		A, R, F		A	A, R, F	A	
50	2A	M	54	IA	neg	pos (60)	neg	pos (54)	unmut	100	wt	ibr	pre, day 8						
51	2D, 3B, S4	F	58	ND	neg	pos (ND)	neg	neg	unmut	100	wt	ibr		A, R, F				A, R, F	
52	5A	M	62	IIB	neg	neg	neg	neg	unmut	100	wt	ide	pre, week 5						
53	5A	F	56	N/A	pos (66)	neg	neg	pos (94)	mut	97.2	mut/del	ide	pre, week 4						
54	5C	F	50	IV A	neg	pos (49)	neg	neg	unmut	100	mut	ide		A, R, F			A, R, F		
55	5C	M	78	ND	neg	neg	neg	pos (91)	unmut	98.61	wt	ide		A, R, F			A, R, F		
56	5C	M	52	IA	neg	pos (87)	neg	pos (85)	unmut	100	wt	ide		A, R, F			A, R, F		
57	5C	F	59	ND	neg	pos (93)	neg	neg	unmut	100	wt	ide		A, R, F			A, R, F		
58	5C	F	74	ND	neg	neg	pos (78)	neg	unmut	99.3	wt	ide		A, R, F			A, R, F		
59	5C	M	61	IV A	neg	pos (86)	neg	neg	unmut	100	wt	ide		A, F			A, F		

60	5A	M	57	IV	neg	neg	neg	pos (95)	mut	92.23	wt	ide	pre, week 4						
61	5C, 5A	M	62	IV	neg	pos (96)	neg	pos (94)	unmut	100	wt	ide	pre, week 4	A, R, F				A, R, F	
62	5A	M	56	IV	neg	neg	neg	neg	mut	95.8	mut	ide	pre, week 4						
63	5A	M	75	IV	neg	neg	neg	pos (7)	mut	95.8	ND	ide	pre, week 4						
64	5C, 5A	M	66	IV	neg	neg	neg	pos (58)	mut	92.4	wt	ide	pre, week 4	A, R, F				A, R, F	
65	5A	M	69	I	neg	neg	pos (69)	neg	unmut	99.1	wt	ide	pre, week 4						
66	5C	M	67	ND	neg	neg	neg	pos (98)	mut	97.92	mut	ide		A, R, F			A, R, F		
67	5B, 5C	F	63	IV A	neg	neg	neg	pos (55)	unmut	100	mut	ide		A, R, F			A, R, F		
68	5A, 5C	F	82	IIB	pos (22)	neg	neg	pos (24)	unmut	100	mut/del	ide	pre, week 2	A, F		A, F			
123	S7A, S9D	F	65	IIIB	neg	neg	pos(70)	pos(5)	mut	93.33	mut	acal		A, R, F					A, R, F
124	S7A, S9D	M	38	IVA	pos(23)	pos(18)	neg	neg	unmut	100	mut	acal		A, R, F					A, R, F
125	S7A	M	75	IIIA	ND	ND	ND	ND	unmut	100	mut	acal		A, R, F			A, R, F		
126	S7A, S9D	F	63	IIIA	ND	ND	ND	ND	mut	95.79	ND	acal		A, R, F			A, R, F		
127	S7A	M	48	IIB	neg	neg	neg	neg	mut	96.18	wt	acal		A, R, F			A, R, F		
128	S7A, S9D	F	63	IVA	neg	neg	neg	neg	unmut	100	wt	acal		A, R, F					A, R, F (month 4)
111	7F, G, S3, S7A	M	63	ND	neg	neg	neg	pos(89)	unmut	100	mut	acal		A, R, F					A, R, F
112	7F, G, S3, S7A, S11B	M	44	ND	neg	pos(93)	neg	pos(94)	unmut	100	wt	acal		A, R, F					A, R, F
69	2I, 4H, 7H	M	66	IA	neg	neg	neg	pos (52)	unmut	100	wt								
70	S14A	M	79	IIIA	neg	neg	neg	pos (23)	unmut	100	wt								
71	4H, 7H	M	70	IIB	neg	neg	neg	pos (8)	mut	94.39	wt								
72	S14A	F	64	IIIB	neg	pos (9)	neg	pos (47)	unmut	100	ND								
73	7D	M	58	IA	neg	neg	neg	pos (86)	unmut	98.26	mut								
74	7C, D	F	63	IA	neg	neg	neg	pos (30)	mut	97.22	wt								
75	2L	M	73	0A	neg	neg	neg	pos (64)	biclon (mut; mut)	92.36; 92.98	ND								

76	7H	M	73	ND	neg	neg	neg	pos (95)	mut	92.52	wt
78	S14A	F	65	IIA	neg	neg	neg	pos (95)	unmut	98.6	wt
79	7D	M	72	IVB	ND	ND	ND	ND	mut	89.47	wt
80	2K, 4H	F	50	IIIA	neg	neg	neg	pos (ND)	mut	94.79	wt
82	7C, D	M	58	I A	neg	neg	neg	pos (43)	unmut	100	wt
83	S1A, S1B	M	57	IV	pos (60.5)	neg	neg	pos (96)	mut	92.18	mut/del
84	6D	M	56	IIB	neg	neg	neg	pos (98)	unmut	100	wt
85	4G, 4H, 7H	F	61	I A	neg	neg	neg	pos (46)	mut	97.57	wt
86	6D, S9A	F	64	ND	neg	neg	neg	pos (94)	mut	95.83	wt
87	2K	M	72	IA	neg	neg	neg	pos (95)	mut	87.29	wt
88	6D, S9A	F	70	IA	neg	pos (95)	neg	pos (36)	mut	97.89	wt
89	S14A	F	59	A	neg	pos (64)	neg	pos (97)	unmut	100	wt
90	S9A	F	54	IA	neg	neg	neg	pos (25)	mut	89.24	wt
91	S14A	F	71	IA	neg	neg	neg	pos (59)	mut	97.6	wt
92	S14A	F	59	IIIB	neg	neg	neg	pos (90)	unmut	100	wt
93	S14A	M	74	IVA	neg	neg	neg	pos (99)	unmut	99.65	mut
94	2K, 2L, 6D, 7H, S9A	F	75	0A	neg	neg	neg	neg	mut	94.79	wt
95	2L, 7H	M	65	0A	neg	neg	neg	pos (51)	biclon (mut; mut)	96.88; 95.19	wt
96	2L, 7H	M	60	IIA	neg	neg	neg	pos (ND)	mut	97.92	wt
97	2K, 4H	M	70	II	neg	neg	neg	neg	mut	91.67	wt
98	2L	M	57	IA	neg	neg	neg	neg	mut	92.63	wt
99	S14A	M	70	IA	neg	neg	neg	pos (99)	unmut	99	wt
100	6D	M	65	IIA	neg	neg	neg	pos (79)	unmut	100	wt
101	S14A	F	62	IA	neg	neg	neg	pos (7)	unmut	100	wt
102	7D	F	76	IB	neg	neg	neg	pos (ND)	mut	96.56	wt
103	2J, 2K, 4F, 6C	F	68	IV B	neg	pos (92)	neg	pos (88)	mut	93.75	wt
104	6D, S9A	M	70	IA	neg	neg	neg	pos (ND)	mut	94.74	mut
105	2L, 7H	F	62	IIB	neg	neg	pos(66)	neg	mut	96.14	wt

106	4H, 7H	M	56	IVA	neg	neg	neg	pos (81)	mut	96.53	wt
107	6D, S9A	M	61	IVB	neg	neg	neg	pos (83)	unmut	100	wt
108	S14A	M	71	IIA	neg	neg	neg	pos (14)	unmut	100	wt
109	S9B, C	F	85	IA	ND	ND	ND	ND	unmut	100	ND
110	S3	M	35	ND	neg	neg	neg	pos(95)	unmut	100	mut
113	7A, B, D, S10A	M	62	IA	neg	neg	neg	pos(86)	mut	88.89	wt
114	7A, B	M	54	IA/B	neg	pos	neg	neg	unmut	100	mut
115	7A, B, D, S9C, S10A	M	75	ND	neg	neg	neg	pos(99)	mut	91.67	wt
116	7A, B, D, S10A	M	75	0A	neg	neg	neg	pos	unmut	100	mut
117	7A, B, D, S10A	F	54	IA	neg	neg	neg	pos(62)	mut	94.79	wt
118	7A, B, D, S10A	F	63	IV	neg	neg	neg	pos(60)	mut	90.53	mut
119	S9C	M	56	IVB	ND	ND	ND	ND	unmut	100	ND
120	S9C	M	61	III	neg	neg	neg	pos(99)	unmut	100	mut
121	S9C	F	73	IA	ND	ND	ND	ND	unmut	100	mut
122	S9C	M	84	IV	neg	pos	pos	neg	biclon	96.18; 98.26	wt

*M= male, F= female
**ibrutinib= ibr, idelalisib= ide, acalabrutinib= acal

References

1. Ian Mockridge C, et al. Reversible anergy of sIgM-mediated signaling in the two subsets of CLL defined by VH-gene mutational status. *Blood*. 2007;109(10):4424–4431.
2. Seda V, et al. FoxO1-GAB1 axis regulates homing capacity and tonic AKT activity in chronic lymphocytic leukemia. *Blood*. 2021;138(9):758–772.
3. Naeem A, et al. Pirtobrutinib targets BTK C481S in ibrutinib-resistant CLL but second-site BTK mutations lead to resistance. *Blood Adv*. 2023;7(9):1929–1943.
4. Coelho V, et al. Identification in CLL of circulating intraclonal subgroups with varying B-cell receptor expression and function. *Blood*. 2013;122(15):2664–2672.
5. Sharma S, et al. miR-29 modulates CD40 signaling in chronic lymphocytic leukemia by targeting TRAF4: an axis affected by BCR inhibitors. *Blood*. 2021;137(18):2481–2494.
6. Mraz M, et al. MicroRNA isolation and stability in stored RNA samples. *Biochem Biophys Res Commun*. 2009;390(1):1–4.
7. Pavlasova G, et al. Rituximab primarily targets an intra-clonal BCR signaling proficient CLL subpopulation characterized by high CD20 levels. *Leukemia*. 2018;32(9):2028–2031.
8. Martin M. Cutadapt removes adapter sequences from high-throughput sequencing reads. *EMBnet.journal*. 2011;17(1):10.
9. Hackenberg M, et al. miRanalyzer: a microRNA detection and analysis tool for next-generation sequencing experiments. *Nucleic Acids Res*. 2009;37(Web Server issue):W68-76.
10. Bolger AM, Lohse M, Usadel B. Trimmomatic: a flexible trimmer for Illumina sequence data. *Bioinforma Oxf Engl*. 2014;30(15):2114–2120.
11. Kozomara A, Griffiths-Jones S. miRBase: annotating high confidence microRNAs using deep sequencing data. *Nucleic Acids Res*. 2014;42(Database issue):D68-73.
12. Love MI, Huber W, Anders S. Moderated estimation of fold change and dispersion for RNA-seq data with DESeq2. *Genome Biol*. 2014;15(12):550.
13. Robinson MD, McCarthy DJ, Smyth GK. edgeR: a Bioconductor package for differential expression analysis of digital gene expression data. *Bioinforma Oxf Engl*. 2010;26(1):139–140.
14. Huber W, et al. Orchestrating high-throughput genomic analysis with Bioconductor. *Nat Methods*. 2015;12(2):115–121.
15. Andrews S. FastQC: A Quality Control Tool for High Throughput Sequence Data [Online]. 2010. http://www.bioinformatics.babraham.ac.uk/projects/fastqc/.
16. Dobin A, et al. STAR: ultrafast universal RNA-seq aligner. *Bioinforma Oxf Engl*. 2013;29(1):15–21.
17. Picard Tools - By Broad Institute. http://broadinstitute.github.io/picard/ (accessed 19 Apr2018).
18. Liao Y, Smyth GK, Shi W. The R package Rsubread is easier, faster, cheaper and better for alignment and quantification of RNA sequencing reads. *Nucleic Acids Res*. 2019;47(8):e47.
19. Wang L, Wang S, Li W. RSeQC: quality control of RNA-seq experiments. *Bioinforma Oxf Engl*. 2012;28(16):2184–2185.
20. Okonechnikov K, Conesa A, García-Alcalde F. Qualimap 2: advanced multi-sample quality control for high-throughput sequencing data. *Bioinforma Oxf Engl*. 2016;32(2):292–294.
21. Saunders CT, et al. Strelka: accurate somatic small-variant calling from sequenced tumor-normal sample pairs. *Bioinforma Oxf Engl*. 2012;28(14):1811–1817.
22. Lai Z, et al. VarDict: a novel and versatile variant caller for next-generation sequencing in cancer research. *Nucleic Acids Res*. 2016;44(11):e108.
23. Benjamin D, et al. *Calling Somatic SNVs and Indels with Mutect2*. Bioinformatics; 2019.
24. Koboldt DC, et al. VarScan 2: Somatic mutation and copy number alteration discovery in cancer by exome sequencing. *Genome Res*. 2012;22(3):568–576.
25. McLaren W, et al. The Ensembl Variant Effect Predictor. *Genome Biol*. 2016;17(1):122.
26. Janssens D. *CUT&RUN: Targeted in situ genome-wide profiling with high efficiency for low cell numbers v3*. 2019.
27. Liu N. *Library Prep for CUT&RUN with NEBNext® Ultra™ II DNA Library Prep Kit for Illumina® (E7645) v2*. 2019.
28. Zhu Q, et al. CUT&RUNTools: a flexible pipeline for CUT&RUN processing and footprint analysis. *Genome Biol*. 2019;20(1):192.

29. Bailey TL, Machanick P. Inferring direct DNA binding from ChIP-seq. *Nucleic Acids Res*. 2012;40(17):e128.
30. Mraz M, et al. miR-150 influences B-cell receptor signaling in chronic lymphocytic leukemia by regulating expression of GAB1 and FOXP1. *Blood*. 2014;124(1):84–95.
31. Quah BJC, Warren HS, Parish CR. Monitoring lymphocyte proliferation in vitro and in vivo with the intracellular fluorescent dye carboxyfluorescein diacetate succinimidyl ester. *Nat Protoc*. 2007;2(9):2049–2056.
32. Hoferkova E, et al. Stromal cells engineered to express T cell factors induce robust CLL cell proliferation in vitro and in PDX co-transplantations allowing the identification of RAF inhibitors as anti-proliferative drugs. *Leukemia*. [published online ahead of print: June 14, 2024]. https://doi.org/10.1038/s41375-024-02284-w.
33. Roederer M. Interpretation of cellular proliferation data: Avoid the panglossian. *Cytometry A*. 2011;79A(2):95–101.
34. Xie Z, et al. Gene Set Knowledge Discovery with Enrichr. *Curr Protoc*. 2021;1(3). https://doi.org/10.1002/cpz1.90.
35. Lu J, et al. Multi-omics reveals clinically relevant proliferative drive associated with mTOR-MYC-OXPHOS activity in chronic lymphocytic leukemia. *Nat Cancer*. 2021;2(8):853–864.
36. Dominguez-Sola D, et al. The FOXO1 Transcription Factor Instructs the Germinal Center Dark Zone Program. *Immunity*. 2015;43(6):1064–1074.
