# Supplementary material for: FoxO1/Rictor axis induces a nongenetic adaptation to ibrutinib via Akt activation in chronic lymphocytic leukemia
Source: J Clin Invest. 2024 Oct 22;134(23):e173770. doi: 10.1172/JCI173770 (PMC11601945; doi:10.1172/JCI173770)

FoxO1/Rictor Axis Induces a Non-Genetic Adaptation to Ibrutinib via Akt Activation in Chronic Lymphocytic Leukemia

Ondrisova *et al*.

Uncropped original blots from the manuscript

List of used antibodies

Detected protein	Manufacturer	Catalog number	Dilution
**Rictor**	Cell Signaling	#2114	1:2000
**mTOR**	Cell Signaling	#2983	1:2000
**pmTOR**	Cell Signaling	#2974	1:1000
**FoxO1**	Cell Signaling	#2880	1:2000
**pFoxO1**	Cell Signaling	#2599	1:1000
**Akt**	Cell Signaling	#2920	1:2000
**pAkt^{S473}**	Cell Signaling	#4060	1:1000
**pAkt^{T308}**	Cell Signaling	#13038	1:1000
**BTK**	Cell Signaling	#3533	1:2000
**pBTK**	Cell Signaling	#5082	1:1000
**GAB1**	Cell Signaling	#3232	1:2000
**GSK3α/β**	Cell Signaling	#5676	1:2000
**pGSK3α/β**	Cell Signaling	#9331	1:2000
**cMYC**	Cell Signaling	#5605	1:2000
**GAPDH**	Cell Signaling	#2118	1:2000
**Vinculin**	Santa Cruz	sc73614	1:2000

Full unedited gels for Figure 1A

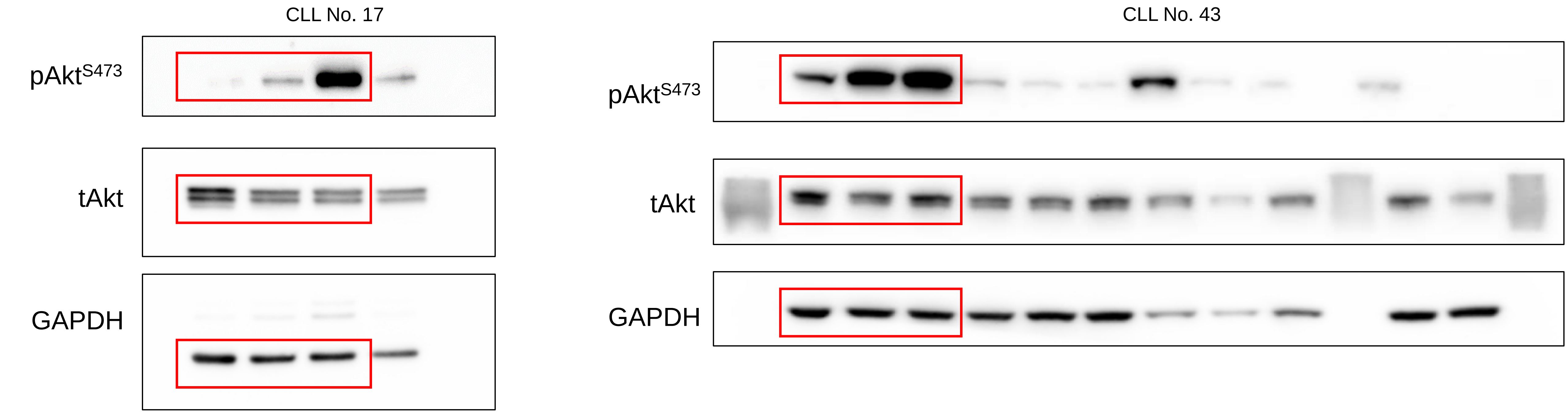

Full unedited gels for Figure 1C

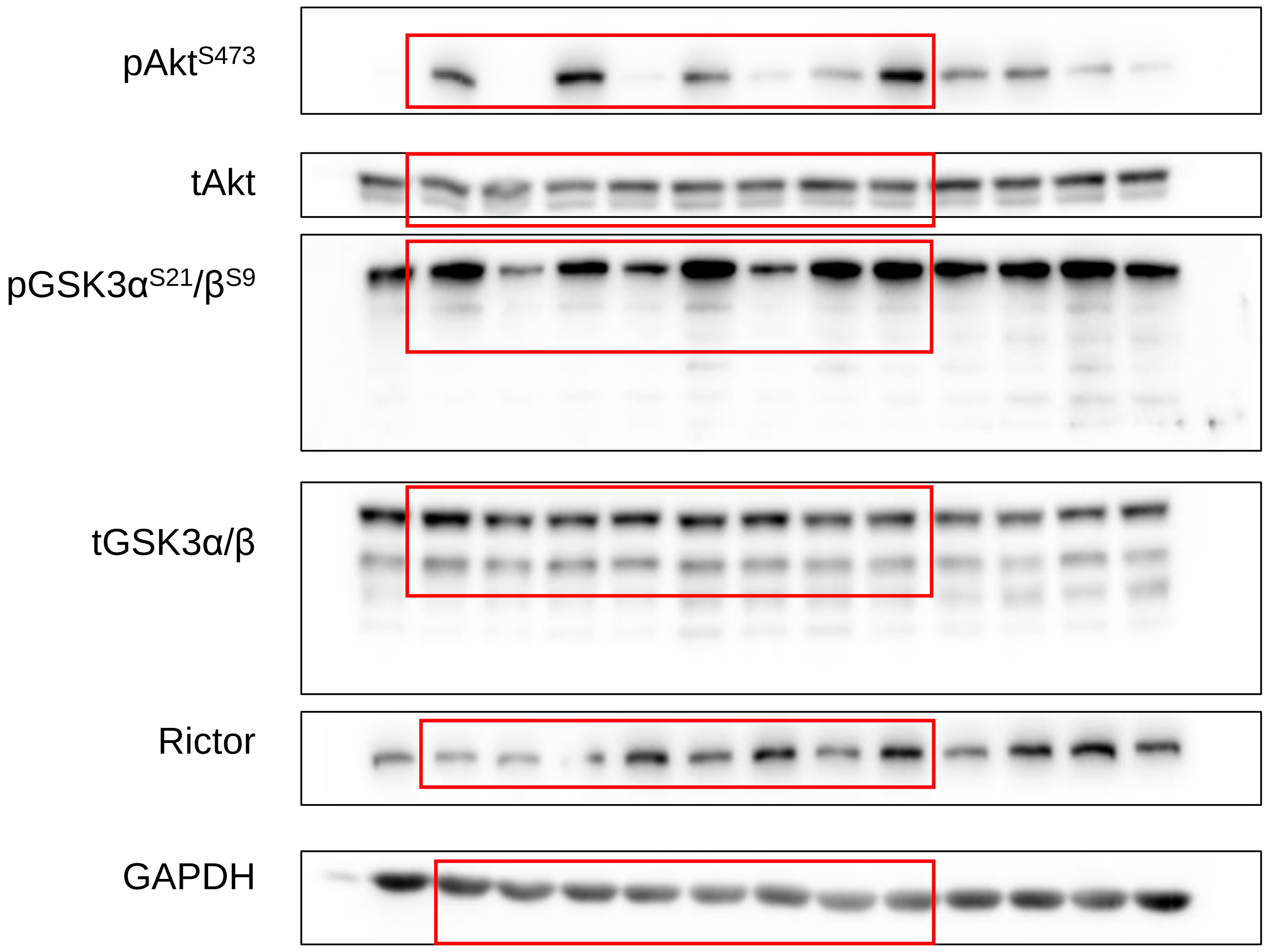

Full unedited gels for Figure 2C

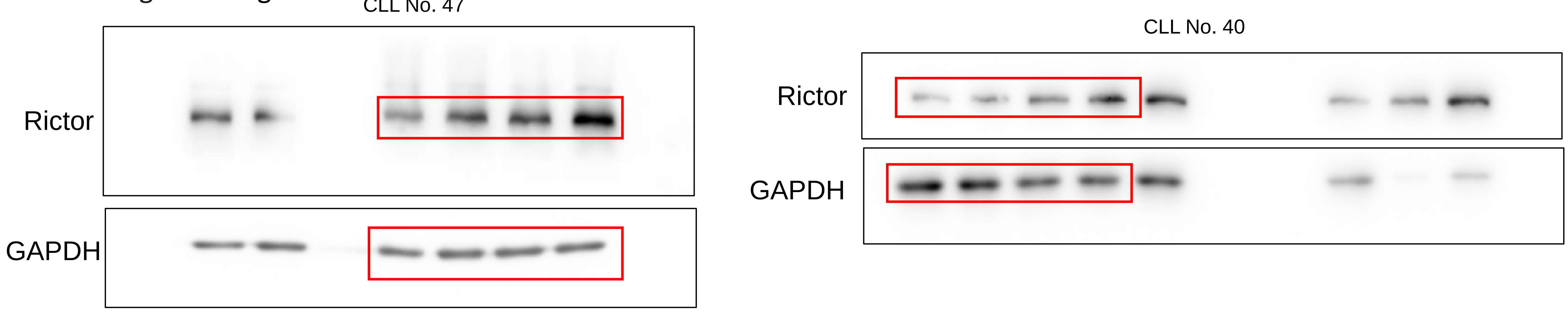

Full unedited gels for Figure 2F

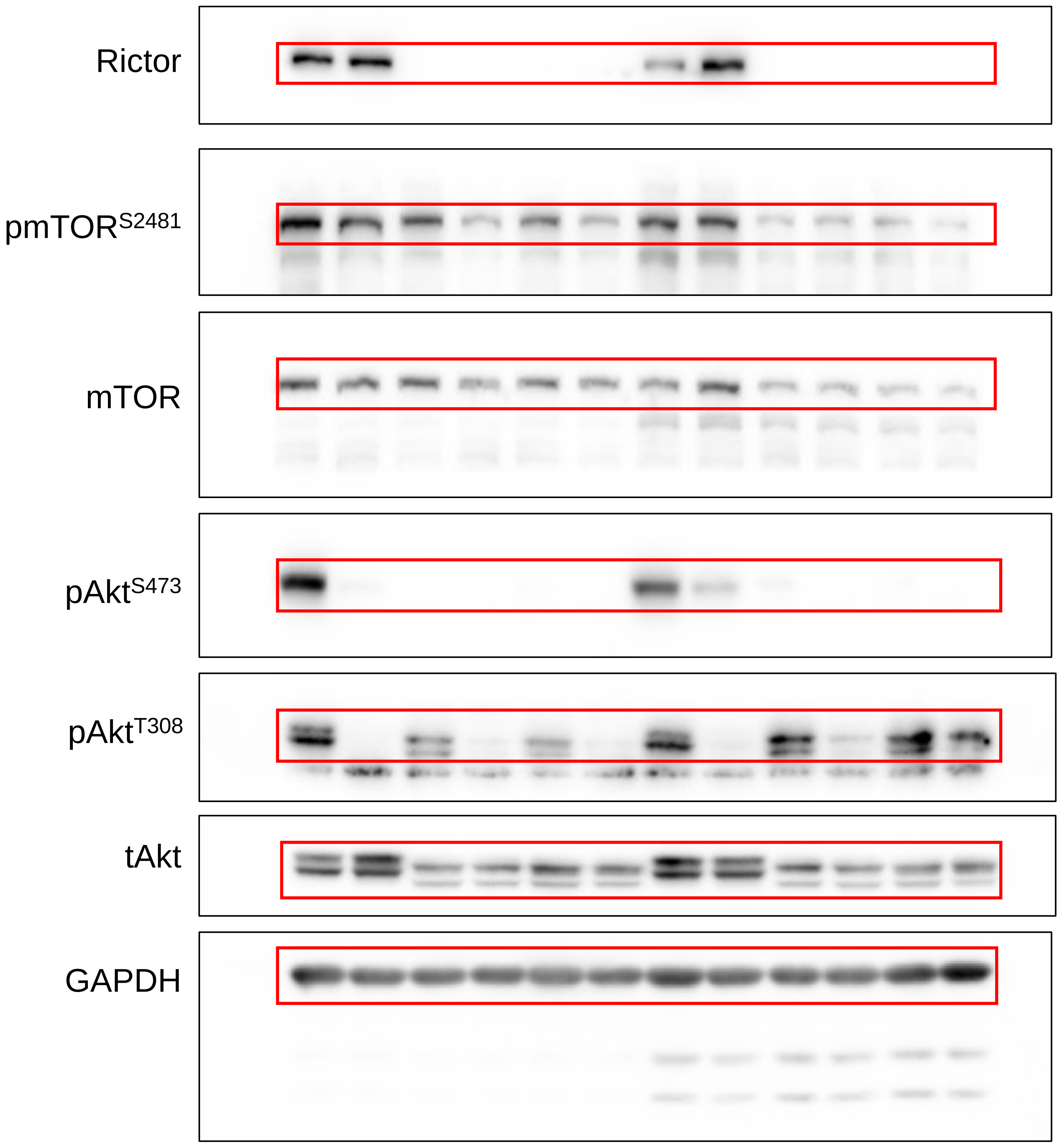

Full unedited gels for Figure 2H

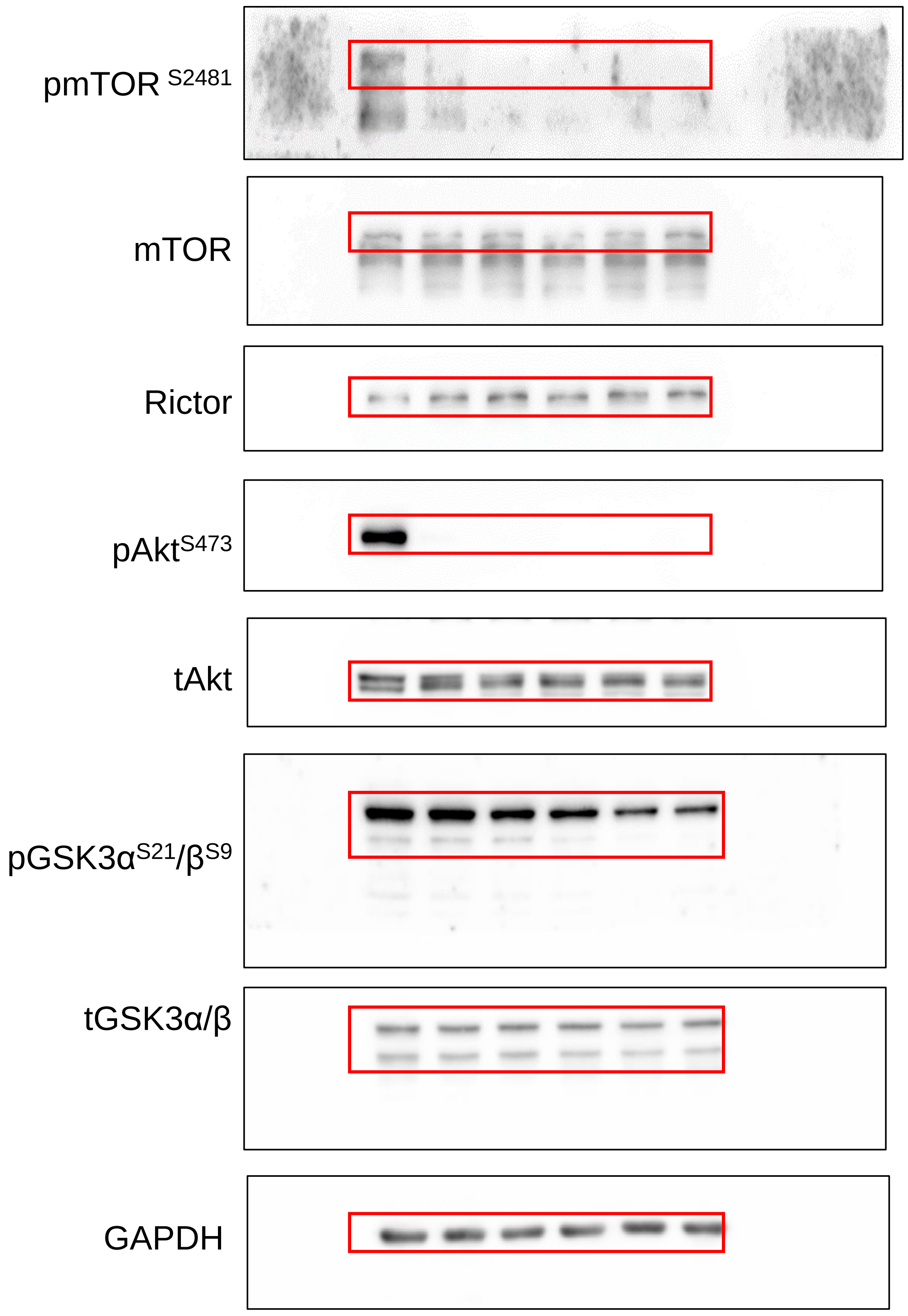

Full unedited gels for Figure 2I

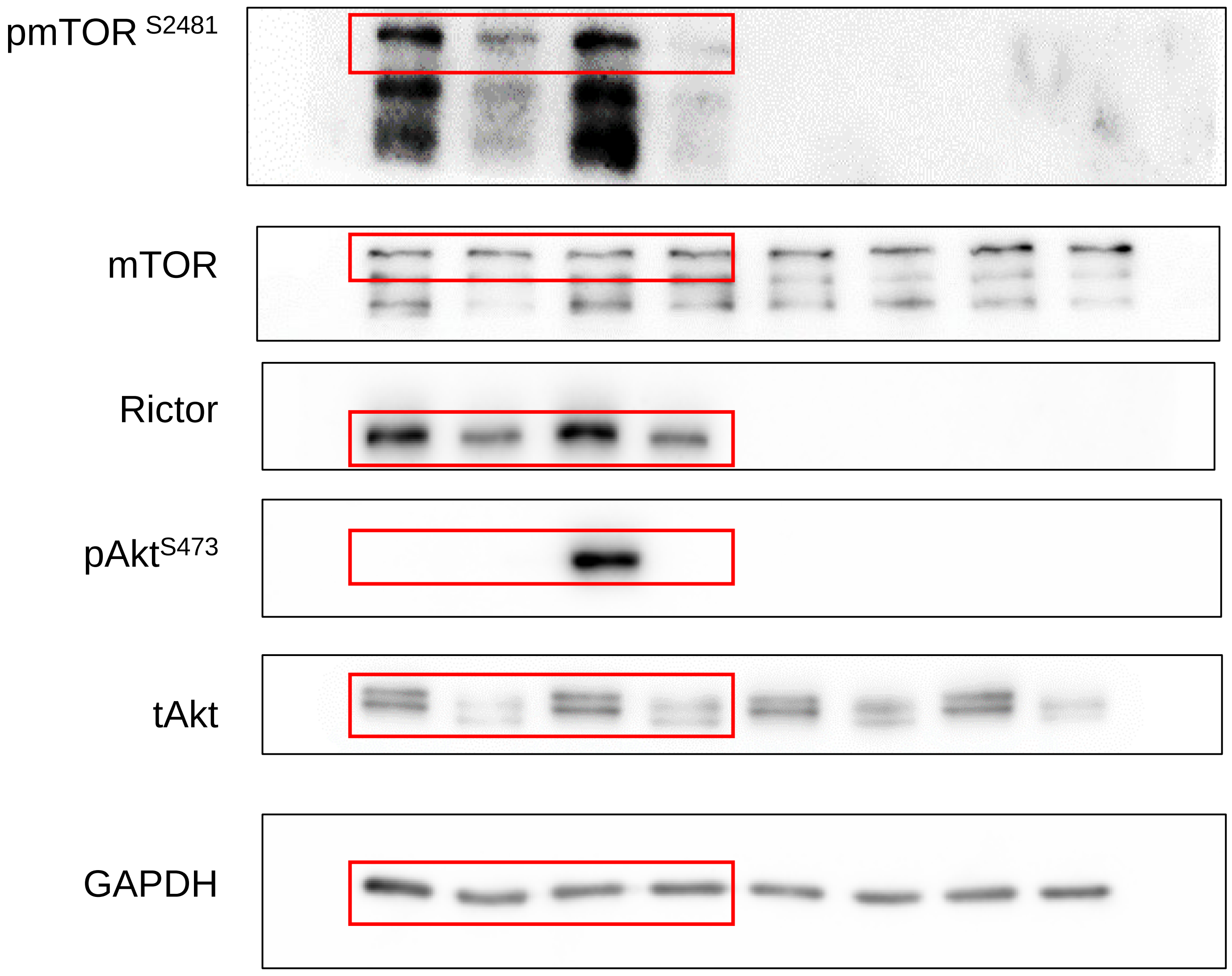

Full unedited gels for Figure 2J

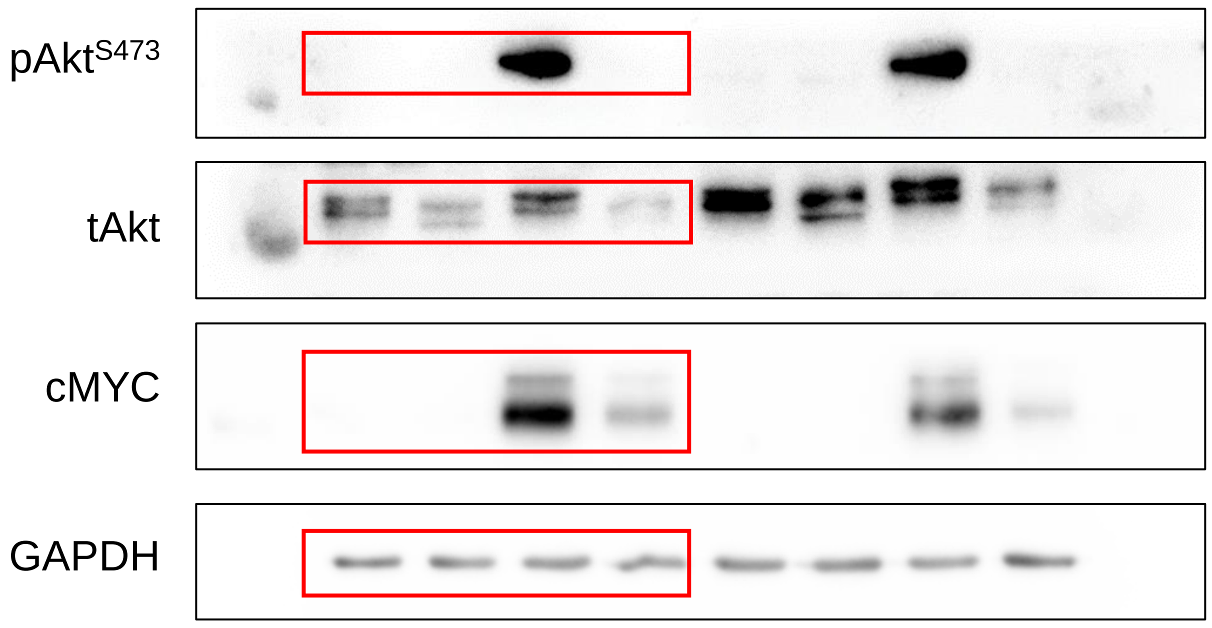

Full unedited gels for Figure 3A

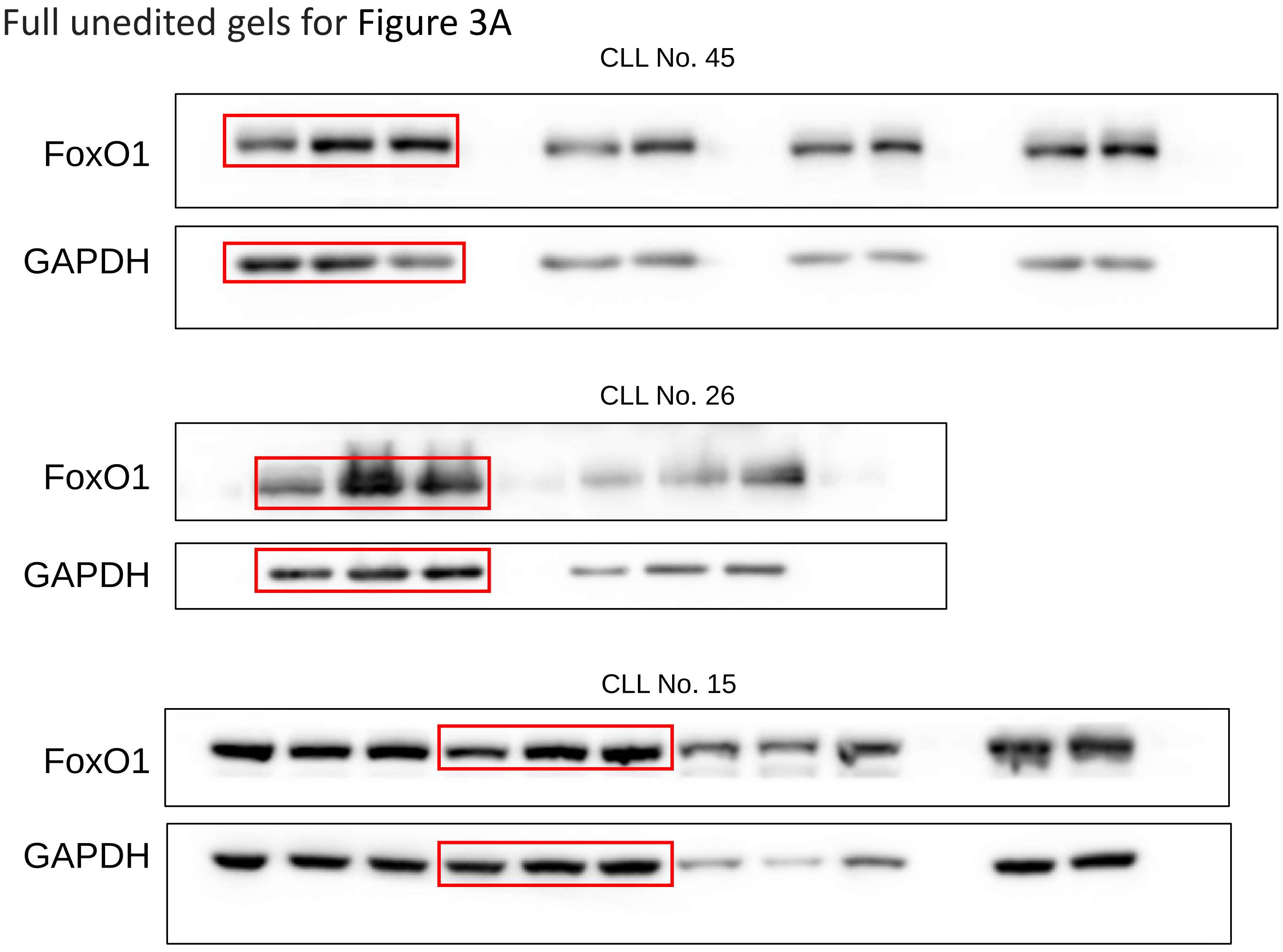

Full unedited gels for Figure 3C

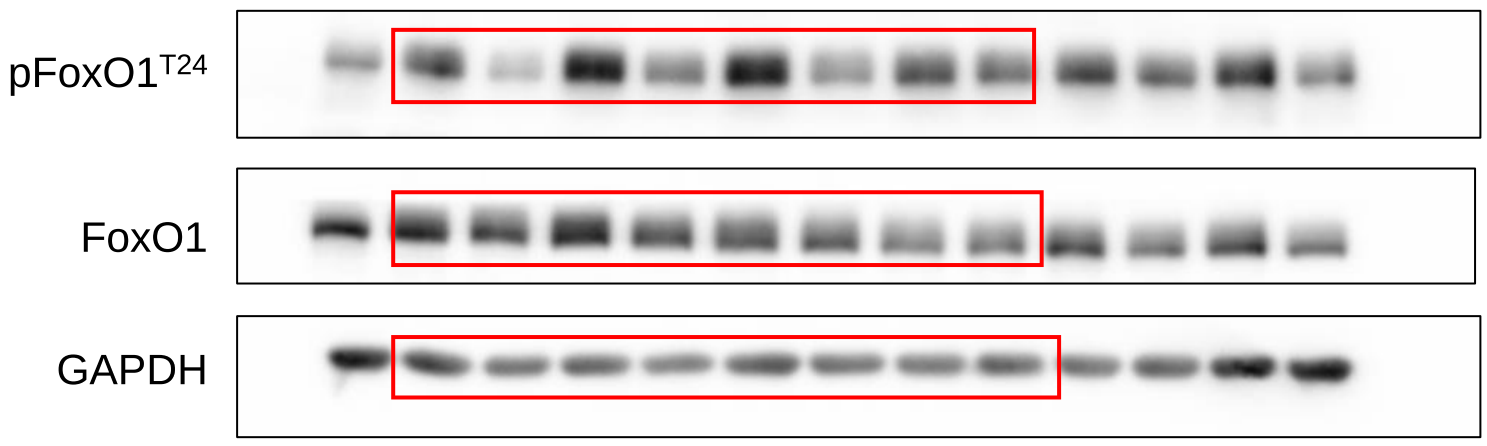

Full unedited gels for Figure 4A

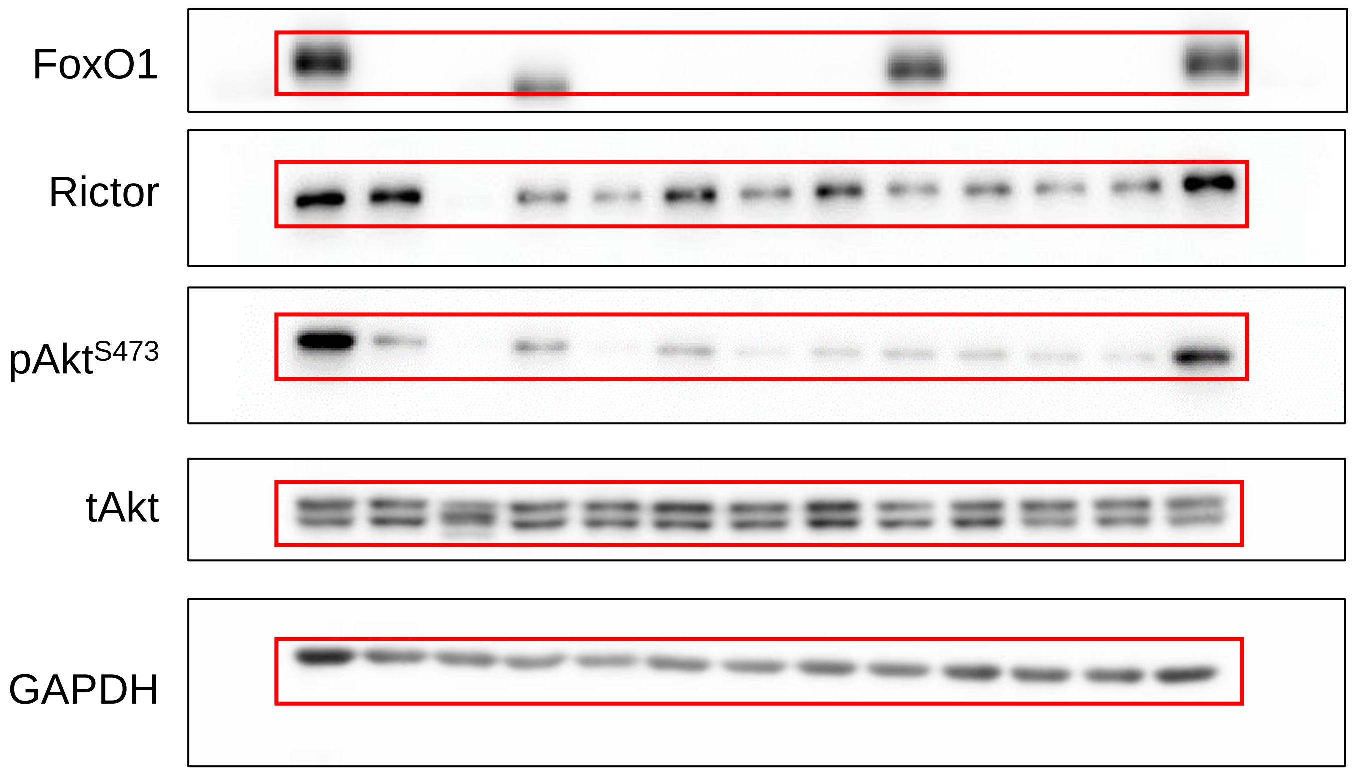

Full unedited gels for Figure 4B

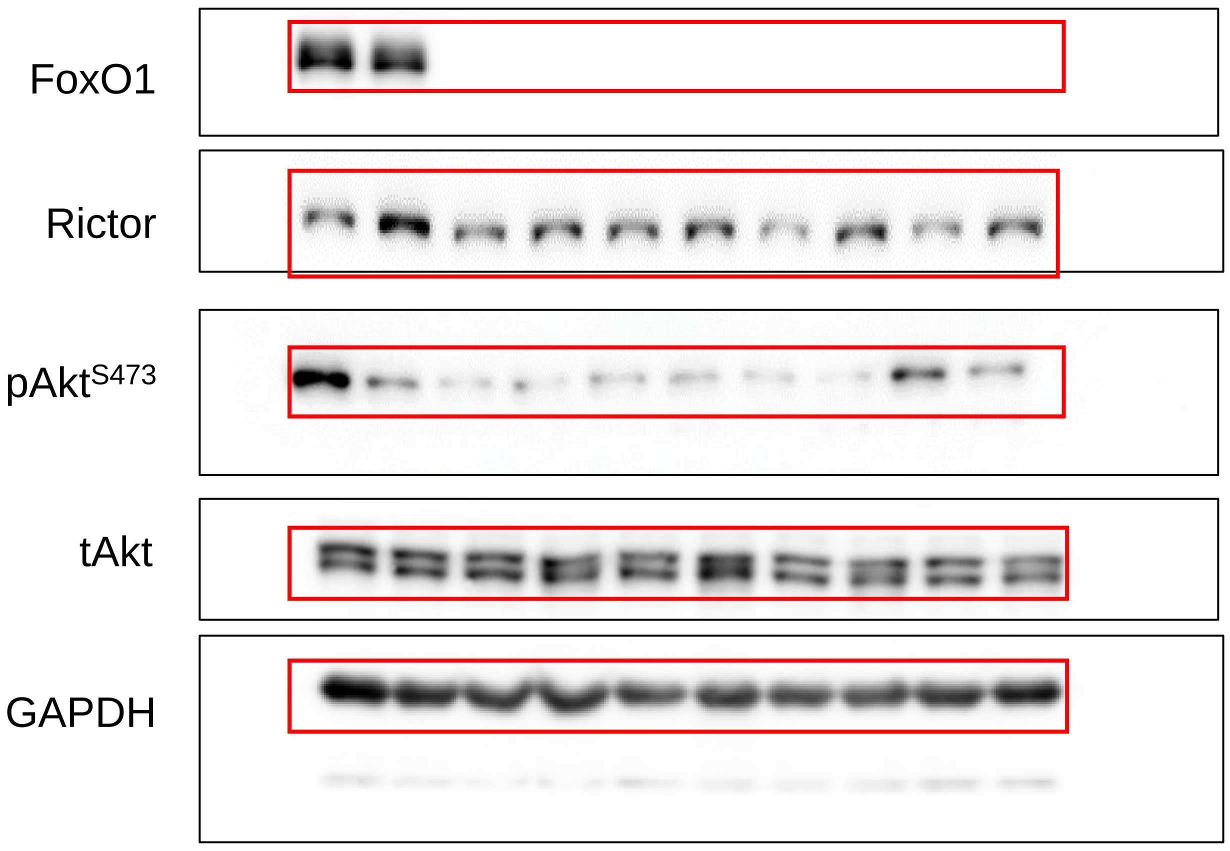

Full unedited gels for Figure 4D

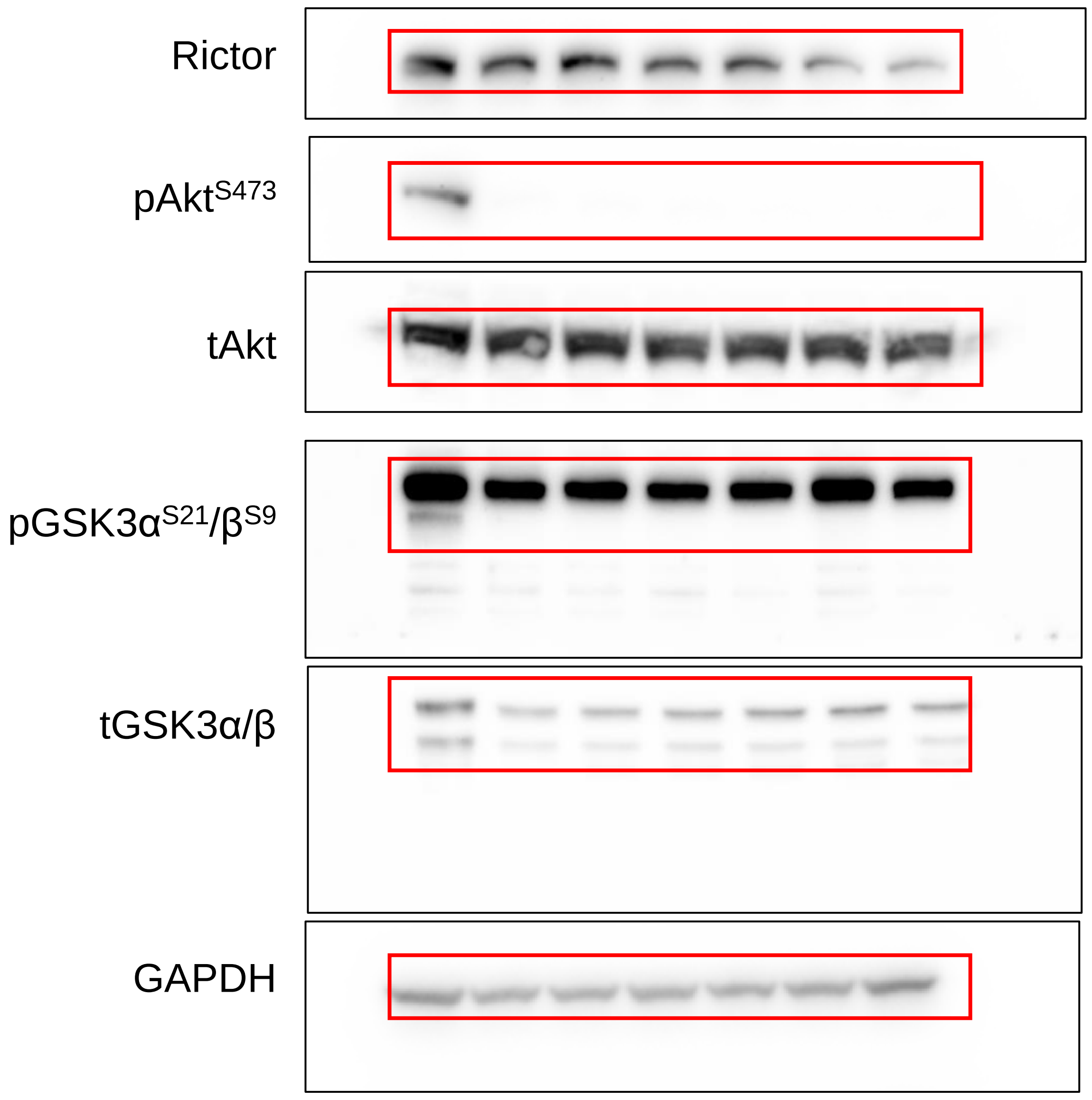

Full unedited gels for Figure 4E

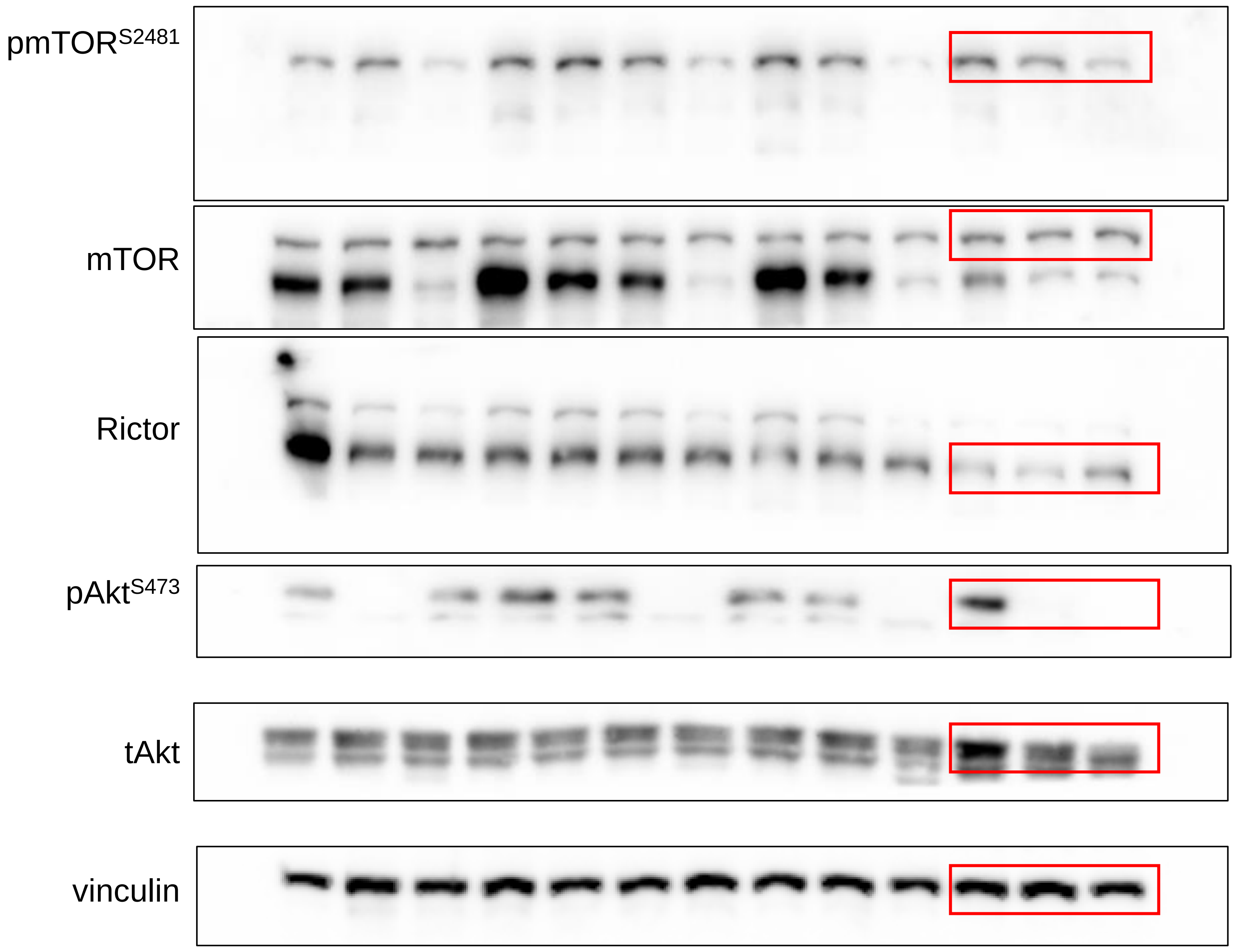

Full unedited gels for Figure 4F

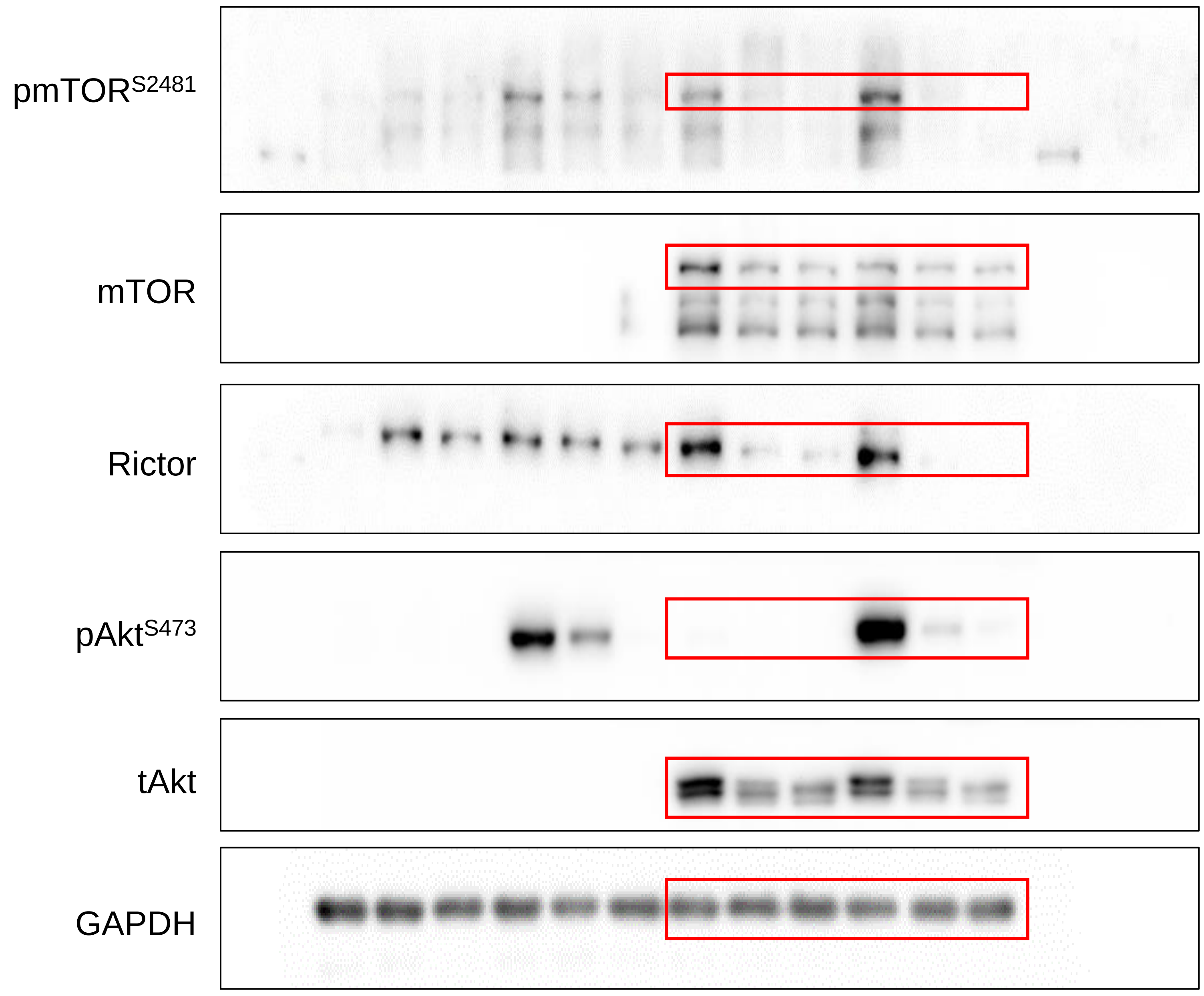

Full unedited gels for Figure 4G

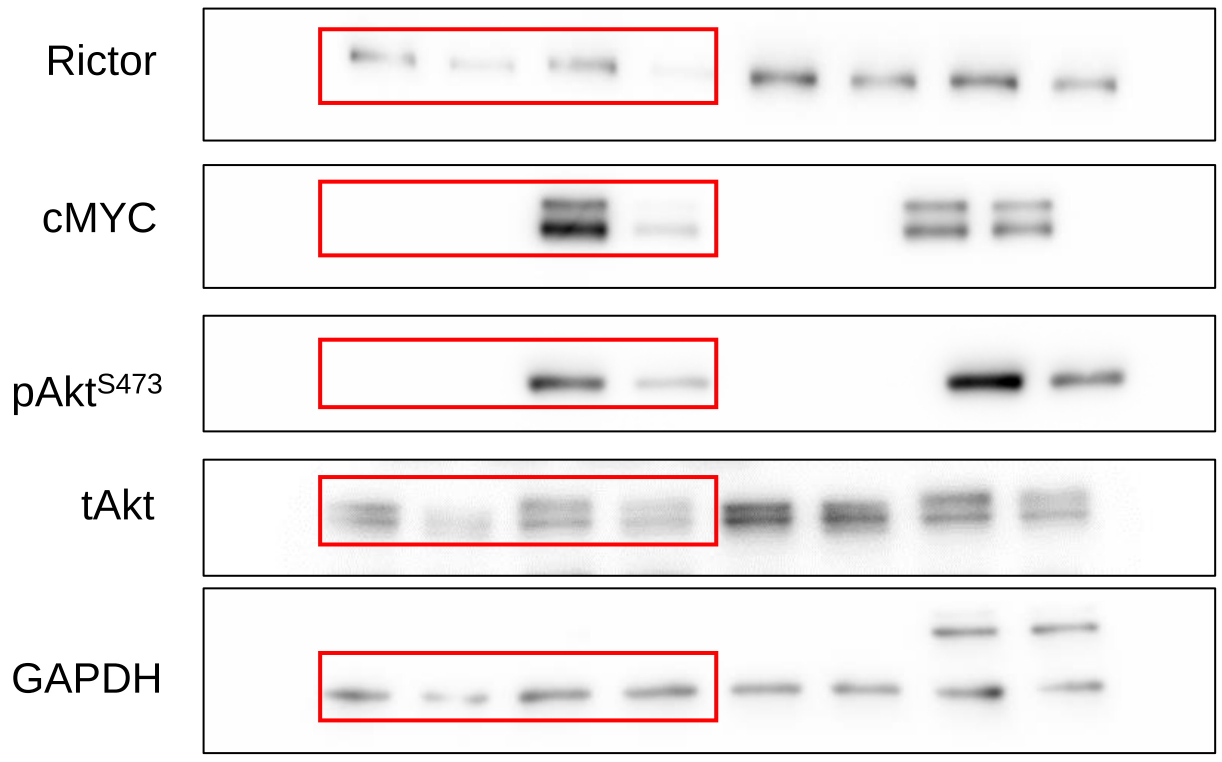

Full unedited gels for Figure 5B

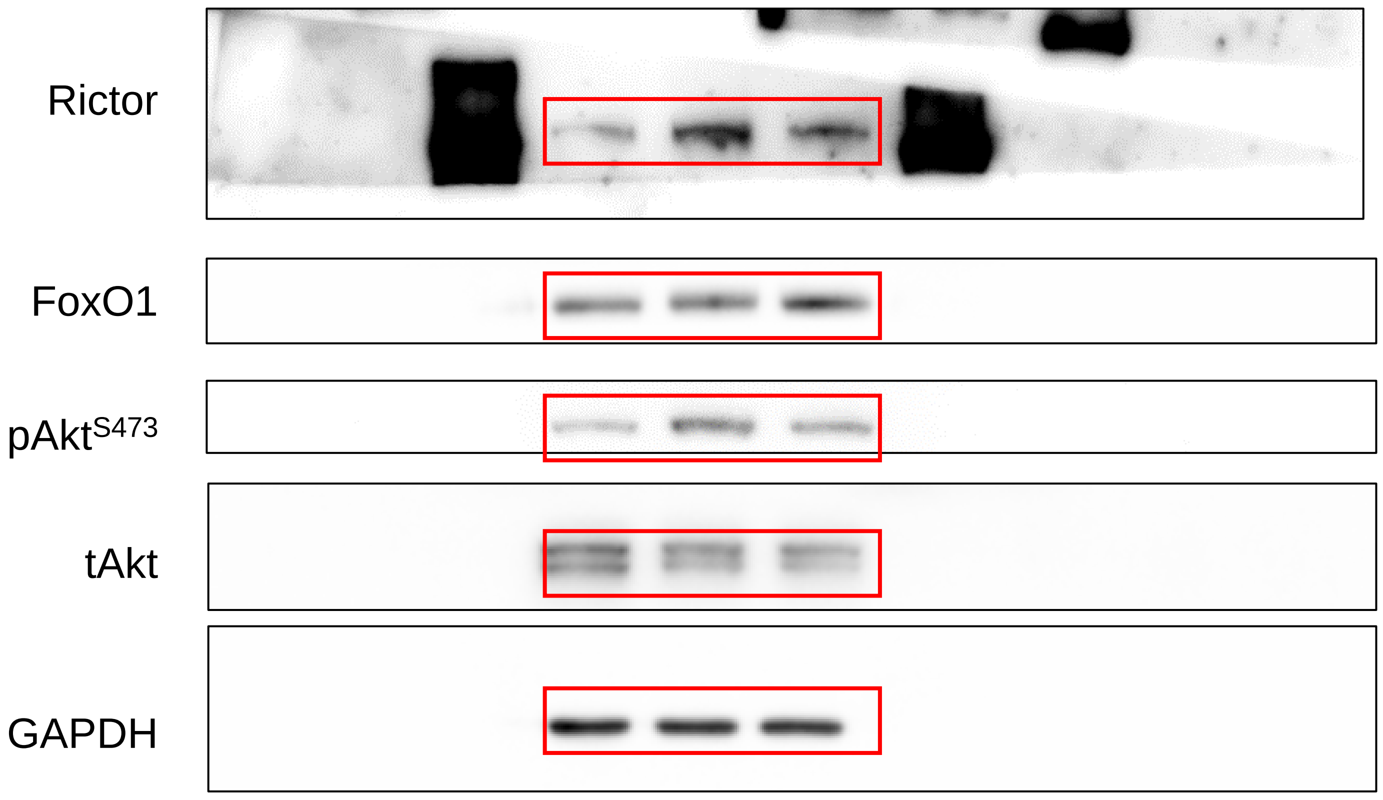

Full unedited gels for Figure 6A

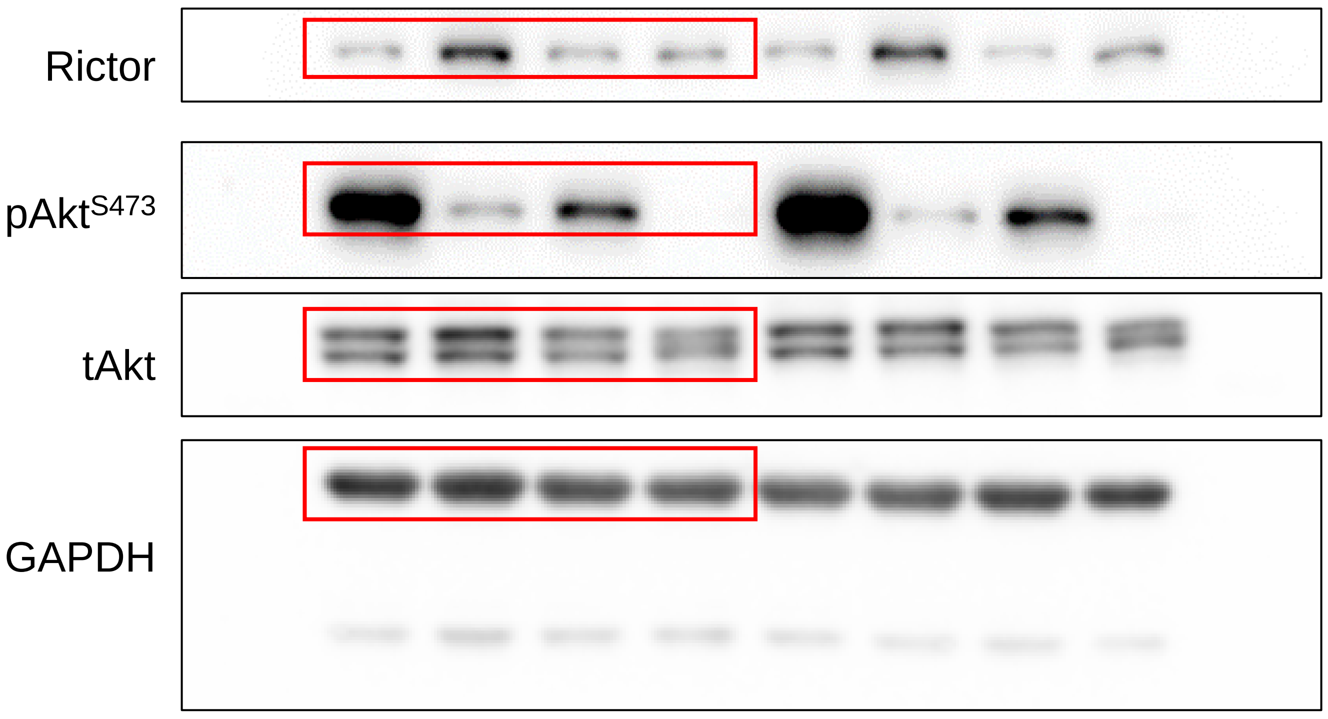

Full unedited gels for Figure 6C

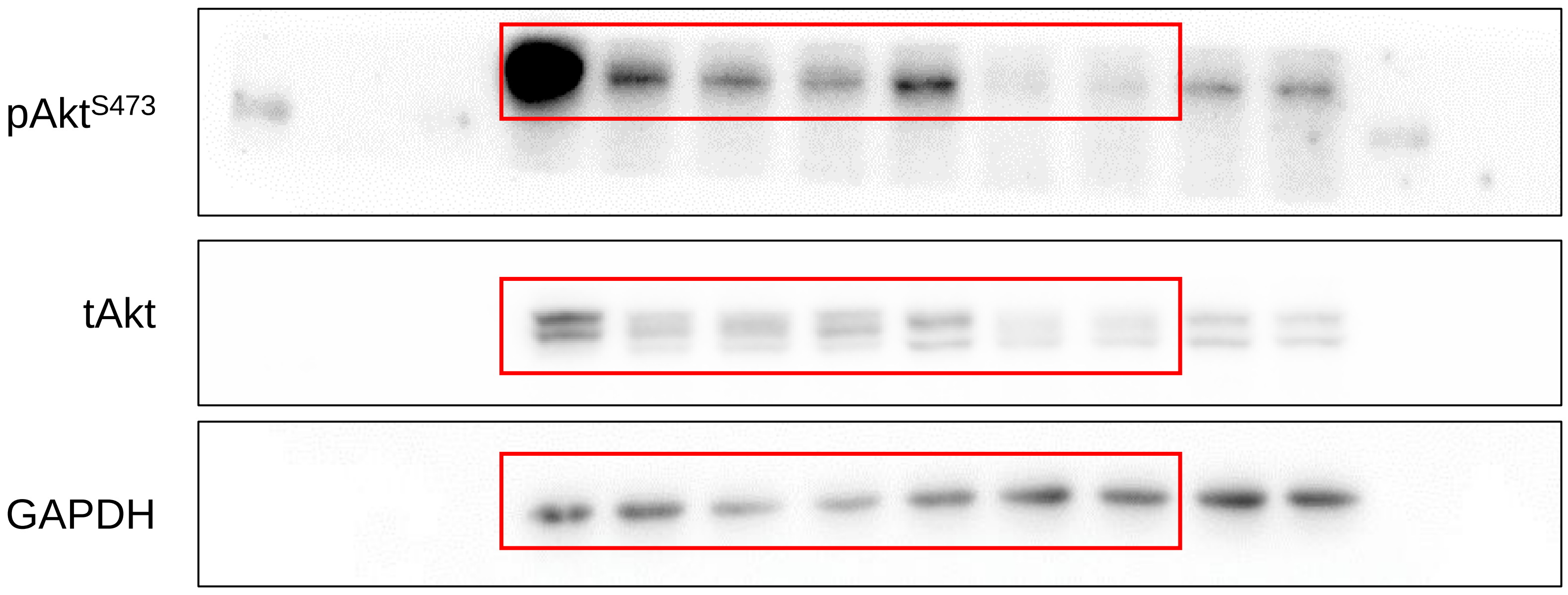

Full unedited gels for Supplemental Figure 3

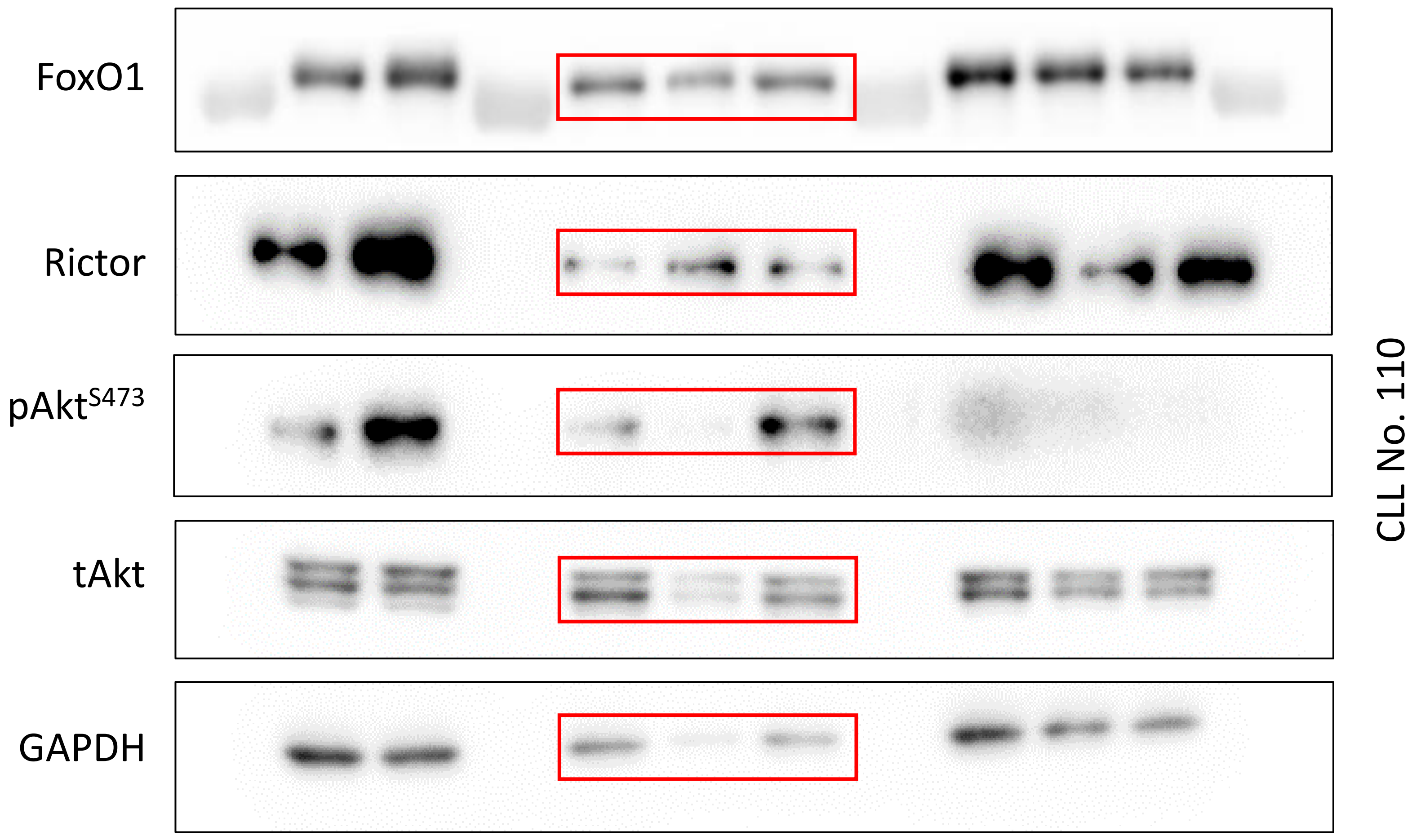

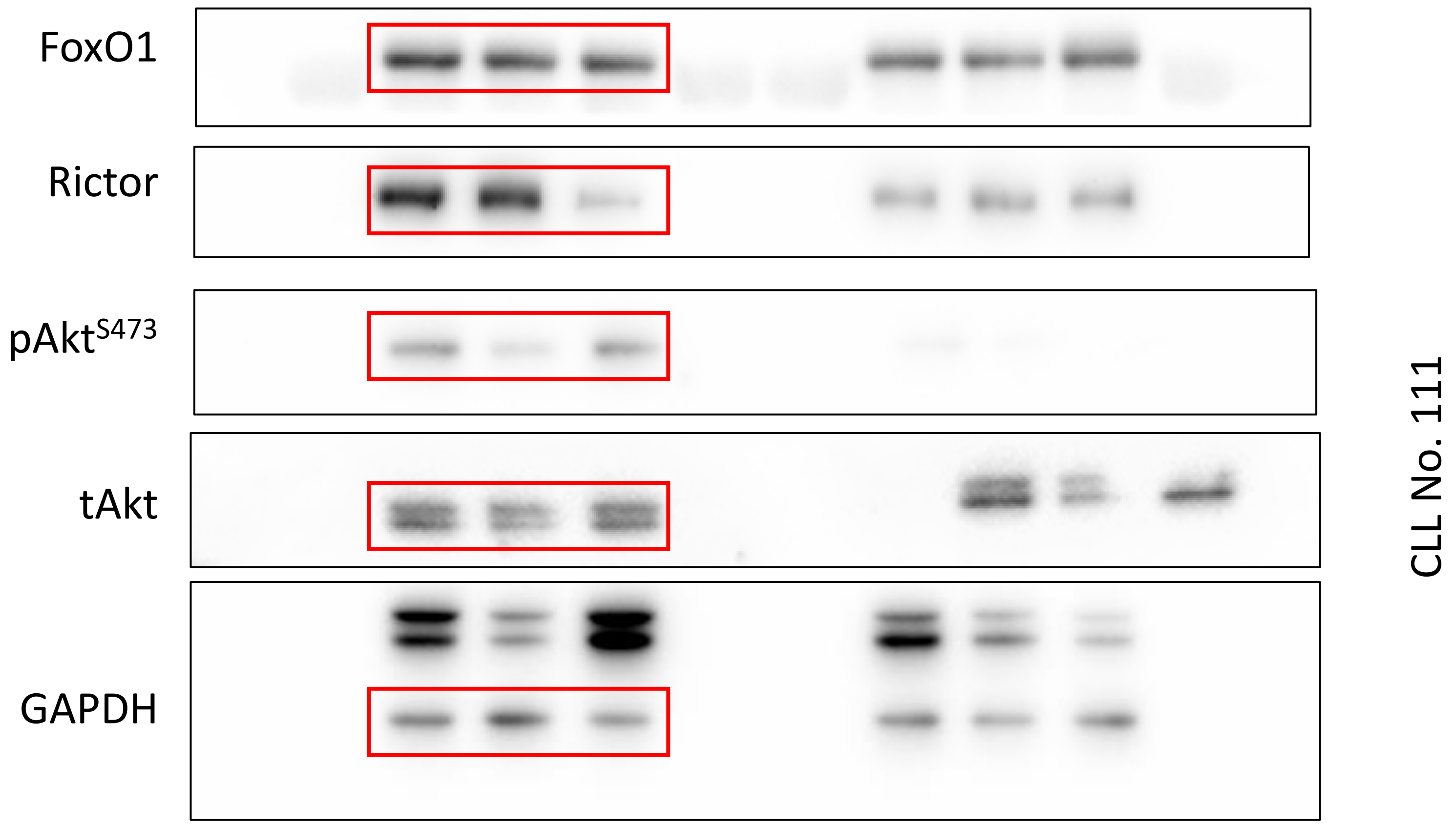

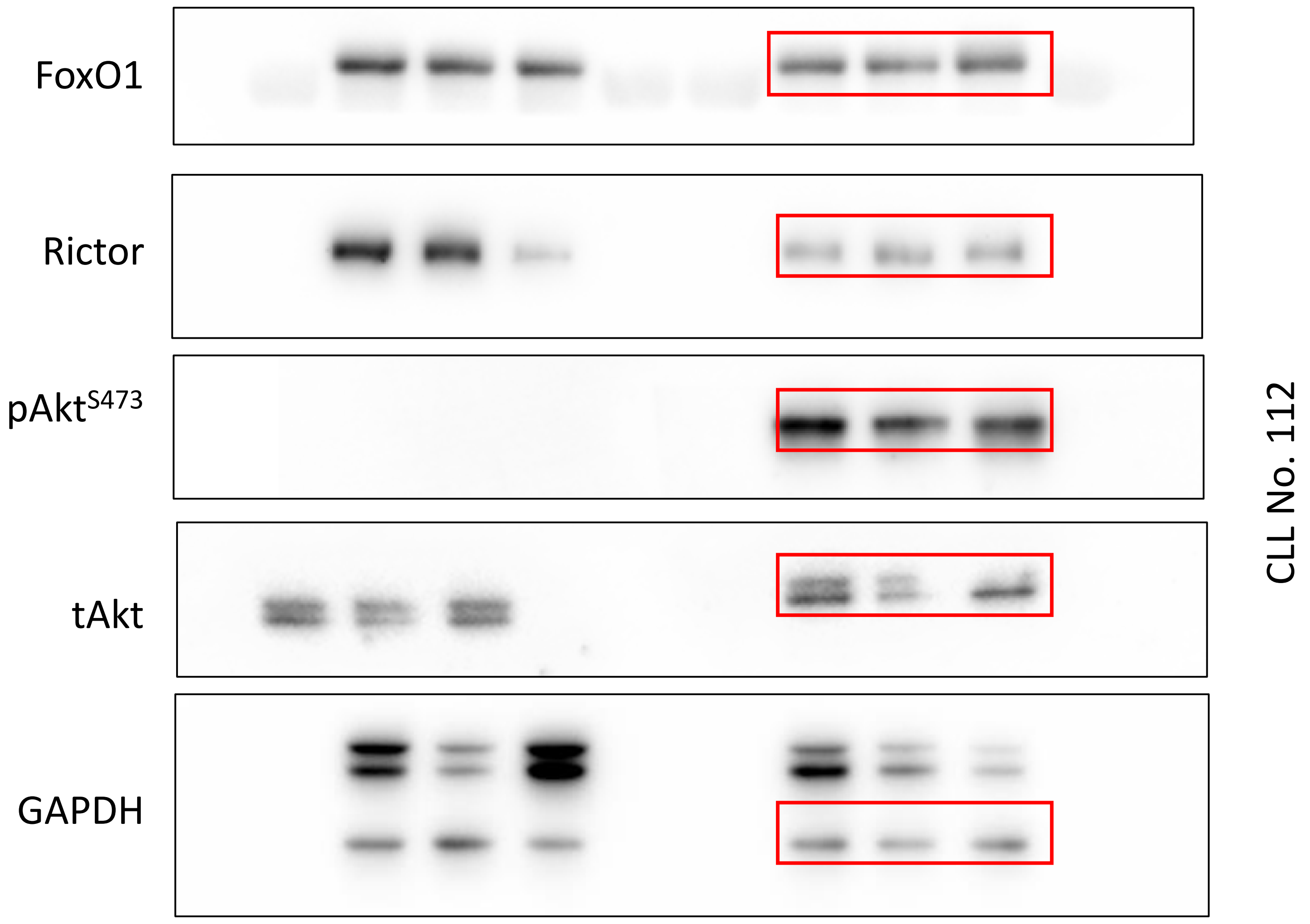

Full unedited gels for Supplemental Figure 6A

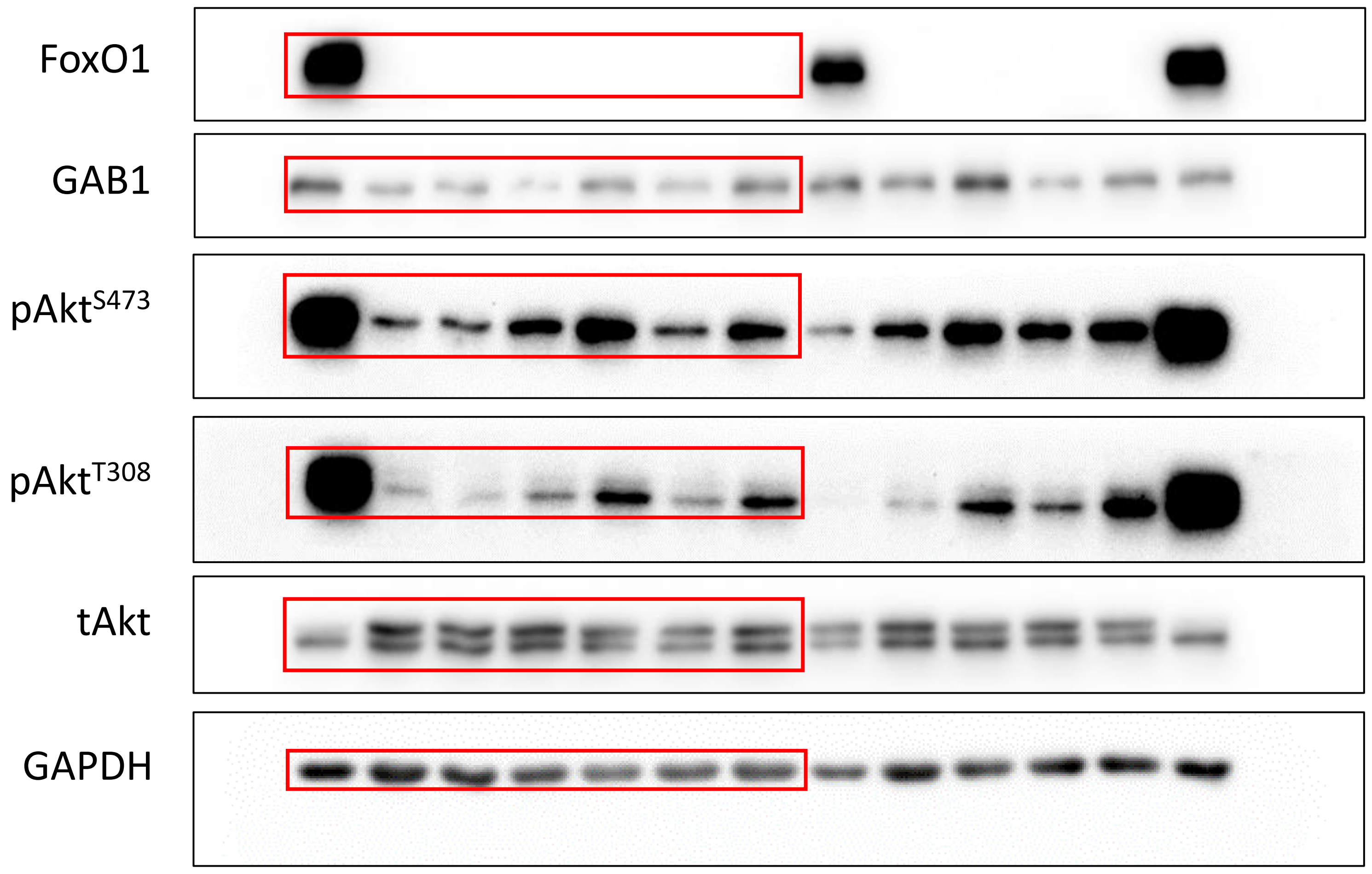

Full unedited gels for Supplemental Figure 6B

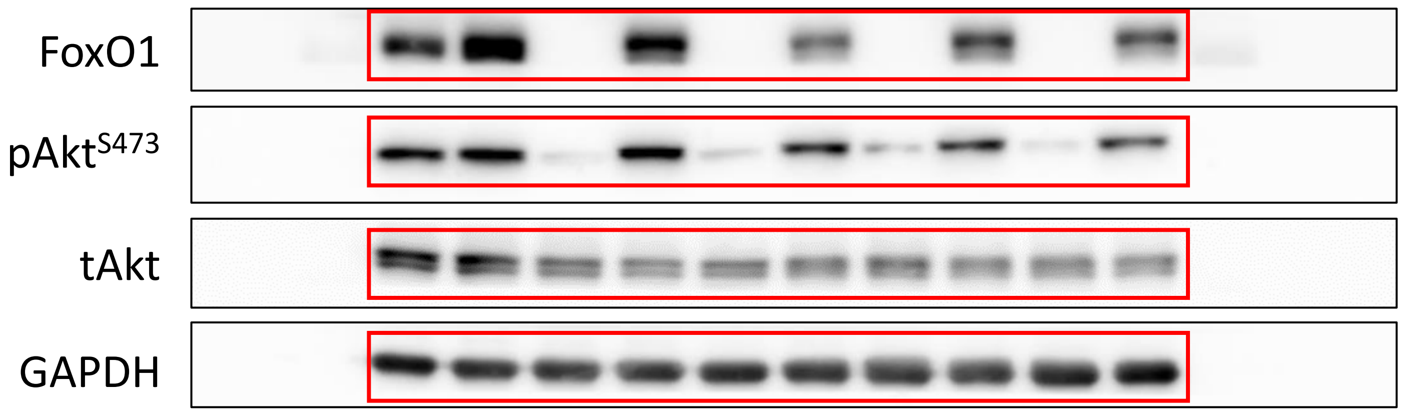

Full unedited gels for Supplemental Figure 6C

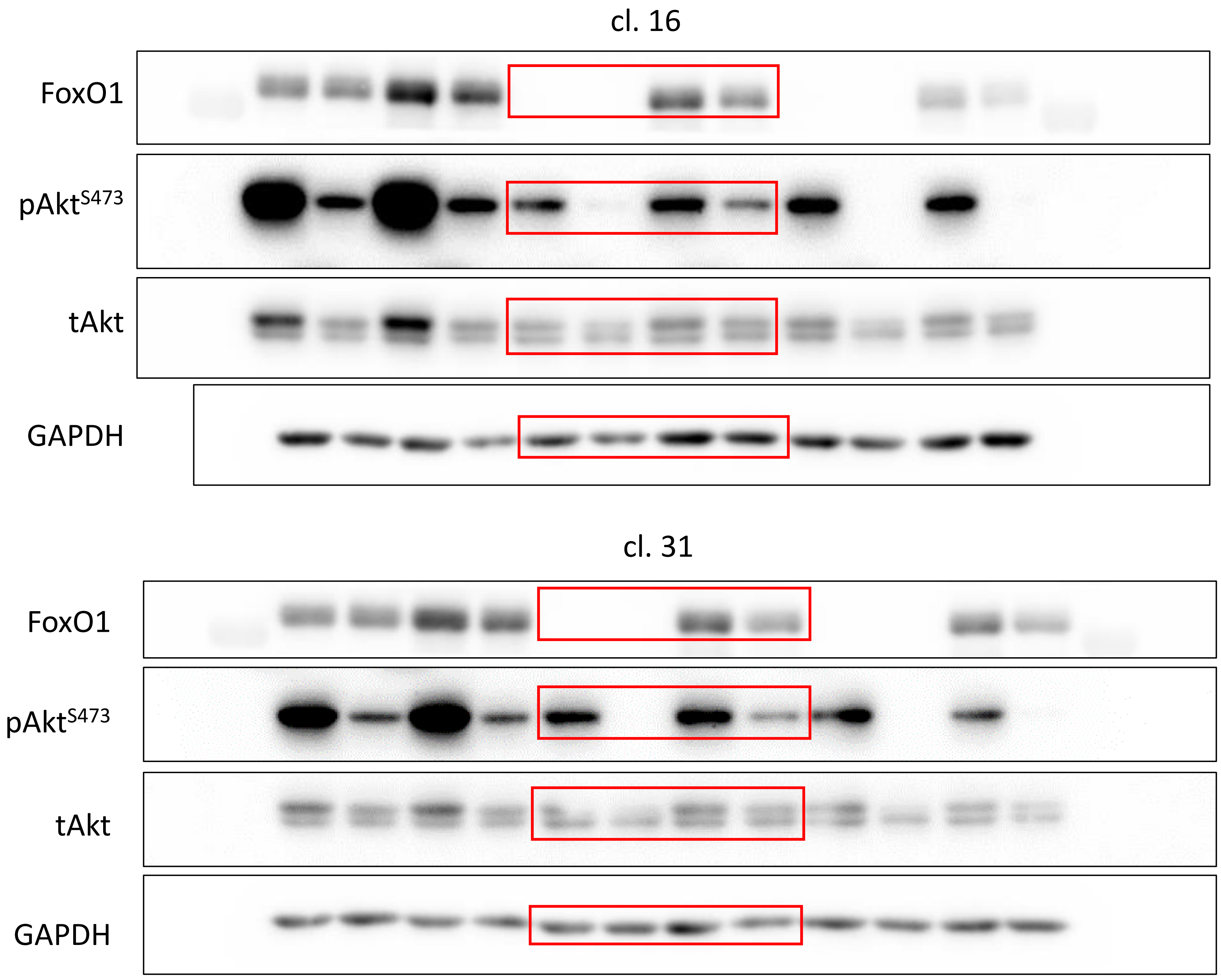

Full unedited gels for Supplemental Figure 7Bi

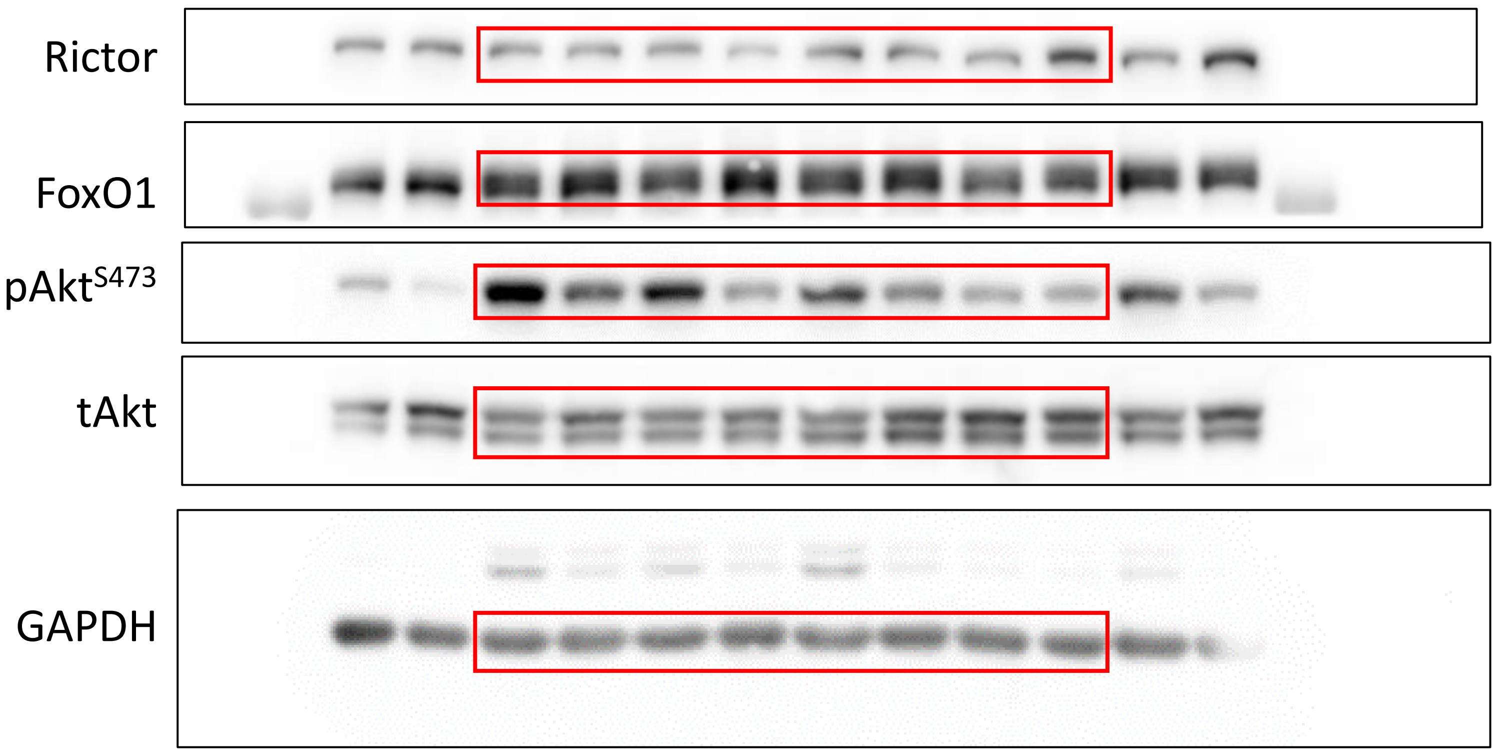

Full unedited gels for Supplemental Figure 7Bii

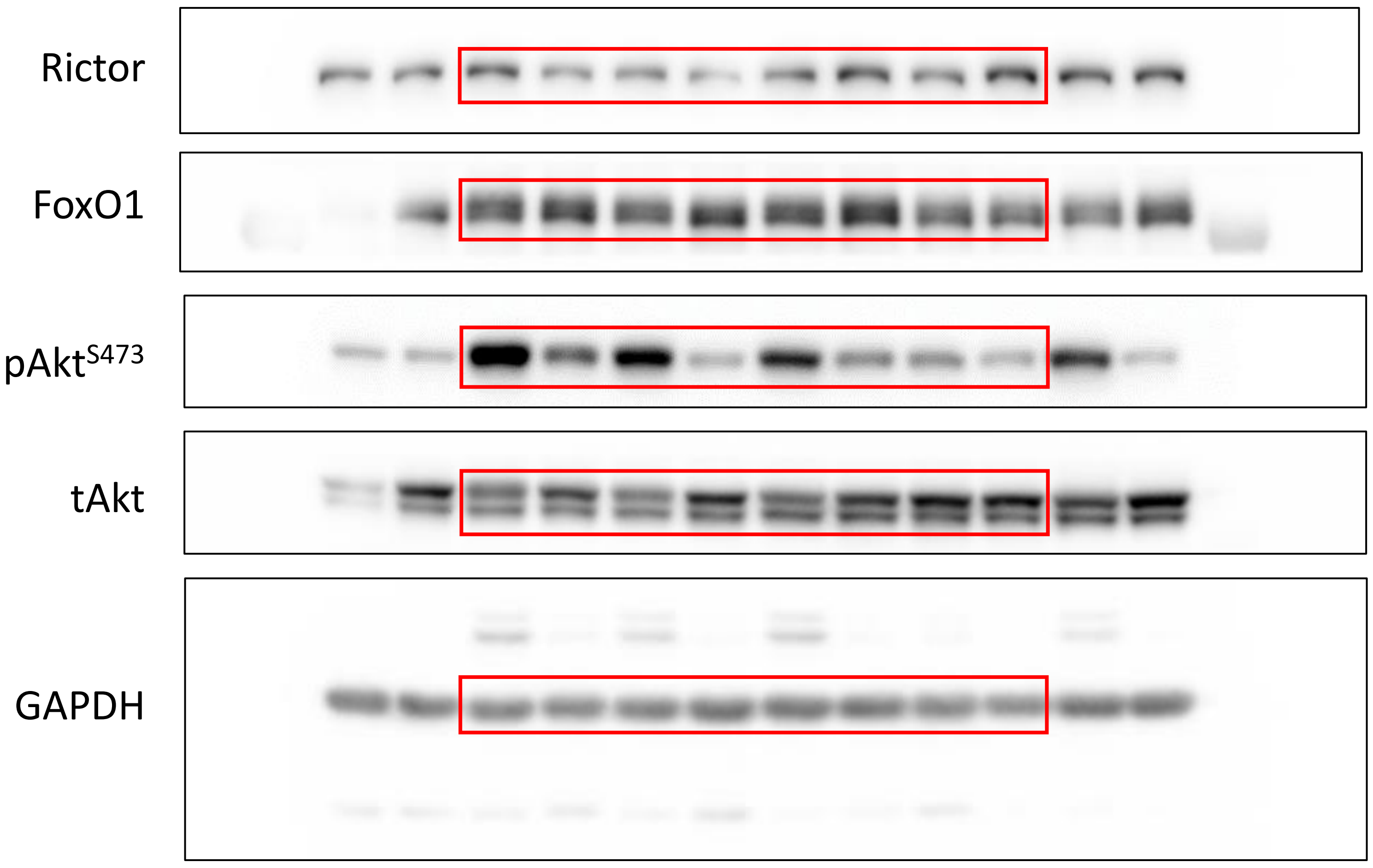

Full unedited gels for Supplemental Figure 7C

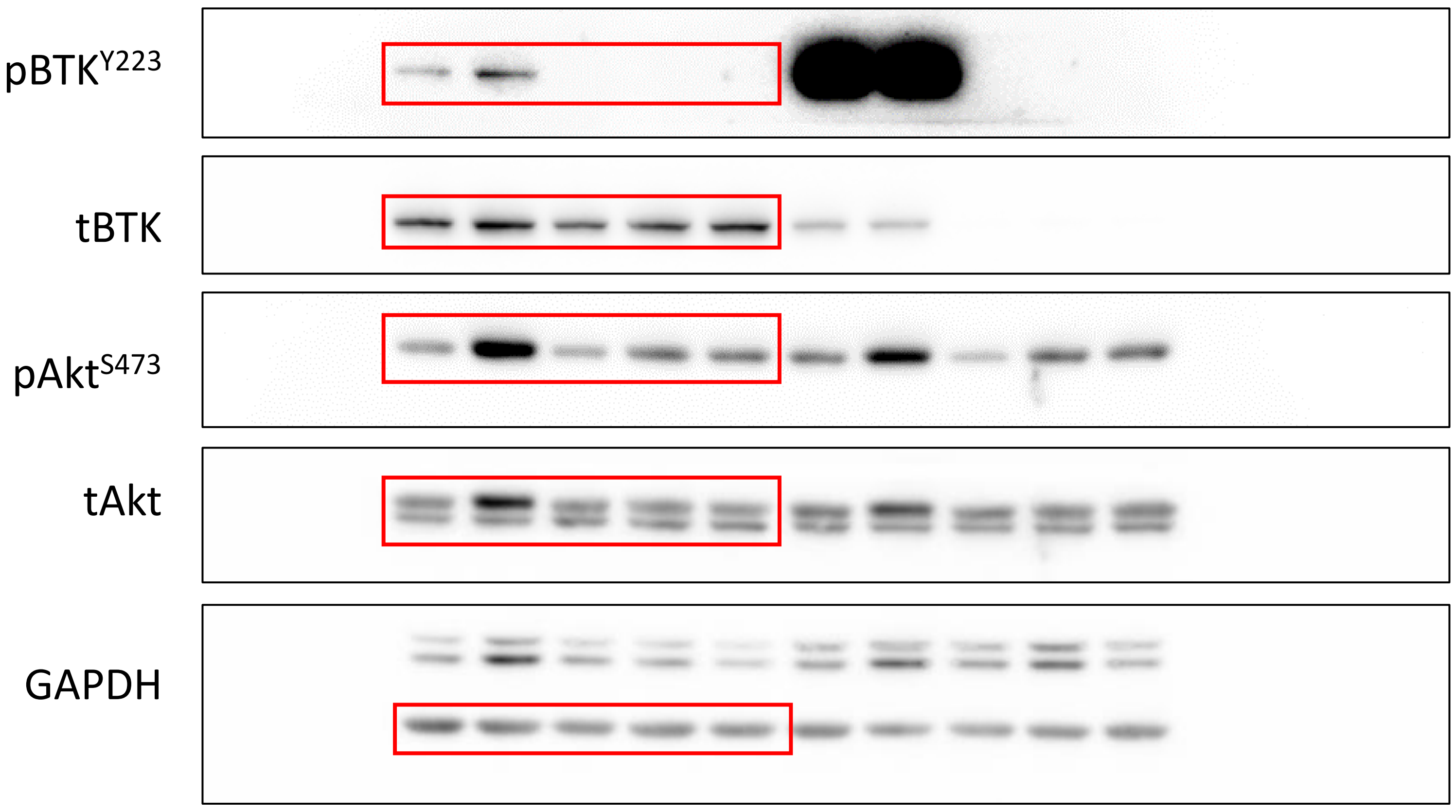

Full unedited gels for Supplemental Figure 9B

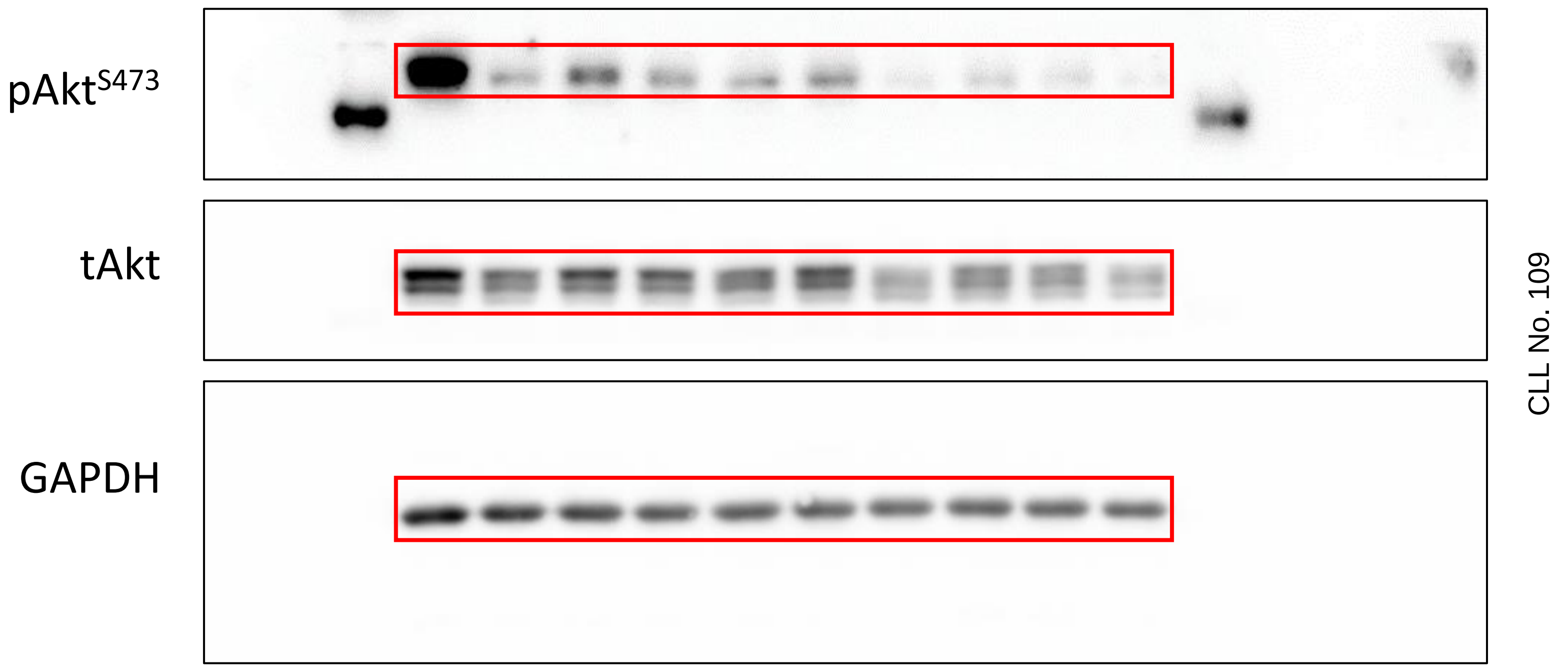

Full unedited gels for Supplemental Figure 12

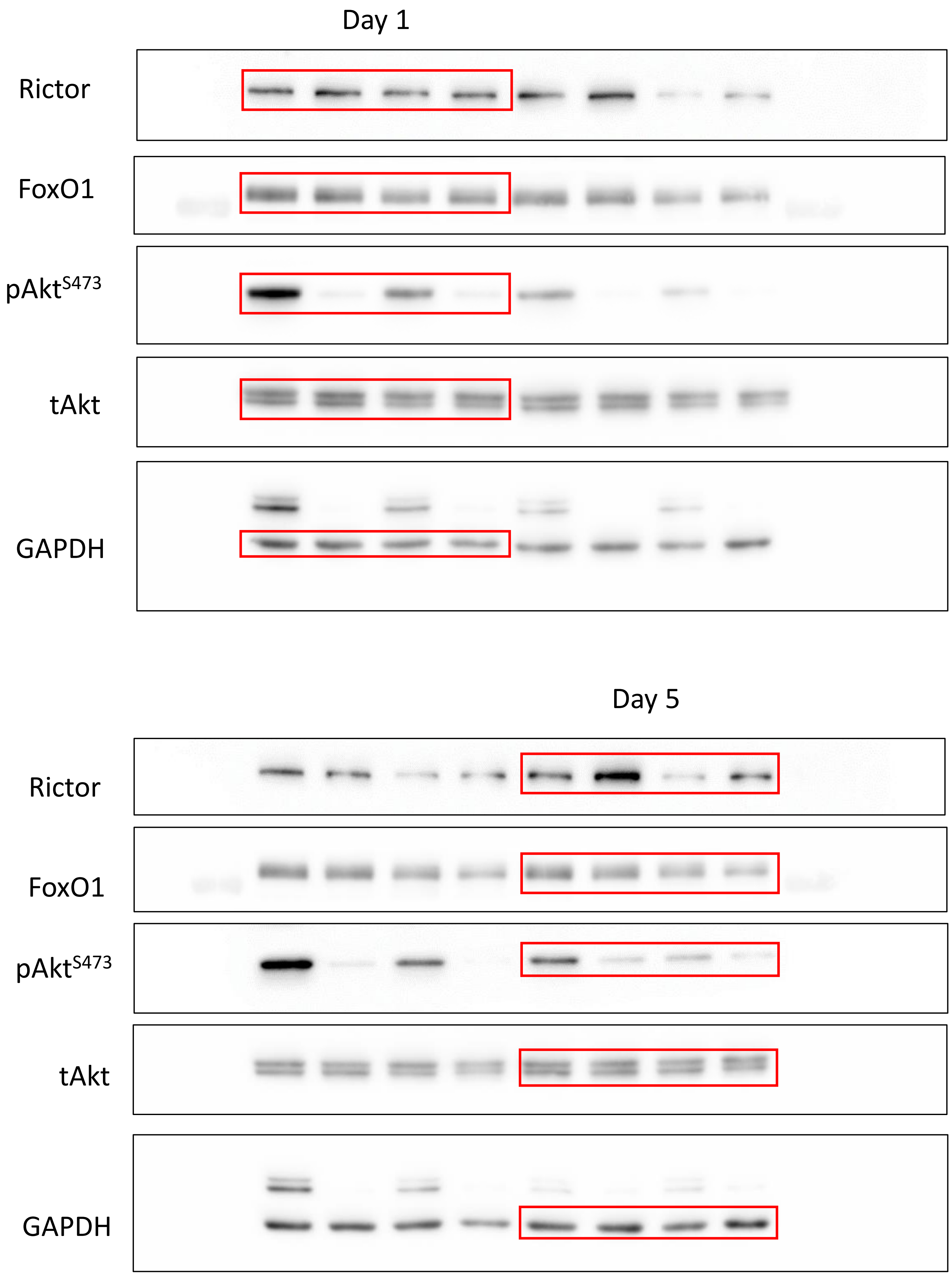

Full unedited gels for Supplemental Figure 13

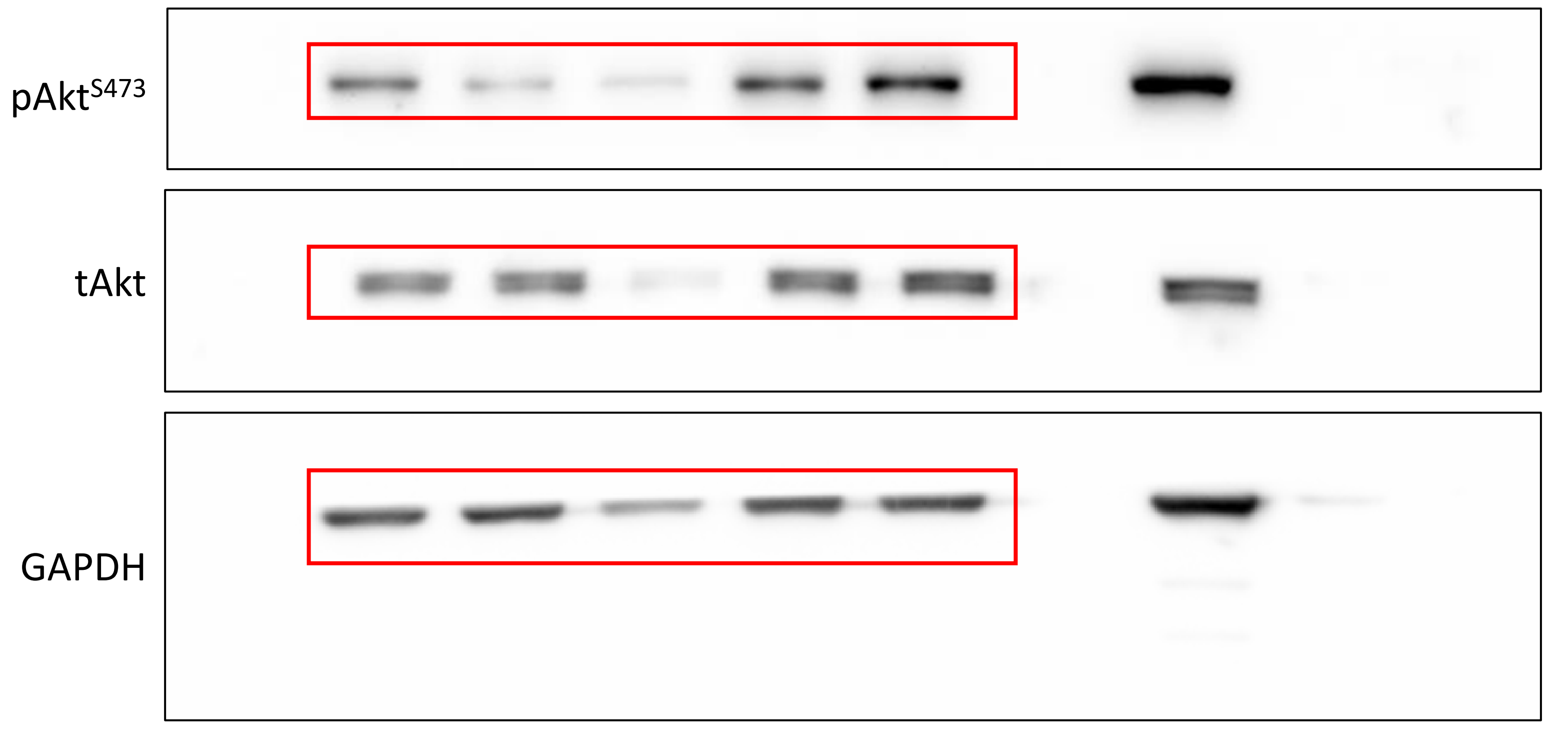

Supplement: Unedited blot and gel images [file jci-134-173770-s149.pdf]
